# Supplementary material for: Resource use, availability and cost in the provision of critical care in Tanzania: a systematic review
Source: BMJ Open. 2022 Nov 22;12(11):e060422. doi: 10.1136/bmjopen-2021-060422 (PMC9684998; doi:10.1136/bmjopen-2021-060422)
Supplement: Supplementary data [file bmjopen-2021-060422supp001.pdf]

SUPPLEMENTARY MATERIAL

Supplementary 1: Key concepts and search terms identified for the search.

Databases: Embase, Medline and Global Health
Search platform: Ovid

Database: Embase Classic+Embase <1947 to 2020 November 18> , Global Health <1973 to 2020 Week 45>, Ovid MEDLINE(R) ALL <1946 to November 17, 2020>
Search Strategy:
----
1 exp intensive care/ or hospital care.ab,ti. or artificial ventilation/ or early goal-directed therapy/ or intensive care nursing/ or newborn intensive care/ or patient monitoring/ or pediatric advanced life support.ab,ti. or emergency.ab,ti. or urgent care.ab,ti. (1538827)
2 (hospital patient or inpatient).ab,ti. (248658)
3 exp hospital patient/ (188072)
4 exp "health care cost"/ or "cost"/ or health economics/ or "drug cost"/ or health care financing/ or "hospital cost"/ or nursing cost.ab,ti. or cost.ab,ti. or costs.ab,ti. (1697788)
5 exp tanzania/ or tanzania.ab,ti. (45585)
6 exp Health Care Costs/ or exp Health Expenditures/ or (cost* or ((health care or health-care or healthcare or medical) adj3 expenditure*) or expense* or hospital resources).ti,ab. or (((resource* or "health service") adj3 ("use" or utilisation or utilization)) or ((financial or economic) and (burden or impact or consequence)) or spending or out of pocket or out-of-pocket or OOP or insurance or absenteeism or (productivity and (loss or lost)) or "economic modelling").ab,ti. (2248416)
7 artificial ventilation.ab,ti. (7728)
8 (malaria or tb or tuberculosis or diagnostics or laboratory or lab or hiv).ab,ti. (3253274)
9 (general care or general ward care or general inpatient care or hospital care).ab,ti. (27507)
10 1 or 2 or 3 or 7 or 8 or 9 (4988953)
11 (economic evaluation or cost effective).ab,ti. (264378)
12 4 or 6 or 11 (2320354)
13 5 and 10 and 12 (1905)
14 limit 13 to yr="2010 -Current" (1295)
15 remove duplicates from 14 (792)

Kazibwe J, *et al. BMJ Open* 2022; 12:e060422. doi: 10.1136/bmjopen-2021-060422

Google Scholar

"Intensive care" OR "Critical care" OR "Critical illness" OR "Resource use" AND "Tanzania"

Supplementary 2: Inclusion and exclusion criteria

Inclusion criterion

For a study to be included it should fulfil the following criterion

- Published in English in a peer reviewed journal
- Reports forms or types of critical care offered, critical care services offered and or costs and resources used in the provision of care in Tanzania
- Includes costs from a provider perspective
- Articles published from 2010

Exclusion criterion

Studies will be excluded in case of the following;

- Published in any other languages except English
- Studies carried out outside Tanzania
- Studies with costs estimated from patient perspective
- Studies without full text versions
- Studies published before 2010

Supplementary 3: Study Characteristics (N=31)]

Article	Disease area	Intervention	Study Type	Purpose Type	Geographical area covered	Hospital Type	Facility/hospital Level	Source of data
**Studies reporting on costs**								
Baynes et al 2019	Obstetrics	Providing care to women that have had an abortion	Cross sectional	Cost Analysis	Mwanza, Arusha and Zanzibar	NGO & Public	All	Records and interviews
Chris et al 2013	General	Health services	Cross sectional	Cost Analysis	National	All	All	Survey
Kabadi et al 2013	Stroke	Providing stroke care	Longitudinal	Cost Analysis	Mbeya and Dar es Salaam	NGO & Public	Tertiary referral	Survey and project data
Mengistu et al 2019	Direct obstetric complications	Emergency Obstetric and Neonatal Care	Cross sectional	Cost Analysis	Kigoma	Public	Tertiary, district hospital and primary care	Programme & hospital data

Kazibwe J, *et al. BMJ Open* 2022; 12:e060422. doi: 10.1136/bmjopen-2021-060422

**Article**	**Disease area**	**Intervention**	**Study Type**	**Purpose Type**	**Geographical area covered**	**Hospital Type**	**Facility/hospital Level**	**Source of data**
Mosha et al 2010	Malaria	Diagnostic tools and drugs	Cross sectional	Cost analysis	Kilimanjaro region and Korogwe District, Tanga region	Public	District and dispensaries	Survey
Riewpaiboon et al 2014	Typhoid	Typhoid treatment/care	Longitudinal	Cost Analysis	Pemba and Zanzibar	Public	District hospital	Record review and interviews
Sicuri et al 2011	Malaria	Malaria treatment/care	Longitudinal	Cost Analysis	Not specified	Not specified	Not specified	Survey
Chaudhury et al 2016	Neonatal care	Birth attendants training on	Cross sectional	Cost Analysis	Mbeya	NGO & Public	All	Survey

Kazibwe J, *et al. BMJ Open* 2022; 12:e060422. doi: 10.1136/bmjopen-2021-060422

Article	Disease area	Intervention	Study Type	Purpose Type	Geographical area covered	Hospital Type	Facility/hospital Level	Source of data
		neonatal resuscitation						
Penno et al 2015	Antimicrobial resistance	Surveillance for Bloodstream Infections for Sepsis Management in low resource setting	Modelling	Economic evaluation	Northern Tanzania	Not specified	Not specified	Laboratory records
Githang'a et 2020	Oncology	Oncology treatment: e.g. chemotherapy, off site	Cross-sectional	Economic evaluation	Mwanza	NGO	Tertiary referral	Hospital data & interviews

**Article**	**Disease area**	**Intervention**	**Study Type**	**Purpose Type**	**Geographical area covered**	**Hospital Type**	**Facility/hospital Level**	**Source of data**
		radiotherapy, surgery						
Guerriero et al 2010	Blood transfusion	Use of Tranexamic acid as a replacement for blood transfusion	Modelling	Economic evaluation	National	Not specified	Not specified	Literature

Kazibwe J, *et al. BMJ Open* 2022; 12:e060422. doi: 10.1136/bmjopen-2021-060422

**Article**	**Disease area**	**Intervention**	**Study Type**	**Purpose Type**	**Geographical area covered**	**Hospital Type**	**Facility/hospital Level**	**Source of data**
Kimaro et al 2017	Advanced HIV	Providing antiretroviral therapy services to HIV-infected individuals presenting with advanced HIV disease	Cohort	Economic evaluation	Dar es Salaam	Public	Primary health center	Patient survey, health care worker diaries
Phillips et al 2015	Malaria	Malaria Rapid Diagnostic test (mRDT)	Modelling	Economic evaluation	National	All	All	Local program/ records

**Article**	**Disease area**	**Intervention**	**Study Type**	**Purpose Type**	**Geographical area covered**	**Hospital Type**	**Facility/hospital Level**	**Source of data**
Shayo et al 2017	Tuberculosis	Isoniazid preventive therapy	Cohort	Economic evaluation	Dar es Salaam	NGO, Public and private	Tertiary and primary care	Records and interviews
Wilson et al 2020	Neonatal care	Birth attendants training on neonatal resuscitation (59 participants)	Cross sectional	Financial planning	Zanzibar	Not specified	Not specified	Survey
**Studies reporting on forms and resource availability in critical care**								
Baker et al 2011	Critical care	Emergency and critical care	Cross sectional	Others (structure and resources	National	All	Tertiary, district, and primary care	Survey

Kazibwe J, *et al. BMJ Open* 2022; 12:e060422. doi: 10.1136/bmjopen-2021-060422

**Article**	**Disease area**	**Intervention**	**Study Type**	**Purpose Type**	**Geographical area covered**	**Hospital Type**	**Facility/hospital Level**	**Source of data**
				available for emergency and critical care)				
Chalya et al 2011	Trauma	Trauma care	Cross sectional	Others (describe patient characteristics and health outcomes)	Mwanza	NGO	Referral hospital	Survey
Mkoka et al 2014	Obstetrics	Emergency obstetric care	Cross sectional	Others (availability	Kongwa	Public	Primary healthcare	Interviews

Kazibwe J, *et al. BMJ Open* 2022; 12:e060422. doi: 10.1136/bmjopen-2021-060422

**Article**	**Disease area**	**Intervention**	**Study Type**	**Purpose Type**	**Geographical area covered**	**Hospital Type**	**Facility/hospital Level**	**Source of data**
				of medical supplies)				
Mtango et al 2019	Critical care	Critical care	Cross sectional	Others (doctors' experiences of ICU referral)	Dar es salaam	Public	Tertiary referral	Interviews
Murthy et al 2015	Critical care	N/A	Review	Others (estimate the current ICU capacity)	Ifakara, Dar es Salaam and Mwanza	NGO & Public	Tertiary referral	Literature
Mwandri et al 2020	Trauma	Baseline assessment of	Cross sectional	Others (assessing	Mbeya and Dar es Salaam	NGO & Public	Tertiary referral	Hospital registry data

Article	Disease area	Intervention	Study Type	Purpose Type	Geographical area covered	Hospital Type	Facility/hospital Level	Source of data
		trauma care provision		burden and care process)				
Mwiga & Monaghan 2020	Respiratory illness	Difficult airway trolley	Case study	Others (development of difficulty airway trolley)	Mbeya	NGO	Tertiary referral	Difficult airway trolley
Nicks et al 2012	Emergence medicine	N/A	Case study	Others (structure of health system and care processes)	National	All	All	Literature

Article	Disease area	Intervention	Study Type	Purpose Type	Geographical area covered	Hospital Type	Facility/hospital Level	Source of data
Papali et al 2019	Critical care	N/A	Review	Others (structure and organisation of adult ICU)	N/A	All	All	Literature
Penoyar et al 2012	Surgery	Surgical care	Cross sectional	Others (capacity of first referral hospitals)	National	All	District, regional and referral	Survey
Rajaguru et al 2019	Surgery	Surgery	Cross sectional	Others (operation and financing of a referral hospital)	Mbeya	NGO	Tertiary referral	Record review

**Article**	**Disease area**	**Intervention**	**Study Type**	**Purpose Type**	**Geographical area covered**	**Hospital Type**	**Facility/hospital Level**	**Source of data**
Reynolds et al 2012	General (particular injuries, pneumonia and psychosis)	Emergency care	Cross sectional	Others (Barriers to emergency care)	National	Public	Tertiary referral	Interview
Sawe et 2016	General	Emergency treatment	Cross sectional	Others (clinical presentation and resource utilization)	Dar es Salaam	Public	Tertiary referral	Hospital record
Staton et al 2017	Traumatic Brain Injury	Trauma care	Cross sectional	Others (describe	Kilimanjaro	NGO	Referral Hospital	Survey

Article	Disease area	Intervention	Study Type	Purpose Type	Geographical area covered	Hospital Type	Facility/hospital Level	Source of data
				quality of care for TBI)				
Sylvanus et al 2019	Renal failure	Renal failure treatment and management	Cohort	Others (renal failure case management)	Dar es Salaam	Public	Tertiary referral	Survey
Zimmerman et al 2020	Acute traumatic brain injury	Quantifying the effect on outcomes from delays in the care of trauma	Cross sectional	Others (association of hospital delays and poor outcomes)	Moshi	NGO	Tertiary referral	Hospital data

Kazibwe J, *et al. BMJ Open* 2022; 12:e060422. doi: 10.1136/bmjopen-2021-060422

Supplementary 4: Availability of resources required for critical care provision

**Resources**	**Study**			
	Penoyar et al 2012 (N=40 hospitals)	*Staton et al 2017 (patients; N=893)*	*Zimmerman et al 2020 (patients; N=3209)*	*Baker et al 2013 (N=10 hospitals)*
Availability of oxygen when needed	42%	22.9%	20.1%	
Oxygen concentrators	75%			
Mask and tubing to connect to oxygen supply	46%			
Pulse oximetry	13%			70%
Oxygen cylinders	35%			
Mechanical ventilators				40%
Oropharyngeal airway-adult	42%			60%
Oropharyngeal airway-paediatric	21%			
Personal protective wear (gloves)	90%			90%
Personal protective wear (aprons)	81%			
Personal protective wear (face masks)	69%			
Personal protective wear (eye protection)	40%			
Consistent running water	37.5%			100%
Consistent running electricity	37.5%			50%
Staff trained in critical care				20%

Supplementary 5: Appraisal Checklist

Kazibwe J, *et al. BMJ Open* 2022; 12:e060422. doi: 10.1136/bmjopen-2021-060422

**Study ID**	**Author**	**#1 Question(s)**	**#2 Perspective**	**#3 Time horizon**	**#4 Relevant inputs**	**#5 Methods for quantities**	**#6 Data source(s)**	**#7 Sample size**	**#8 Discount rate**	**#9 Sensitivity analysis**	**#10 Costs reporting**
1	Githang'a et 2020	✓	✓	(✓)	✓	✓	✓		✓	✓	✓
2	Mengistu et al 2019	✓	✓	✓	✓	✓	✓			X	✓
3	Wilson et al 2020	✓	✓	**X**	✓	✓	✓			X	✓
4	Kimaro et al 2017	✓	✓	✓	✓	✓	✓			✓	✓
5	Shayo et al 2017	✓	✓	✓	✓	✓	✓			✓	✓
6	Phillips et al 2015	✓	✓	✓	✓	✓	✓			X	✓
7	Penno et al 2015	✓	✓	X	✓	✓	✓			✓	✓
8	Kabadi et al 2013	✓	✓	✓	✓	✓	✓			X	✓
9	Sicuri et al 2011	✓	✓	X	✓	✓	✓			✓	✓

Kazibwe J, *et al. BMJ Open* 2022; 12:e060422. doi: 10.1136/bmjopen-2021-060422

10	Guerriero et al 2010	✓	✓	X	✓	✓	✓			✓	✓
11	Baynes et al 2019	✓	✓	(✓)	✓	✓	✓			✓	✓
12	Riewpaiboon et al 2014	✓	✓	(✓)	✓	✓	✓			✓	✓
13	Sumona et al 2016	✓	✓	(✓)	✓	✓	✓			✓	✓
14	James et al 2013	✓	✓	✓	✓	✓	✓			X	✓

Key: ✓ addressed; (✓) partially addressed; X Not addressed

Supplementary 6: Cost of diagnostics

Study	Treatment/disease area	Description	Unit of output	Currency year	Unit cost Price in 2019 (USD)	Unit cost Price in 2019 (TZS)
Mengistu et al 2019	Emergency obstetric care	Laboratory	per programme	2013	9.72	22249.85
Riewpaiboon et al 2014	Typhoid fever	Blood count	Per test	2010	1.64	3751.89
Riewpaiboon et al 2014	Typhoid fever	Blood chemistry	Per test	2010	2.46	5627.84
Kabadi et al 2013	Stroke	Diagnostics for stroke (Blood, ECG, ECHO, CXR) - Rural area	Cost per patient	2005-2006	3.09	7065.07
Kabadi et al 2013	Stroke	CT Scan- rural area	Cost per patient	2005-2006	44.35	101473.18
Kabadi et al 2013	Stroke	Diagnostics for stroke (Blood, ECG, ECHO, CXR) - Urban area	Cost per patient	2005-2006	35.89	82117.17

Kabadi et al 2013	Stroke	CT Scan- urban area	Cost per patient	2005-2006	55.43	126841.48
Kabadi et al 2013	Stroke	Diagnostics for stroke (Blood, ECG, ECHO, CXR) - average	Cost per patient	2005-2006	13.34	30519.33
Kabadi et al 2013	Stroke	CT Scan- average	Cost per patient	2005-2006	47.81	109400.77
Baird et al 2014	Sepsis management	Integrated Management of Adolescent and Adult Illness assessment	Per test	2011	1.17	2677.59
Baird et al 2014	Sepsis management	Lab assessment for sepsis- Negative blood culture	Per test	2011	13.09	29952.25
Baird et al 2014	Sepsis management	Lab assessment for sepsis- positive blood culture	Per test	2011	23.00	52633.02
Baird et al 2014	Sepsis management	Lab assessment for sepsis- ID and susceptibilities positive blood culture	Per test	2011	82.55	188901.34
Phillips et al 2015	Malaria	Malaria rapid diagnostic test	Per test	2010	14.92	34142.23
Shayo et al 2017	TB	Sputum microscopy	Per test	2012	2.16	4945.33
Shayo et al 2017	TB	Suptum culture	Per test	2012	10.83	24778.14
Kimaro et al 2017	HIV - ART provision	CD4 Count test	Per test	2012	17.74	40592.88
Kimaro et al 2017	HIV - ART provision	Alanine aminotransferase (ALAT) test	Per test	2012	0.99	2266.61
Kimaro et al 2017	HIV - ART provision	creatinine test	Per test	2012	0.35	798.46
Kimaro et al 2017	HIV - ART provision	Haemoglobin test	Per test	2012	0.98	2240.85
Kimaro et al 2017	HIV - ART provision	Random blood glucose (RBG) test	Per test	2012	0.96	2189.34
Kimaro et al 2017	HIV - ART provision	Venereal disease reference laboratory (VDRL) test	Per test	2012	2.13	4868.05
Kimaro et al 2017	HIV - ART provision	Pregnancy test	Per test	2012	0.60	1365.12
Kimaro et al 2017	HIV - ART provision	Full blood count (FBC) test	Per test	2012	2.30	5254.41

Kimaro et al 2017	HIV - ART provision	Serum cryptococcal meningitis (CRAG) test	Per test	2012	4.46	10199.73
Kimaro et al 2017	HIV - ART provision	Xpert test	Per test	2012	21.47	49118.41
Kimaro et al 2017	HIV - ART provision	Collection and processing of cerebral spinal fluid	Per test	2012	18.34	41957.99
Kimaro et al 2017	HIV - ART provision	Chest X-ray	Per test	2012	3.58	8190.70
Kimaro et al 2017	HIV - ART provision	Smear microscopy test	Per test	2012	2.04	4662.00
Kimaro et al 2017	HIV - ART provision	Sputum culture (Lowenstein Jensen) for TB	Per test	2012	7.70	17617.72

Supplementary 7: Cost of human resources

**Study**	**Description**	**Unit of output**	**Currency year**	**Unit cost Price in 2019 (USD)**	**Unit cost Price in 2019 (TZS)**
Mengistu et al 2019	Total personnel for Emergency obstetric care	per patient	2013	103.01	235,711.74
Baynes et al 2019	Cost of Obstetrician/gynecologist on all complications	Per patient	2016	10.00	22,884.06
Baynes et al 2019	Cost of Anesthesiologist on all obstetric complications	Per patient	2016	0.00	0.00

Kazibwe J, *et al. BMJ Open* 2022; 12:e060422. doi: 10.1136/bmjopen-2021-060422

Baynes et al 2019	Cost Anesthetist on all obstetric complications	Per patient	2016	2.23	5,112.40
Baynes et al 2019	Cost of Anesthetist Assistant on all obstetric complications	Per patient	2016	1.81	4,138.61
Baynes et al 2019	Cost of doctor/ medical officer on all obstetric complications	Per patient	2016	15.75	36,030.22
Baynes et al 2019	Cost of assistant medical officer on all obstetric complications	Per patient	2016	5.00	11,442.03
Baynes et al 2019	Cost of clinical officer on all obstetric complications	Per patient	2016	0.85	1,947.58
Baynes et al 2019	Cost of registered nurse or principal enrolled nurse on obstetric all complications	Per patient	2016	9.79	22,397.16
Baynes et al 2019	Cost of enrolled nurse or midwife on all obstetric complications	Per patient	2016	43.62	99,813.44
Baynes et al 2019	Cost of medical attendant on all obstetric complications	Per patient	2016	6.06	13,876.50
Baynes et al 2019	Cost of laboratory technician on all complications	Per patient	2016	3.19	7,303.42
Baynes et al 2019	Cost of laboratory assistant on all complications	Per patient	2016	1.70	3,895.16

Kazibwe J, *et al. BMJ Open* 2022; 12:e060422. doi: 10.1136/bmjopen-2021-060422

Baynes et al 2019	Cost of Sonographer on all obstetric complications	Per patient	2016	2.87	6,573.08
Baynes et al 2019	Cost of pharmacist on all complications	Per patient	2016	2.55	5,842.74
Baynes et al 2019	Cost of drug dispenser on all complications	Per patient	2016	1.06	2,434.47
Baynes et al 2019	Average personnel costs at primary care level facilities for all complications	Per patient	2016	14.28	32,670.64
Baynes et al 2019	Average personnel care costs at district hospitals for all complications	Per patient	2016	8.97	20,522.62
Baynes et al 2019	Average personnel costs at Regional hospital for all complications	Per patient	2016	30.14	68,968.66
Baynes et al 2019	Average personnel costs for all complications across any level of hospital	Per patient	2016	19.16	43,844.88
Shayo et al 2017	Assistant medical officer	Monthly salary	2012	638.96	1,462,064.84
Shayo et al 2017	Nurse	Monthly salary	2012	313.34	716,994.91
Shayo et al 2017	Health Attendant	Monthly salary	2012	440.41	1,007,738.83
Shayo et al 2017	Assistant Nurse	Monthly salary	2012	338.60	774,793.40

Kazibwe J, *et al. BMJ Open* 2022; 12:e060422. doi: 10.1136/bmjopen-2021-060422

Shayo et al 2017	Nurse Counsellors	Monthly salary	2012	819.44	1,875,051.02
Shayo et al 2017	Medical Doctors	Monthly salary	2012	1473.56	3,371,810.40
Shayo et al 2017	Medical Specialists	Monthly salary	2012	2721.79	6,228,019.13
Shayo et al 2017	Laboratory Scientific Officer	Monthly salary	2012	1106.07	2,530,924.80
Shayo et al 2017	Laboratory technician	Monthly salary	2012	819.44	1,875,051.02
Shayo et al 2017	Pharmacist	Monthly salary	2012	1106.07	2,530,924.80
Shayo et al 2017	Pharmacy technician	Monthly salary	2012	819.44	1,875,051.02

Kazibwe J, *et al. BMJ Open* 2022; 12:e060422. doi: 10.1136/bmjopen-2021-060422
