# Supplementary material for: Resource use, availability and cost in the provision of critical care in Tanzania: a systematic review
Source: BMJ Open. 2022 Nov 22;12(11):e060422. doi: 10.1136/bmjopen-2021-060422 (PMC9684998; doi:10.1136/bmjopen-2021-060422)
Supplement: Author's manuscript [file bmjopen-2021-060422.draft_revisions.pdf]

BMJ Open

BMJ Open is committed to open peer review. As part of this commitment we make the peer review history of every article we publish publicly available.

When an article is published we post the peer reviewers' comments and the authors' responses online. We also post the versions of the paper that were used during peer review. These are the versions that the peer review comments apply to.

The versions of the paper that follow are the versions that were submitted during the peer review process. They are not the versions of record or the final published versions. They should not be cited or distributed as the published version of this manuscript.

BMJ Open is an open access journal and the full, final, typeset and author-corrected version of record of the manuscript is available on our site with no access controls, subscription charges or pay-per-view fees (http://bmjopen.bmj.com).

If you have any questions on BMJ Open's open peer review process please email
info.bmjopen@bmj.com

BMJ Open

Resource use, availability and cost in the provision of critical care in Tanzania: A systematic review

Journal:	*BMJ Open*
Manuscript ID	bmjopen-2021-060422
Article Type:	Original research
Date Submitted by the Author:	28-Dec-2021

Complete List of Authors:	Kazibwe, Joseph; London School of Hygiene & Tropical Medicine, Global Health and Development<br>Shah, Hiral A.; Imperial College London, Department of Infectious Disease Epidemiology; Center for Global Development<br>Kuwawenaruwa, A; Ifakara Health Institute, Health System Impact Evaluation and Policy Unit<br>Schell, Carl Otto; Karolinska Institutet, Department of Global Public Health; Uppsala University<br>Khalid, Karima; Muhimbili University of Health and Allied Sciences, Department of Anaesthesia and Critical Care<br>Tran, Phuong Bich; University of Antwerp, Department of Family and Population Health<br>Ghosh, Srobana; Center for Global Development, Global Health Department<br>Baker, Tim; London School of Hygiene & Tropical Medicine, Department of Clinical Research; Ifakara Health Institute, Department of Emergency Medicine<br>GUINNESS, LORNA; Center for Global Development

Keywords:	INTENSIVE & CRITICAL CARE, HEALTH ECONOMICS, Health policy < HEALTH SERVICES ADMINISTRATION & MANAGEMENT

SCHOLARONE™
Manuscripts

I, the Submitting Author has the right to grant and does grant on behalf of all authors of the Work (as defined in the below author licence), an exclusive licence and/or a non-exclusive licence for contributions from authors who are: i) UK Crown employees; ii) where BMJ has agreed a CC-BY licence shall apply, and/or iii) in accordance with the terms applicable for US Federal Government officers or employees acting as part of their official duties; on a worldwide, perpetual, irrevocable, royalty-free basis to BMJ Publishing Group Ltd ("BMJ") its licensees and where the relevant Journal is co-owned by BMJ to the co-owners of the Journal, to publish the Work in this journal and any other BMJ products and to exploit all rights, as set out in our licence.

The Submitting Author accepts and understands that any supply made under these terms is made by BMJ to the Submitting Author unless you are acting as an employee on behalf of your employer or a postgraduate student of an affiliated institution which is paying any applicable article publishing charge ("APC") for Open Access articles. Where the Submitting Author wishes to make the Work available on an Open Access basis (and intends to pay the relevant APC), the terms of reuse of such Open Access shall be governed by a Creative Commons licence – details of these licences and which Creative Commons licence will apply to this Work are set out in our licence referred to above.

Other than as permitted in any relevant BMJ Author's Self Archiving Policies, I confirm this Work has not been accepted for publication elsewhere, is not being considered for publication elsewhere and does not duplicate material already published. I confirm all authors consent to publication of this Work and authorise the granting of this licence.

For peer review only

Resource use, availability and cost in the provision of critical care in Tanzania: A systematic review

Joseph Kazibwe,
1. Global Health and Development Department, London School of Hygiene and Tropical Medicine, London UK
2. ORCID - https://orcid.org/0000-0002-8315-1503

Joseph.Kazibwe@lshtm.ac.uk

Hiral A. Shah,
1. MRC Centre for Global Infectious Disease Analysis, Department of Infectious Disease Epidemiology, School of Public Health, Imperial College London, UK.
2. Center for Global Development, London, UK.
3. ORCID - https://orcid.org/0000-0003-0204-451X

August Kuwawenaruwa,
1. Health System Impact Evaluation and Policy Unit, Ifakara Health Institute United Republic of Tanzania
2. ORCID - https://orcid.org/0000-0001-8459-443X

Carl Otto Schell,
1. Department of Global Public Health, Karolinska Institutet, Stockholm, Sweden
2. Centre for Clinical Research Sörmland, Uppsala University, Eskilstuna, Sweden

   ORCID - https://orcid.org/0000-0002-7904-1336

Karima Khalid,
1. Department of Anaesthesia and Critical Care, Muhimbili University of Health and Allied Sciences, Dar es Salaam, United Republic of Tanzania

Phuong Bich Tran,

1. Department of Family and Population Health, University of Antwerp, Antwerpen, Belgium

For peer review only - http://bmjopen.bmj.com/site/about/guidelines.xhtml

ORCID - https://orcid.org/0000-0003-2119-998X

Srobana Ghosh,

1. Global Health Department, Center for Global Development, London, UK

Tim Baker,

1. Department of Clinical Research, London School of Hygiene & Tropical Medicine, UK
2. Ifakara Health Institute, Dar es Salaam, Tanzania
3. Department of Emergency Medicine, Muhimbili University of Health and Allied Sciences, Dar es Salaam, Tanzania
4. Department of Global Public Health, Karolinska Institutet, Stockholm, Sweden

ORCID: 0000-0001-8727-7018

Lorna Guinness

1. Center for Global Development, London, UK

ORCID: https://orcid.org/0000-0002-1013-4200

Key words: Critical care, Costs of critical care, forms of critical care, Resources, Low- and middle-income countries (LMICs), Tanzania

For peer review only

Abstract

Objectives

Critical care is essential in saving lives of critically ill patients, however, provision of critical care across lower resource settings can be costly, fragmented and heterogenous. Despite the urgent need to scale-up the provision of critical care, little is known about its availability and cost. Here, we aim to systematically review and identify reported resource use, availability and costs for the provision of critical care and the nature of critical care provision in Tanzania.

Methods

The systematic review followed the Preferred Reporting Items for Systematic Reviews and Meta-Analyses (PRISMA) guidelines. We searched Medline, Embase and global health databases. We included studies that reported on provision of critical care, cost and availability of resources used in the provision of critical care published after 2010. Costs were adjusted and reported in 2019 USD and TZS using the world bank GDP deflators.

Results

A total 31 studies were found to fulfil the inclusion and exclusion criteria. Critical care identified in Tanzania was categorised into: ICU delivered critical care and non-ICU critical care. The availability of ICU delivered critical care was limited to urban settings whereas non-ICU critical care was found in rural and urban settings. 15 studies reported on the costs of services related to critical care yet no study reported an average or unit cost of critical care. Costs of medication, equipment (e.g. oxygen, PPE), services, and human resources were identified as inputs to specific critical care services in Tanzania.

Conclusion

There is limited evidence on the resource use, availability and costs of critical care in Tanzania. There is a strong need for further empirical research on critical care resources availability, utilization and costs across specialties and hospitals of different level in LMICs

For peer review only - http://bmjopen.bmj.com/site/about/guidelines.xhtml

like Tanzania to inform planning, priority setting and budgeting for critical care services. PROSPERO registration number: CRD42020221923

Strength and limitations of this study

- This study followed the Preferred Reporting Items for Systematic Reviews and Meta-Analyses guidelines thereby being the first systematic review of the literature around the costs and resources used in critical care and forms of critical care in a lower resource setting such as Tanzania.
- The search strategy broadly and comprehensively included studies on critical care and costs of inpatient care in Tanzania thereby covering majority of published studies on the subject.
- This study has a fully developed population, intervention, comparison and outcome framework.
- The study included only articles published in English.
- There was a chance that there is limited relevant data on Tanzania we included diseases likely to require critical care (eg, malaria and pneumonia). The inclusion of some of these diseases may have introduced some selection bias.

For peer review only

Background

Critical care is an essential element of healthcare whose importance has been highlighted globally during the COVID-19 pandemic with countries experiencing overwhelming numbers of critically ill patients [1–3]. As a result, the care of critical illness has become an urgent point of focus in global health policy.

Critical care entails the care given to a patient in need of enhanced monitoring, treatment and attention, due to a life-threatening illness or injury[4]. There have been great advances in the provision of critical care for example invasive and non-invasive monitoring techniques, mechanical ventilation (MV), and renal replacement therapy, among others, resulting in reduced mortality rates in patients with critical illness, especially in high income countries[5].

However, such critical care is provided in sophisticated intensive care units using equipment which are costly to procure and maintain[6]. This makes it difficult for low resource settings to acquire such equipment to meet the needs of their populations and has led to recommendations for the development of more affordable critical care models that can be provided in any hospital settings and to all critically ill patients [1,7,8]. Moreover, most critically ill patients do not need critical care at ICU-level[9] and large unmet needs of even basic lifesaving critical care have been found in LMICs[10–12]. With various national and international stakeholders calling for critical care scale-up[13], it is necessary to understand the resources needed in the provision of critical care and their respective costs. Unfortunately,

For peer review only - http://bmjopen.bmj.com/site/about/guidelines.xhtml

knowledge on the current availability of resources and the costs of critical care remains sparse[1,14,15]. There is no systematic review that examines the available resources, utilisation and costs of critical care generally[16] nor in low and middle income country settings.

To inform the planning and scale up of critical care provision in a low-income country setting, this study takes a case study approach to review available knowledge on the current resource availability, utilisation and cost in the provision of critical care in Tanzania[16].

Methods

The systematic review followed the Preferred Reporting Items for Systematic Reviews and Meta-Analyses (PRISMA) guidelines[17] and the protocol was registered with PROSPERO; registration number: CRD42020221923[16]. The full methodological protocol used in this review is available elsewhere[16].

Search strategy and selection process

Following PRISMA guidelines, 3 electronic databases (i.e. Medline, Embase and Global Health) were systematically searched for articles published since 2010 [18]. A ten-year time frame was considered to ensure that findings reflect the present-day resource utilization

For peer review only - http://bmjopen.bmj.com/site/about/guidelines.xhtml

paradigm as resources used and their costs vary as technology and clinical guidelines evolve. Bibliographies of included articles were reviewed to find relevant articles that fulfil the inclusion criteria but had not been identified. Google and Google Scholar were also used to search for published articles that may not have been indexed within the databases. As internet search engines typically return several thousand results, the searches were restricted to the first fifty hits and links to potentially relevant material were accessed[19].

Table 1PICO (Population, Intervention, Comparator, Outcome) Framework used to inform the search strategy

**Element**	**Description**
Population	Any patient in need of critical care
Intervention	Inpatient services that could form part of critical care provision
Comparison	No critical care
Outcomes	Critical care services available<br><br>Cost of the different inputs to critical care provision per patient<br><br>Quantity and type of resources used in provision of critical care per patient

The PICO framework (table 1) describes major components used to identify key words. Other concepts and search terms are listed in the supplementary material. Key words included critical care, critical illness, resource use and setting. Some diseases where also added to the research string (for example neonatal disorders, HIV, Malaria and TB[20]), on the premise that these are conditions that have a high

For peer review only - http://bmjopen.bmj.com/site/about/guidelines.xhtml

risk of critical illness. See supplementary 1 for detailed key words. Rayyan QCRI software was used to identify and remove duplicates[21] and screen articles by title and abstract through a double-review process.

Eligibility criteria

A study was considered eligible if it was published in English and reported on the provision of critical care[22], services offered under critical care, resources and or costs incurred by the health system or health providers in providing critical care in Tanzania. A detailed inclusion/ exclusion criterion can be found in supplementary 2.

Data extraction and synthesis

A bespoke data extraction form was developed and used to record extracted data. The extracted data included author, year of publication, context (location, setting- urban or rural, type of facility, level of facility), critical care services offered, critical care equipment available, costing perspective, costing year, currency used, type of provider, payer, source of cost data, costing time frame, direct medical costs, resources used.

A narrative synthesis of extracted data was conducted to report an overview of study characteristics of the eligible studies. We described the characteristics of critical care provision as identified in the literature and resources available for the provision of critical care in Tanzania. In addition, quantitative and qualitative syntheses were performed for cost and resource use data. Costs and resources used

For peer review only - http://bmjopen.bmj.com/site/about/guidelines.xhtml

For peer review only

were categorized into groups based on a standard input-wise categorisation, for example, human resources, diagnostics, services, consumables and medication. Costs were first standardized by adjusting for inflation using the GDP deflators for Tanzania[23] and reported in USD and TZS of 2019 value.

Risk of bias in individual studies

The quality of the included articles was assessed by two in dependent researchers using an adaptation of the Reference Case for Estimating the Costs of Global Health Services and Interventions[24], which provides a framework for quality assessment and data extraction. The framework is used to identify likely sources of bias. Furthermore, methodological quality of studies that reported costs were further assessed by an appraisal checklist by Adawiyah et al[25]. Any discrepancies were addressed by a joint re-evaluation of the article among 3 authors.

Patient and Public Involvement

This systematic review did not involve any patient nor public in the design, data extraction and analysis. However, the findings of this systematic review will be made available to the public through an open access manuscript.

[Insert figure 1: PRISMA Chart]

For peer review only - http://bmjopen.bmj.com/site/about/guidelines.xhtml

Results

Study characteristics

Thirty-one studies fulfilled the inclusion criteria (figure 1), of which 14 reported on the costs associated with critical care in Tanzania (please see supplementary 3: table of study characteristics (N=31)). The disease areas covered by eligible studies included; oncology (n=1), trauma (n=4), obstetrics (n=3), neonatal/child care (n=2), HIV (n=1), renal failure (n=1), TB (n=1), malaria (n=3), stroke (n=1) and typhoid (n=1), antimicrobial resistance (n=1) surgery (n=2) respiratory illness (n=1), blood transfusion (n=1), no disease or area specific (n=8). The majority of studies were cross sectional in design (n=20) and qualitatively reported on the location of critical care and critical care services available.

Most studies were conducted in a specific region: the Coast Zone (n = 7), Lake Zone (n = 3), Northern Highland Zone (n = 3) and Southern Highland Zone (n = 3) and Central Zone (n = 1). A zone is a geographical area made up of health regions which are made up of districts. Seven studies were nationally representative (n = 7); two studies didn't specify a given region. Three studies were carried out in rural settings while the rest were urban or mixed settings. Nine studies specified that they were conducted in in public (government owned) facilities and six specified non-governmental organisations including faith/missionary based hospitals.

For peer review only - http://bmjopen.bmj.com/site/about/guidelines.xhtml

Critical care provision in Tanzania

The structure of the health system in Tanzania comprises six levels[26] starting with the village health post (lowest level), dispensaries, health centres, district hospitals, regional hospitals and tertiary referral hospitals (the highest level) [26–39]. Critical care is usually offered at the hospital level only, that is the district hospital, regional hospitals and referral hospitals [26,27,37–40,28–31,33–36]. In addition, Nicks et al state that district hospitals offer basic forms of critical care while referral hospitals offer critical care in ICUs[26]. Thirteen of the fifteen studies that reported on critical care provision were carried out in urban settings. Two of the fifteen studies were carried out in both rural and urban settings; no study was exclusively carried out in a rural setting. Based on the literature, we further categorise critical care into "ICU delivered critical care" (otherwise known as ICU based critical care) and "non-ICU based critical care".

Seven studies reported on ICU delivered critical care offered in tertiary referral hospitals in Tanzania[28,29,35,36,38,39] including regional hospitals. For example, Murthy et al state that St. Francis Hospital in Ifakara region had 10 ICU beds and Sekou Toure Regional Referral Hospital in Mwanza had 8 ICU beds[33]. ICU delivered critical care services included intubation and ventilation. For example, one study found that 54.2% of all ICU patients were intubated or ventilated[38]. In addition, two studies reported surgical interventions that were conducted on patients cared for in ICU included wound debridement, treatment of fractures, craniotomy, underwater seal

For peer review only - http://bmjopen.bmj.com/site/about/guidelines.xhtml

drainage, tracheostomy, eye surgery and Thoracotomy[38,41]. The district hospitals, for which studies were available did not provide ICU delivered critical care [34].

Critical care outside the ICU was reported to be delivered either in operating theatres or in emergency units and general wards. For example, operating theatres at Kilimanjaro Christian Medical Centre (KCMC) offered critical care for cases like trauma and sepsis[42]. Theatres in lower-level hospitals like district hospitals often offer initial critical care before referring the patient to higher level hospitals that can offer ICU based critical care. Although district hospitals and some regional hospitals were reported not to have ICUs, these facilities were reported to offer critical care in the general wards[34]. Some hospitals without ICUs designated areas within general wards to cater for the critically ill patients[34]. Furthermore, Staton et al found that some critical care services including intubation were performed in the emergency units[39].

Availability of resources required for critical care

The majority of eligible studies reported on the known resources used for critical care (n=21). However, many studies did not report the extent to which the resources were readily available at a given hospital (n=18). Three studies reported on availability and use of critical care resources and indicate that their availability varies between hospitals and is sometimes unreliable or inconsistent (supplementary 4). Two studies find that there is a scarcity of trained staff in critical care[34,37]. Further, the availability of critical care

For peer review only - http://bmjopen.bmj.com/site/about/guidelines.xhtml

equipment was heterogenous with scarcity in the areas of mask and tubing, pulse oximetry, oxygen cylinders, adult and paediatric oropharyngeal airway and PPE eye protection highlighted. In addition, a greater scarcity in paediatric than adult critical care equipment was identified [34,37].

Resource use and cost of resources associated with provision of critical care

Of the 31 studies, 14 reported on the resource use and critical care costs and were appraised for quality using the Adawiyah et al adapted appraisal checklist[25] (Supplementary 5). All 14 studies were found to have clear research questions, mentioned the specific costing perspective, included relevant inputs, clearly stated methods for quantifying resources and reported costs. Four studies did not specify the time horizon while four studies partially addressed it. The majority of the studies (n=8) carried out some form of sensitivity analysis. All studies that collected primary data stated sample size but did not mention how it was determined and whether it was sufficient.

Types of costs reported and sources of data

Table 2: Types of costs reported and sources of data

Study	Economic/financial cost/both	Real world /guideline cost	Full/incremental cost	Top down/ bottom up estimations/both	Source of cost data
Githang'a et 2020	Financial	Real world costs	Full costs	Both	Hospital data
Mengistu et al 2019	Financial	Real world costs	Full costs	Top-down estimation	Programme, hospital and health center data
Wilson et al 2020	Financial	Real world costs	Full costs	Bottom-up estimation	Staff Survey (N=59)
Kimaro et al 2017	Economic costs	Real world costs	Full costs	Bottom-up estimation	Patient survey, diaries (N=870)

For peer review only - http://bmjopen.bmj.com/site/about/guidelines.xhtml

Shayo et al 2017	Economic costs	Real world costs	Full costs	Bottom-up estimation	Records, government standards and market price survey
Phillips et al 2015	Economic costs	Real world costs	Full costs	Top down estimation	Local program records
Penno et al 2015	Economic costs	Real world costs	Full costs	Bottom-up estimation	Laboratory records
Kabadi et al 2013	Economic costs	Real world costs	Full costs	Bottom-up estimation	Survey (N=16) and project data
Sicuri et al 2011	Economic costs	Real world costs	Full costs	Bottom-up estimation	Patient Survey (N=150)
Guerriero et al 2010	Economic costs	Real world costs	Full costs	N/A	Literature
Baynes et al 2019	Financial costs	Real world costs	Full costs	Bottom-up estimation	Health facility Records (N=31) and key informant interviews (N=124)
Riewpaiboon et al 2014	Economic costs	Real world costs	Full costs	Bottom-up estimation	Record review and patient survey (N=17)
Sumona et al 2016	Financial costs	Real world costs	Full costs	Bottom-up estimation	Health facility Survey (N=386)
Chris et al 2013	Financial costs	Real world costs	Full costs	Top-down estimation	Facility Survey (N=155)

Published costs of critical care were reported as economic costs (n = 8) or financial costs (n = 6), using a mix of bottom up (n = 9) and top down (n = 5) approaches. Sources of the costs were local data collected from hospital records, surveys and price lists. Surveys (including key informant interviews) were a common method of data collection (n = 9). Three studies used patient level data on resource use to inform costs (Kimaro[43]; Sicuri[44]; Riewpaiboon[45]). Hospital or laboratory records were used in 6 of the 14 studies. Some studies used both survey and records review as the source of data (n = 6). Two studies used international literature as a source of data (n =2). Unit costs reported included cost per program, cost per patient, cost per visit, cost per patient day, cost per episode, cost per bed day, inpatient cost, total annual cost, cost per case (see table 2).

Cost of services offered to patients

Table 3: Cost of services offered to patients

**Study**	**Description**	**Unit cost of output**	**Currency year**	**Unit cost Price in 2019 (USD)**	**Unit cost Price in 2019 (TZS)**
Mengistu et al 2019	Clinical care for emergency obstetric case	per case	2013	143.85	329,166.
Mengistu et al 2019	Average cost per surgical patient	per patient	2013	285.08	652,333.26

For peer review only
For peer review only - http://bmjopen.bmj.com/site/about/guidelines.xhtml

Mengistu et al 2019	Average cost per patient undergoing caesarean	Per patient	2013	471.28	1,078,396.41
Riewpaiboon et al 2014	Inpatient hospital cost	Per patient per day	2010	91.78	107.49
Sicuri et al 2011	Episode of Malaria hospitalisation and Severe anaemia	per episode	2009	57.12	130,704.61
Sicuri et al 2011	Episode of Cerebral malaria	per episode	2009	22.67	51,864.33
Sicuri et al 2011	Episode of Cerebral malaria and neurological sequela	per episode	2009	47.58	108,879.84
Kabadi et al 2013	Hospitalisation of stroke patient (Inpatient, Drugs, Registration)- Rural area	per patient	2005-2006	12.76	29,200.18
Kabadi et al 2013	Hospitalisation of stroke patient (Inpatient, Drugs, Registration)- Urban area	per patient	2005-2006	31.17	71,317.89
Kabadi et al 2013	Physiotherapy for stroke- urban area	per patient	2005-2006	44.88	102,697.20
Kabadi et al 2013	Hospitalisation of stroke patient (Inpatient, Drugs, Registration)- average	per patient	2005-2006	18.51	42,361.25
Kabadi et al 2013	Physiotherapy for stroke- average	per patient	2005-2006	14.03	32,093.43
James, Bura & Ensor 2013	Health centre: overall	Inpatient day	2011/12	18.03	41,261.22
James, Bura & Ensor 2013	Health centre: public	Inpatient day	2011/12	18.98	43,432.74
James, Bura & Ensor 2013	health centre: NGO	Inpatient day	2011/12	17.29	39,564.68
James, Bura & Ensor 2013	Hospital: all	Inpatient day	2011/12	25.95	59,387.93
James, Bura & Ensor 2013	Hospital: public	Inpatient day	2011/12	18.54	42,422.55
James, Bura & Ensor 2013	Hospital: NGO	Inpatient day	2011/12	15.76	36,062.52
James, Bura & Ensor 2013	Hospital: private	Inpatient day	2011/12	68.05	155,721.31
James, Bura & Ensor 2013	Hospital regional	Inpatient day	2011/12	17.33	39,653.32
James, Bura & Ensor 2013	Hospital - ARI-pneumonia	per case treated	2011/12	104.33	238,736.91
James, Bura & Ensor 2013	Hospital - Hypertension	per case treated	2011/12	253.98	581,166.07
James, Bura & Ensor 2013	Hospital - Myocardial infarction	per case treated	2011/12	268.57	614,533.21

237

238 Although many studies reported costs within differing disease specific (e.g. malaria and anaemia) or general hospital services (e.g.
239 inpatient and outpatient care), no study identified the cost of elements that are specific for the treatment of critical illness. The highest
240 reported average cost per patient was for a patient undergoing caesarean section (USD 471.28)[46] . The lowest reported cost per patient
241 was for hospitalisation for stroke in a rural area (USD 12.76)[47]. A nationally representative study of general hospital services including
242 critical care found that unit costs vary by provider ownership (public, NGO and private) and level of health facility[48] (regional vs

For peer review only - http://bmjopen.bmj.com/site/about/guidelines.xhtml

243 primary care). Services provided at private hospitals were more expensive than the same services received at public hospitals. NGO
244 hospitals were less expensive than public hospitals.

245 *Cost of Equipment, consumables and medication*

246 **Table 4: Cost of Equipment, consumables and medication**

Study	Description	Unit cost of output	Currency year	Unit cost Price in 2019 (USD)	Unit cost Price in 2019 (TZS)
**Equipment**					
Chaudhury et al 2016	Mannequin	per piece	2014	76.03	173,970.65
Chaudhury et al 2016	Bag-mask device	per piece	2014	16.29	37,279.43
Chaudhury et al 2016	Penguin sucke	per piece	2014	3.26	7,455.89
Mengistu et al 2019	Equipment needed for emergency obstetric care	per patient	2013	90.02	205,994.65
**Consumables**					
Mengistu et al 2019	Other supplies for emergency obstetric care	per patient	2013	28.68	65,635.80
Mengistu et al 2019	Blood supplies	per patient	2013	0.71	1,620.01
Guerriero et al 2010	Consumables needed in the administration of tranexamic acid (10ml syringe,100ml bag of saline, large gauge needle)	Per dose	2007	2.00	4,576.41
**Medication**					
Analgesics and sedatives					
Riewpaiboon et al 2014	Paracetamol 120 mg/5 mL in 60-mL bottle	per bottle	2010	0.33	750.38
Riewpaiboon et al 2014	Paracetamol 500 mg tablet	Per tablet	2010	0.01	26.80
Antifungal and antibacterial drugs					
Riewpaiboon et al 2014	Ampicillin 500 mg vial injection	Per piece	2010	0.21	482.39
Riewpaiboon et al 2014	Ceftriaxone 250 mg vial injection	Per piece	2010	0.46	1,045.17
Riewpaiboon et al 2014	Ciprofloxacin 250 mg capsule	Per Capsule	2010	0.01	26.80
Riewpaiboon et al 2014	Ciprofloxacin 500 mg capsule	Per capsule	2010	0.05	107.20
Penno et al 2015	Antimicrobial treatment using generic drugs for sepsis management	per case	2011	15.17	34,703.66
Penno et al 2015	Evidence-based antimicrobials	Per case	2011	30.36	69,459.83
Kimaro et al 2017	Cotrimoxazole per daily dose	Per daily dose	2012	0.02	51.51
Others like anti-cancers					
Githang'a et al 2020	50 mg of 6-mercaptopurine (6MP)	Unclear	2013	0.39	885.94
Mengistu et al 2019	Drugs for emergency obstetric care	per patient	2013	6.90	15,795.12

For peer review only - http://bmjopen.bmj.com/site/about/guidelines.xhtml

Riewpaiboon et al 2014	Glucose (5%) 500 mL	Per unit	2010	0.50	1,152.37
Guerriero et al 2010	Tranexamic acid	Per dose	2007	9.74	22,279.66

When looking at cost categories, 2 studies reported costs of equipment, 2 studies reported costs of consumables and 6 studies reported medication cost. Within studies, we found per item cost for equipment (4 items), consumables (3 items) and medication (13 items). The cost of a mannequin used in training was the most expensive piece of equipment reported (USD 76.03)[49]. In the medication category, tranexamic acid was the most expensive per dose (USD 9.74)[50], whereas antimicrobials were the most expensive on a per case basis (USD 30.36)[51].

Cost of diagnostics

While diagnostics are not part of critical care provision, they form part of the overall patient care and can inform future economic evaluations. Six studies reported on the cost of diagnostics, for example, FBC, serum cryptococcal meningitis test, sputum culture and imaging.

Costs of human resources

Three studies reported human resource costs (see supplementary 6). No human resource costs were identified as specifically for ICU or non-ICU critical care. Reporting of human resource cost was heterogenous. One study provided monthly salaries for all personnel with monthly salaries ranging from USD 2721.79 for medical specialists USD 313.34 for nurses[52]. Another study reported total cost of personnel as a line item. The third study reported unit costs (i.e. cost per patient) treatment for all obstetric complications broken

For peer review only - http://bmjopen.bmj.com/site/about/guidelines.xhtml

down by staff category. This study found that the most important cost was for an enrolled nurse or midwife (USD 43.62 per patient) and that the lowest unit personnel cost was for an anaesthesiologist (less than 0.01USD per patient)[53].

Discussion

Our review found limited evidence on resource use, availability and costs of critical care in Tanzania. Of the eligible evidence, we found high variability in the geographical distribution and availability of resources needed for both ICU and non-ICU critical care provision. We found that ICU critical care was provided at the tertiary level in urban areas and no published provision in rural areas, whereas, non-ICU critical care was evident in both rural and urban areas. Within hospitals, we find that availability of key critical care resources (e.g. pulse oximeters, oxygen cylinders, mask and tubing) can be limited but the use of these resources is not documented. We, finally, find no reported estimates on the unit costs of critical care; and that the generation and reporting of cost data is not standardised thereby limiting synthesis or aggregation.

Availability of critical care in Tanzania

For peer review only - http://bmjopen.bmj.com/site/about/guidelines.xhtml

The majority of ICUs identified in our systematic review were located in tertiary referral hospitals and private hospitals located in urban areas (Dar es Salaam, Moshi, Mwanza, Mbeya city). This is of particular concern in Tanzania where the majority of the population (64.77) lives in rural areas[54]. Although this inequity in service distribution is not unique to Tanzania as previous studies in LMICs found all hospitals with ICUs were located in cities [33] , it is further exacerbated by a lack of critical care guidelines that cater to rural areas[55]. Such geographical inequity in ICU critical care could be detrimental and of particular concern during a critical care crisis (e.g. COVID-19)[1].

However, critical care delivery is heterogenous and can be provided in advanced or basic forms, depending on resource availability. Critical care can involve high tech and high-cost equipment that requires highly trained staff (e.g. mechanical ventilation) and is more often found in the ICU. Investing in this level of critical care is therefore an expensive policy decision and may explain the lack of ICU critical care availability in rural areas in Tanzania. On the other hand, investing in adequately resourced critical care outside of ICUs can potentially save lives and reduce the case load that requires ICU care [56]. We found that non-ICU critical care was reported to be given in wards and theatres[39] across rural and urban areas.

A key finding of the literature review was that neither the level of care provided, complexity, resource intensity or quality of provision was reported. We were therefore unable to characterise critical care in Tanzania other than by where it is delivered i.e. ICU vs non-ICU based critical care. This is of concern as care for the critically ill takes many forms and is not necessarily synonymous with ICU

For peer review only - http://bmjopen.bmj.com/site/about/guidelines.xhtml

care. Critical care, at least in its more basic forms, should be readily available to all patients in need in any hospital. Characterising the minimum needs for the care of a critically ill patient will facilitate our understanding of what elements of critical care are currently available as well as recommendations for the progressive improvement of critical care in all facilities. Previous research suggests that basic critical care, which effectively identifies and treats all critically ill patients, should be available and provided in all settings within a hospital (e.g. in wards) across rural and urban settings [57,58]. Recently, some of this paper's authors have proposed the concept of Essential Emergency and Critical Care (EECC) across LMICs. EECC is pragmatic and low-cost care of critical illness that does not need to be reliant on sophisticated technology, and have a primary focus on simple, effective actions that have large potential impact on the population[56] and the components of have been specified in a large global consensus[8].

Given the lack of availability and access of critical care across Tanzania, such novel approaches may provide a building block to developing a fully resourced, equitable critical care system[59]. However, decision makers require further evidence on the optimal design, costs, budget impact and cost-effectiveness of such critical care interventions in rural and urban systems.

Complexity of resources within critical care

At the hospital level, we found that many studies identified resources that were used in the provision of services that included a critical care component but a few studies went further to report on the availability of the resources at the facility (e.g. equipment, consumables

For peer review only

and human resources). For example, while oxygen is one of the essential resources required in the provision of critical care, many hospitals still lack basic equipment for its storage and delivery[60]. Our findings show that even when oxygen is available, the equipment and consumables required to facilitate provision of oxygen therapy can be missing[39,61]. This leaves a considerable proportion of critically ill patients in need of medical oxygen without the necessary treatment, incurring unnecessary deaths and disability.

Many reasons exist as to why availability and supply of oxygen is limited in settings such as Tanzania. For example, supply and scaling of medical oxygen is a complex process and is often controlled though monopoly suppliers. Regulation and high investment costs create barriers for new firms to enter the market and easily scale up supply. In Tanzania, medical oxygen supply is controlled centrally by a quasi-governmental monopoly organisation[62]. On the demand side, there is limited funding for medical oxygen with procurement decentralised to individual hospitals with constraint budgets[60]. Alternatives, such as oxygen concentrators and hospital plants for the local production of oxygen, are affected by the need for a constant supply of electricity, compounded by challenges with maintaining equipment. In Tanzania, approx. 50% of hospitals are without a constant oxygen supply[37] and there is limited local capacity to maintain and repair oxygen concentrators like Tanzania.

In addition to oxygen equipment, we found variability in availability of PPE and human resources across studies. While some personal protective equipment (PPE), especially gloves, were available in most hospitals, others, such as eye protection, remained unavailable,

For peer review only - http://bmjopen.bmj.com/site/about/guidelines.xhtml

which is in line with previous research[63]. In terms of human resources, evidence suggests that only 20% of hospitals have at least one health worker trained in adult critical care which includes administering complex oxygen related equipment [34,55,57]. Technical assistance, training of health workers, and improved processes for non ICU based care, development of context-specific critical care guidelines, building capacity in equipment maintenance and biomedical engineering in facilities, and support to medical oxygen infrastructure development should be prioritised in applications to multilaterals and technical assistance agencies [34,64,65].

Costs of critical care

Costing of critical care is vital in eliciting potential budget constraints or impacts, assessing cost-effectiveness, providing insight into the economic burden of disease and can be useful for understanding the resources incurred by health systems, other payers, and patients. We find that no study reported or calculated an average cost or cost per patient day for the provision of critical services. On the other hand, many studies reported individual costs of resources associated with provision of critical care and it was possible to identify the cost of specific resources needed in critical care provision (i.e. human resource cost, services, medication, and other consumables). Such limited evidence could be due to no formal or standardised definition of critical care existing thereby reducing specificity when costing critical care provisioning. Another possibility may be that patients who require critical care are heterogenous. The underlying demographics, epidemiology, risk factors, patient pathway and interventions administered are also heterogenous thereby making aggregation and estimates of average cost challenging. This is further confirmed by the data extracted in this review

For peer review only - http://bmjopen.bmj.com/site/about/guidelines.xhtml

which showed that reporting of costs within eligible studies were inconsistent thereby making comparisons or synthesising information across studies difficult. There is an urgent need to understand the resources required and their cost implications to inform the planning of improved critical care, globally.

Limitations

There are a number of inherent limitations with this systematic review. Firstly, we tailored our search strategy to account for the lack of standardised or formal definitions for critical illness, critical care provision and ICUs [57] by including specific terms for conditions likely to lead to critical illness and identified in consultation with clinicians on the authorship team. Here, we may have introduced a personal bias by focussing on specific conditions and excluding others. Secondly, cost and resource use in an African context are traditionally poorly documented and can fluctuate due to market dynamics and resource availability. While this systematic review provides key insights into peer reviewed published information on the resource use, availability and costs of critical care in Tanzania, researchers must update a previous nationally representative facility survey conducted in 2012 extending it to include an understanding of the costs of critical care provision that includes patient level data on resource use. Finally, we opted to only include articles in English but believe the extent and effects of language bias are diminished recently because of the shift towards publication of studies in English[66].

For peer review only - http://bmjopen.bmj.com/site/about/guidelines.xhtml

Conclusion

Critical care is essential in the prevention of mortality among critically ill patients, yet limited evidence exists on the costs and cost-effectiveness of scaling up critical care thereby hindering informed planning for investments in key life-saving interventions. We further find that ICU based critical care provision is currently only provided in urban areas in Tanzania and there is an urgent need to assess the quality of non-ICU based critical care implemented to address the gap in critical care provision in rural and urban areas. Evidence on resources used and potential costs in critical care is limited, and, along with a specific focus on oxygen supply and delivery which is a key resource in critical care, requires further research. Improving the evidence base on the costs, utilisation, cost-effectiveness and equity impacts of different strategies for critical care services across rural and urban areas is needed to help prevent unnecessary deaths caused by critical illness in Tanzania.

References

1. Baker T, Otto Schell C, Brun Petersen D, et al. Essential care of critical illness must not be forgotten in the COVID-19 pandemic. *Lancet*. 2020;395:1253-1254. doi:10.1016/S0140-6736(20)30793-5

For peer review only - http://bmjopen.bmj.com/site/about/guidelines.xhtml

2. Barasa E, Kairu A, Nganga W, et al. Examining Unit Costs for COVID-19 Case Management in Kenya. *medRxiv*. Published online October 13, 2020:2020.10.08.20209684. doi:10.1101/2020.10.08.20209684

3. Sen-Crowe B, Sutherland M, McKenney M, Elkbuli A. A Closer Look Into Global Hospital Beds Capacity and Resource Shortages During the COVID-19 Pandemic. *J Surg Res*. 2021;260:56. doi:10.1016/J.JSS.2020.11.062

4. National Institute for Health and Care Excellence (NICE). What "critical care" means | Information for the public | Rehabilitation after critical illness in adults | Guidance | NICE. Published March 25, 2009. Accessed November 1, 2020. https://www.nice.org.uk/guidance/cg83/ifp/chapter/What-critical-care-means

5. Zimmerman JE, Kramer AA, Knaus WA. Changes in hospital mortality for United States intensive care unit admissions from 1988 to 2012. *Crit Care*. 2013;17(2):R81. doi:10.1186/cc12695

6. Turner HC, Van Hao N, Yacoub S, et al. Achieving affordable critical care in low-income and middle-income countries Commentary Handling editor Seye Abimbola. *BMJ Glob Heal*. 2019;4:1675. doi:10.1136/bmjgh-2019-001675

7. Schell CO, Gerdin Wärnberg M, Hvarfner A, et al. The global need for essential emergency and critical care. *Crit Care*. 2018;22(1):1-5. doi:10.1186/s13054-018-2219-2

8. Schell CO, Khalid K, Wharton-Smith A, et al. Essential Emergency and Critical Care: a consensus among global clinical experts. *BMJ Glob Heal*. 2021;6(9):e006585. doi:10.1136/BMJGH-2021-006585

For peer review only

For peer review only - http://bmjopen.bmj.com/site/about/guidelines.xhtml

9. Kayambankadzanja RK, Schell CO, Namboya F, et al. The prevalence and outcomes of sepsis in adult patients in two hospitals in Malawi. *Am J Trop Med Hyg*. 2020;102(4):896-901. doi:10.4269/ajtmh.19-0320

10. Kayambankadzanja RK, Schell CO, Mbingwani I, Mndolo SK, Castegren M, Baker T. Unmet need of essential treatments for critical illness in Malawi. Chen T-H, ed. *PLoS One*. 2021;16(9):e0256361. doi:10.1371/JOURNAL.PONE.0256361

11. King C, Banda M, Bar-Zeev N, et al. Care-seeking patterns amongst suspected paediatric pneumonia deaths in rural Malawi. *Gates Open Res 2021 4178*. 2021;4:178. doi:10.12688/gatesopenres.13208.2

12. Hillary C, Tom P, P. K, A.M. M, G. S, D. M. A survey of emergency and surgical services in the United Republic of Tanzania. *Acad Emerg Med*. 2011;18(5 SUPPL. 1):S242. doi:http://dx.doi.org/10.1111/j.1553-2712.2011.01073.x

13. World Health Organisation. *Strengthening the Health Systems Response to COVID-19: Creating Surge Capacity for Acute and Intensive Care* .; 2020. Accessed September 21, 2021. http://apps.who.int/bookorders.

14. World Bank. World Bank facilitates immediate support and resources for the COVID-19 (Coronavirus) emergency in Bolivia. Published April 2, 2020. Accessed December 16, 2020. https://www.worldbank.org/en/news/press-release/2020/04/02/el-banco-mundial-facilita-apoyo-y-recursos-inmediatos-para-atender-la-emergencia-por-el-covid-19-coronavirus-en-bolivia

15. The World Bank. *The World Bank Annual Report 2020: Supporting Countries in Unprecedented Time*.; 2020. Accessed December 16, 2020. https://www.worldbank.org/en/about/annual-report/covid-response

For peer review only

16. Kazibwe J, Shah HA, Kuwawenaruwa A, et al. Resource availability, utilisation and cost in the provision of critical care in Tanzania: a protocol for a systematic review. *BMJ Open*. 2021;11(8):e050881. doi:10.1136/BMJOPEN-2021-050881

17. Page MJ, McKenzie JE, Bossuyt PM, et al. The PRISMA 2020 statement: an updated guideline for reporting systematic reviews. *BMJ*. 2021;372. doi:10.1136/BMJ.N71

18. Moher D, Liberati A, Tetzlaff J, et al. Preferred reporting items for systematic reviews and meta-analyses: The PRISMA statement (Chinese edition). *J Chinese Integr Med*. 2009;7(9):889-896. doi:10.3736/jcim20090918

19. Savilaakso S, Garcia C, Garcia-Ulloa J, et al. Systematic review of effects on biodiversity from oil palm production. *Environ Evid*. 2014;3(1):4. doi:10.1186/2047-2382-3-4

20. Abbafati C, Machado DB, Cislaghi B, et al. Global burden of 369 diseases and injuries in 204 countries and territories, 1990–2019: a systematic analysis for the Global Burden of Disease Study 2019. *Lancet*. 2020;396(10258):1204-1222. doi:10.1016/S0140-6736(20)30925-9

21. Ouzzani M, Hammady H, Fedorowicz Z, Elmagarmid A. Rayyan-a web and mobile app for systematic reviews. *Syst Rev*. 2016;5(1). doi:10.1186/s13643-016-0384-4

22. Kazibwe J, Shah HA, Kuwawenaruwa A, et al. Resource availability, utilization and cost in the provision of critical care in Tanzania: A protocol for a systematic review. *BMJ Open*. Published online 2021.

For peer review only - http://bmjopen.bmj.com/site/about/guidelines.xhtml

23. World bank. GDP deflator (base year varies by country) - Tanzania . Accessed June 2, 2021. https://data.worldbank.org/indicator/NY.GDP.DEFL.ZS?locations=TZ

24. Vassall A, Sweeney S, Kahn JG, et al. *Reference Case for Estimating the Costs of Global Health Services and Interventions*.

25. Adawiyah R al, Saweri OPM, Boettiger DC, et al. The costs of scaling up HIV and syphilis testing in low- and middle-income countries: a systematic review. *Health Policy Plan*. 2021;2021:1-16. doi:10.1093/heapol/czab030

26. Nicks BA, Sawe HR, Juma AM, Reynolds TA. The state of emergency medicine in the United Republic of Tanzania. *African J Emerg Med*. 2012;2(3):97-102. doi:10.1016/j.afjem.2012.06.002

27. Sylvanus E, Sawe HR, Muhanuzi B, et al. Profile and outcome of patients with emergency complications of renal failure presenting to an urban emergency department of a tertiary hospital in Tanzania. *BMC Emerg Med*. 2019;19(1):11. doi:http://dx.doi.org/10.1186/s12873-019-0229-2

28. Engdahl Mtango S, Lugazia E, Baker U, Johansson Y, Baker T. Referral and admission to intensive care: A qualitative study of doctors' practices in a Tanzanian university hospital. Brenner S, ed. *PLoS One*. 2019;14(10):e0224355. doi:10.1371/journal.pone.0224355

29. Mkoka DA, Goicolea I, Kiwara A, Mwangu M, Hurtig AK. Availability of drugs and medical supplies for emergency obstetric care: Experience of health facility managers in a rural District of Tanzania. *BMC Pregnancy Childbirth*. 2014;14(1):1-10.

For peer review only

doi:10.1186/1471-2393-14-108

30. Papali A, Adhikari NKJ, Diaz J V., et al. Infrastructure and organization of adult intensive care units in resource-limited settings. In: *Sepsis Management in Resource-Limited Settings*. Springer International Publishing; 2019:31-68. doi:10.1007/978-3-030-03143-5_3

31. Mwiga F, Monaghan M. Development of a difficulty airway trolley (DAT) at a tertiary referral hospital in northern Tanzania. *Trends Anaesth Crit Care*. 2020;30:e107-e108. doi:http://dx.doi.org/10.1016/j.tacc.2019.12.265

32. Sawe HR, Reynolds TA, Mfinanga JA, et al. The clinical presentation, utilization, and outcome of individuals with sickle cell anaemia presenting to urban emergency department of a tertiary hospital in Tanzania. *BMC Hematol*. 2018;18(1):25. doi:http://dx.doi.org/10.1186/s12878-018-0122-3

33. Murthy S, Leligdowicz A, Adhikari NKJ. Intensive care unit capacity in low-income countries: A systematic review. *PLoS One*. 2015;10(1):116949. doi:http://dx.doi.org/10.1371/journal.pone.0116949

34. Baker T, Lugazia E, Eriksen J, Mwafongo V, Irestedt L, Konrad D. Emergency and critical care services in Tanzania: A survey of ten hospitals. *BMC Health Serv Res*. 2013;13(1):1-9. doi:10.1186/1472-6963-13-140

35. Rajaguru PP, Jusabani MA, Massawe H, Temu R, Sheth NP. Understanding surgical care delivery in Sub-Saharan Africa: a cross-sectional analysis of surgical volume, operations, and financing at a tertiary referral hospital in rural Tanzania. *Glob Heal*

For peer review only - http://bmjopen.bmj.com/site/about/guidelines.xhtml

Res policy. 2019;4:30. doi:https://dx.doi.org/10.1186/s41256-019-0122-2

36. Reynolds TA, Mfinanga JA, Sawe HR, Runyon MR, Mwafongo V. Emergency care capacity in Africa: a clinical and educational initiative in Tanzania. *J Public Health Policy*. 2012;33(Supplement 1):S126-S137. doi:http://dx.doi.org/10.1057/jphp.2012.41

37. Penoyar T, Cohen H, Kibatala P, et al. Emergency and surgery services of primary hospitals in the United Republic of Tanzania. *BMJ Open*. 2012;2(1):e000369. doi:10.1136/bmjopen-2011-000369

38. Chalya PL, Gilyoma JM, Dass RM, et al. Trauma admissions to the Intensive care unit at a reference hospital in Northwestern Tanzania. *Scand J Trauma Resusc Emerg Med*. 2011;19:61. doi:10.1186/1757-7241-19-61

39. Staton CA, Msilanga D, Kiwango G, et al. A prospective registry evaluating the epidemiology and clinical care of traumatic brain injury patients presenting to a regional referral hospital in Moshi, Tanzania: challenges and the way forward. *Int J Inj Contr Saf Promot*. 2017;24(1):69-77. doi:10.1080/17457300.2015.1061562

40. Sawe HR, Reynolds TA, Mfinanga JA, et al. The clinical presentation, resource utilization, and outcomes of patients with sickle cell disease presenting to the ED of the muhimbili national Hospital in Dar es Salaam Tanzania. *Acad Emerg Med*. 2016;23(SUPPL. 1):S99. doi:http://dx.doi.org/10.1111/acem.12974

41. Mwandri M, Hardcastle TC, Sawe H, et al. Trauma burden, patient demographics and care-process in major hospitals in

For peer review only

For peer review only - http://bmjopen.bmj.com/site/about/guidelines.xhtml

Tanzania: A needs assessment for improving healthcare resource management. *African J Emerg Med*. 2020;10(3):111-117. doi:http://dx.doi.org/10.1016/j.afjem.2020.01.010

42. Rajaguru PP, Jusabani MA, Massawe H, Temu R, Sheth NP. Understanding surgical care delivery in Sub-Saharan Africa: a cross-sectional analysis of surgical volume, operations, and financing at a tertiary referral hospital in rural Tanzania. *Glob Heal Res Policy*. 2019;4(1):30. doi:10.1186/s41256-019-0122-2

43. Kimaro GD, Mfinanga S, Simms V, et al. The costs of providing antiretroviral therapy services to HIV-infected individuals presenting with advanced HIV disease at public health centres in Dar es Salaam, Tanzania: Findings from a randomised trial evaluating different health care strategies. *PLoS One*. 2017;12(2):e0171917. doi:http://dx.doi.org/10.1371/journal.pone.0171917

44. Sicuri E, Vieta A, Lindner L, Constenla D, Sauboin C. Economic costs of malaria in children in three Sub-Saharancountries: Ghana, Tanzania and Kenya. *Trop Med Int Heal*. 2011;16(SUPPL. 1):117. http://ovidsp.ovid.com/ovidweb.cgi?T=JS&PAGE=reference&D=emed12&NEWS=N&AN=70589204

45. Riewpaiboon A, Piatti M, Ley B, et al. Cost of illness due to typhoid fever in Pemba, Zanzibar, East Africa. *J Heal Popul Nutr*. 2014;32(3):377-385. http://www.jhpn.net/index.php/jhpn/article/view/2819/1043

46. Mengistu T, Berruti A, Krivelyova A, Swor M, Waite R, Maro G. Cost of providing emergency obstetric care in Tanzania's Kigoma region. *Int J Health Plann Manage*. 2019;34(4):e1510-e1519. doi:http://dx.doi.org/10.1002/hpm.2820

For peer review only

For peer review only - http://bmjopen.bmj.com/site/about/guidelines.xhtml

47. Kabadi GS, Walker R, Donaldson C, Shackley P. The cost of treating stroke in urban and rural Tanzania: A 6-month pilot study. *African J Neurol Sci*. 2013;32(2). http://ajns.paans.org/article.php3?id_article=446

48. James C, Bura M, Ensor T. The costs of delivering health services in Tanzania. Findings from a comprehensive costing analysis.

49. Chaudhury S, Arlington L, Brenan S, et al. Cost analysis of large-scale implementation of the "Helping Babies Breathe" newborn resuscitation-training program in Tanzania. *BMC Health Serv Res*. 2016;16(1):681. http://ovidsp.ovid.com/ovidweb.cgi?T=JS&PAGE=reference&D=emed17&NEWS=N&AN=618644170

50. Guerriero C, Cairns J, Jayaraman S, Roberts I, Perel P, Shakur H. Giving tranexamic acid to reduce surgical bleeding in sub-Saharan Africa: An economic evaluation. *Cost Eff Resour Alloc*. 2010;8:1. doi:http://dx.doi.org/10.1186/1478-7547-8-1

51. Penno EC, Baird SJ, Crump JA. Cost effectiveness of surveillance for bloodstream infections for sepsis management in low resource settings. *Am J Trop Med Hyg*. 2015;93(4):850-860. http://www.ajtmh.org/content/91/5_Suppl_1/74.full.pdf+html

52. Shayo GA, Chitama D, Moshiro C, Aboud S, Bakari M, Mugusi F. Cost-Effectiveness of isoniazid preventive therapy among HIV-infected patients clinicaly screened for latent tuberculosis infection in Dar es Salaam, Tanzania: A prospective Cohort study. *BMC Public Health*. 2017;18(1):35. doi:http://dx.doi.org/10.1186/s12889-017-4597-9

53. Baynes C, Yegon E, Kimaro G, Lusiola G, Kahwa J. The unit and scale-up cost of postabortion care in Tanzania. *Spec Issue*

For peer review only

Sav women's lives through Emerg Obstet care Volunt Fam planning. 2019;7(Suppl. 2):S327-S341. doi:http://dx.doi.org/10.9745/GHSP-D-19-00035

54. The World Bank Group. Rural population (% of total population) - Tanzania | Data. 2021. Accessed July 14, 2021. https://data.worldbank.org/indicator/SP.RUR.TOTL.ZS?locations=TZ

55. Schultz MJ, Dunser MW, Dondorp AM, et al. Current challenges in the management of sepsis in ICUs in resource-poor settings and suggestions for the future. *Intensive Care Med*. 2017;43:612-624. doi:10.1007/s00134-017-4750-z

56. Ranjit S, Kissoon N. Challenges and Solutions in translating sepsis guidelines into practice in resource-limited settings. *Transl Paediatr*. Published online 2021. Accessed August 10, 2021. https://tp.amegroups.com/article/viewFile/66320/pdf

57. Baker T, Khalid K, Acicbe O, McGloughlin S, Amin P. Critical care of tropical disease in low income countries: Report from the Task Force on Tropical Diseases by the World Federation of Societies of Intensive and Critical Care Medicine. *J Crit Care*. 2017;42:351-354. doi:10.1016/J.JCRC.2017.11.028

58. Bion J, Brown C, Gomersall C, Boulanger C, Isherwood P, Schulman D. Society of Critical Care Medicine 50th Anniversary Review Series: Critical Care Education. *Crit Care Med*. Published online 2021:1241-1253. doi:10.1097/CCM.0000000000005130

59. Kairu A, Were V, Isaaka L, Agweyu A, Aketch S, Barasa E. Modelling the cost-effectiveness of essential and advanced critical

For peer review only

For peer review only - http://bmjopen.bmj.com/site/about/guidelines.xhtml

care for COVID-19 patients in Kenya. *medRxiv*. Published online August 24, 2021:2021.08.16.21261894. doi:10.1101/2021.08.16.21261894

60. Kazibwe Joseph, Baker Peter, Baker Tim, et al. We Can Stop Preventable COVID Deaths by Urgently Prioritising Medical Oxygen and Essential Critical Care | Center For Global Development. Center for Global Development. Published February 9, 2021. Accessed August 2, 2021. https://www.cgdev.org/blog/we-can-stop-preventable-covid-deaths-urgently-prioritising-medical-oxygen-and-essential

61. Zimmerman A, Fox S, Griffin R, et al. An analysis of emergency care delays experienced by traumatic brain injury patients presenting to a regional referral hospital in a low-income country. *PLoS One*. 2020;15(10 October 2020):e0240528. doi:http://dx.doi.org/10.1371/journal.pone.0240528

62. Tanzania Medicines and Medical Devices Authority. Guidelines for regulating medical gases. Accessed September 16, 2021. https://www.tmda.go.tz/uploads/publications/en1597391138-GUIDELINES FOR MEDICAL GASES - Final (1)-SK.pdf

63. Powell-Jackson T, King JJC, Makungu C, et al. Infection prevention and control compliance in Tanzanian outpatient facilities: a cross-sectional study with implications for the control of COVID-19. *Lancet Glob Heal*. 2020;8(6):e780-e789. doi:10.1016/S2214-109X(20)30222-9

64. Murthy S, Adhikari NK. ATS REPORTS Global Health Care of the Critically Ill in Low-Resource Settings. *Ann Am Thorac Soc*. 2013;10(5):509-513. doi:10.1513/AnnalsATS.201307-246OT

For peer review only

65. Diaz J V., Riviello ED, Papali A, Adhikari NKJ, Ferreira JC. Global critical care: Moving forward in resource-limited settings. *Ann Glob Heal*. 2019;85(1):3-4. doi:10.5334/aogh.2413

66. Cochrane Reviews. Cochrane Handbook: 10.2.2.4 Language bias. In: *Cochrane*. Accessed September 16, 2021. https://handbook-5-1.cochrane.org/chapter_10/10_2_2_4_language_bias.htm

Authors' contributions

Below is the detailed breakdown of their contribution.

Concept and design: JK, HS, LG, AJ, PT, OS, TB

Development of first draft: JK, HS, LG

Acquisition of data: JK, HS, LG, PT, SG

Analysis and interpretation of data: JK, HS, LG, PT, SG, OS

Drafting of the manuscript: JK, HS, LG

Critical revision of paper for important intellectual content: TB, OS, LG, KK, AJ

Statistical analysis: -

Provision of study materials:

Obtaining funding: TB, OS

Administrative and technical support:

For peer review only

For peer review only - http://bmjopen.bmj.com/site/about/guidelines.xhtml

Supervision: LG, HS

Competing interests

Authors declare no competing interests and the funders did not in any way influence the development of the development of this study.

Funding statement

This work was supported by the Wellcome Trust [221571/Z/20/Z], as part of the 'Innovation in low-and middle-income countries' Flagship.

Data sharing statement

Supporting data is available on request.

Figures

Figure 1: PRISMA Chart

Legend: The PRISMA diagram shows the details the search and selection process applied during our systematic literature search and review.

For peer review only

For peer review only - http://bmjopen.bmj.com/site/about/guidelines.xhtml

Figures

Figure 1: PRISMA Chart

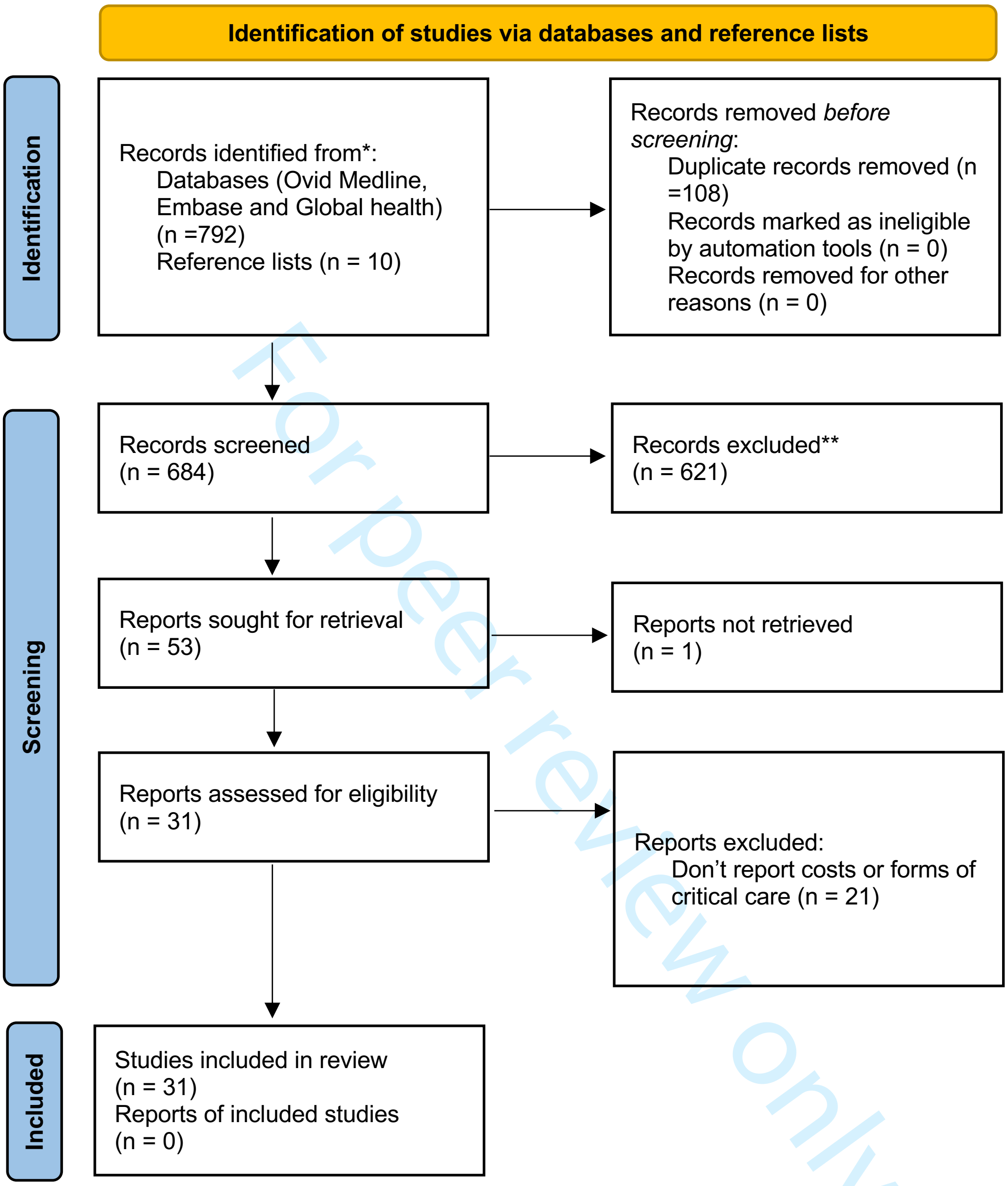

Legend: The PRISMA diagram shows the details the search and selection process applied during our systematic literature search and review.

Based on Page MJ, McKenzie JE, Bossuyt PM, Boutron I, Hoffmann TC, Mulrow CD, et al. The PRISMA 2020 statement: an updated guideline for reporting systematic reviews. BMJ 2021;372:n71. doi: 10.1136/bmj.n71

For peer review only

SUPPLEMENTARY MATERIAL

Supplementary 1: Key concepts and search terms identified for the search.

Search platform: Ovid

Database: Embase Classic+Embase <1947 to 2020 November 18> , Global Health <1973 to 2020 Week 45>, Ovid MEDLINE(R) ALL <1946 to November 17, 2020>
Search Strategy:

----

1 exp intensive care/ or hospital care.ab,ti. or artificial ventilation/ or early goal-directed therapy/ or intensive care nursing/ or newborn intensive care/ or patient monitoring/ or pediatric advanced life support.ab,ti. or emergency.ab,ti. or urgent care.ab,ti. (1538827)
2 (hospital patient or inpatient).ab,ti. (248658)
3 exp hospital patient/ (188072)
4 exp "health care cost"/ or "cost"/ or health economics/ or "drug cost"/ or health care financing/ or "hospital cost"/ or nursing cost.ab,ti. or cost.ab,ti. or costs.ab,ti. (1697788)
5 exp tanzania/ or tanzania.ab,ti. (45585)
6 exp Health Care Costs/ or exp Health Expenditures/ or (cost* or ((health care or health-care or healthcare or medical) adj3 expenditure*) or expense* or hospital resources).ti,ab. or (((resource* or "health service") adj3 ("use" or utilisation or utilization)) or ((financial or economic) and (burden or impact or consequence)) or spending or out of pocket or out-of-pocket or OOP or insurance or absenteeism or (productivity and (loss or lost)) or "economic modelling").ab,ti. (2248416)
7 artificial ventilation.ab,ti. (7728)
8 (malaria or tb or tuberculosis or diagnostics or laboratory or lab or hiv).ab,ti. (3253274)
9 (general care or general ward care or general inpatient care or hospital care).ab,ti. (27507)
10 1 or 2 or 3 or 7 or 8 or 9 (4988953)
11 (economic evaluation or cost effective).ab,ti. (264378)
12 4 or 6 or 11 (2320354)
13 5 and 10 and 12 (1905)
14 limit 13 to yr="2010 -Current" (1295)
15 remove duplicates from 14 (792)

For peer review only

Supplementary 2: Inclusion and exclusion criteria

Inclusion criterion
For a study to be included it should fulfil the following criterion

- Published in English in a peer reviewed journal
- Reports forms or types of critical care offered, critical care services offered and or costs and resources used in the provision of care in Tanzania
- Includes costs from a provider perspective
- Articles published from 2010

Exclusion criterion
Studies will be excluded in case of the following;

- Published in any other languages except English
- Studies carried out outside Tanzania
- Studies with costs estimated from patient perspective
- Studies without full text versions
- Studies published before 2010

Supplementary 3: Study Characteristics (N=31)]

Article	Disease area	Intervention	Study Type	Purpose Type	Geographical area covered	Hospital Type	Facility/hospital Level	Source of data
**Studies reporting on costs**								

For peer review only - http://bmjopen.bmj.com/site/about/guidelines.xhtml

**Article**	**Disease area**	**Intervention**	**Study Type**	**Purpose Type**	**Geographical area covered**	**Hospital Type**	**Facility/hospital Level**	**Source of data**
Baynes et al 2019	Obstetrics	Providing care to women that have had an abortion	Cross sectional	Cost Analysis	Mwanza, Arusha and Zanzibar	NGO & Public	All	Records and interviews
Chris et al 2013	General	Health services	Cross sectional	Cost Analysis	National	All	All	Survey
Kabadi et al 2013	Stroke	Providing stroke care	Longitudinal	Cost Analysis	Mbeya and Dar es Salaam	NGO & Public	Tertiary referral	Survey and project data
Mengistu et al 2019	Direct obstetric complications	Emergency Obstetric and Neonatal Care	Cross sectional	Cost Analysis	Kigoma	Public	Tertiary, district hospital and primary care	Programme & hospital data

For peer review only - http://bmjopen.bmj.com/site/about/guidelines.xhtml

Article	Disease area	Intervention	Study Type	Purpose Type	Geographical area covered	Hospital Type	Facility/hospital Level	Source of data
Mosha et al 2010	Malaria	Diagnostic tools and drugs	Cross sectional	Cost analysis	Kilimanjaro region and Korogwe District, Tanga region	Public	District and dispensaries	Survey
Riewpaiboon et al 2014	Typhoid	Typhoid treatment/care	Longitudinal	Cost Analysis	Pemba and Zanzibar	Public	District hospital	Record review and interviews
Sicuri et al 2011	Malaria	Malaria treatment/care	Longitudinal	Cost Analysis	Not specified	Not specified	Not specified	Survey
Chaudhury et al 2016	Neonatal care	Birth attendants training on	Cross sectional	Cost Analysis	Mbeya	NGO & Public	All	Survey

For peer review only - http://bmjopen.bmj.com/site/about/guidelines.xhtml

Article	Disease area	Intervention	Study Type	Purpose Type	Geographical area covered	Hospital Type	Facility/hospital Level	Source of data
		neonatal resuscitation						
Penno et al 2015	Antimicrobial resistance	Surveillance for Bloodstream Infections for Sepsis Management in low resource setting	Modelling	Economic evaluation	Northern Tanzania	Not specified	Not specified	Laboratory records
Githang'a et 2020	Oncology	Oncology treatment: e.g. chemotherapy, off site	Cross-sectional	Economic evaluation	Mwanza	NGO	Tertiary referral	Hospital data & interviews

For peer review only - http://bmjopen.bmj.com/site/about/guidelines.xhtml

**Article**	**Disease area**	**Intervention**	**Study Type**	**Purpose Type**	**Geographical area covered**	**Hospital Type**	**Facility/hospital Level**	**Source of data**
		radiotherapy, surgery						
Guerriero et al 2010	Blood transfusion	Use of Tranexamic acid as a replacement for blood transfusion	Modelling	Economic evaluation	National	Not specified	Not specified	Literature

For peer review only

Article	Disease area	Intervention	Study Type	Purpose Type	Geographical area covered	Hospital Type	Facility/hospital Level	Source of data
Kimaro et al 2017	Advanced HIV	Providing antiretroviral therapy services to HIV-infected individuals presenting with advanced HIV disease	Cohort	Economic evaluation	Dar es Salaam	Public	Primary health center	Patient survey, health care worker diaries
Phillips et al 2015	Malaria	Malaria Rapid Diagnostic test (mRDT)	Modelling	Economic evaluation	National	All	All	Local program/ records

For peer review only - http://bmjopen.bmj.com/site/about/guidelines.xhtml

Article	Disease area	Intervention	Study Type	Purpose Type	Geographical area covered	Hospital Type	Facility/hospital Level	Source of data
Shayo et al 2017	Tuberculosis	Isoniazid preventive therapy	Cohort	Economic evaluation	Dar es Salaam	NGO, Public and private	Tertiary and primary care	Records and interviews
Wilson et al 2020	Neonatal care	Birth attendants training on neonatal resuscitation (59 participants)	Cross sectional	Financial planning	Zanzibar	Not specified	Not specified	Survey
**Studies reporting on forms and resource availability in critical care**								
Baker et al 2011	Critical care	Emergency and critical care	Cross sectional	Others (structure and resources	National	All	Tertiary, district, and primary care	Survey

For peer review only - http://bmjopen.bmj.com/site/about/guidelines.xhtml

Article	Disease area	Intervention	Study Type	Purpose Type	Geographical area covered	Hospital Type	Facility/hospital Level	Source of data
				available for emergency and critical care)				
Chalya et al 2011	Trauma	Trauma care	Cross sectional	Others (describe patient characteristics and health outcomes)	Mwanza	NGO	Referral hospital	Survey
Mkoka et al 2014	Obstetrics	Emergency obstetric care	Cross sectional	Others (availability	Kongwa	Public	Primary healthcare	Interviews

For peer review only - http://bmjopen.bmj.com/site/about/guidelines.xhtml

Article	Disease area	Intervention	Study Type	Purpose Type	Geographical area covered	Hospital Type	Facility/hospital Level	Source of data
				of medical supplies)				
Mtango et al 2019	Critical care	Critical care	Cross sectional	Others (doctors' experiences of ICU referral)	Dar es salaam	Public	Tertiary referral	Interviews
Murthy et al 2015	Critical care	N/A	Review	Others (estimate the current ICU capacity)	Ifakara, Dar es Salaam and Mwanza	NGO & Public	Tertiary referral	Literature
Mwandri et al 2020	Trauma	Baseline assessment of	Cross sectional	Others (assessing	Mbeya and Dar es Salaam	NGO & Public	Tertiary referral	Hospital registry data

For peer review only - http://bmjopen.bmj.com/site/about/guidelines.xhtml

Article	Disease area	Intervention	Study Type	Purpose Type	Geographical area covered	Hospital Type	Facility/hospital Level	Source of data
		trauma care provision		burden and care process)				
Mwiga & Monaghan 2020	Respiratory illness	Difficult airway trolley	Case study	Others (development of difficulty airway trolley)	Mbeya	NGO	Tertiary referral	Difficult airway trolley
Nicks et al 2012	Emergence medicine	N/A	Case study	Others (structure of health system and care processes)	National	All	All	Literature

For peer review only - http://bmjopen.bmj.com/site/about/guidelines.xhtml

Article	Disease area	Intervention	Study Type	Purpose Type	Geographical area covered	Hospital Type	Facility/hospital Level	Source of data
Papali et al 2019	Critical care	N/A	Review	Others (structure and organisation of adult ICU)	N/A	All	All	Literature
Penoyar et al 2012	Surgery	Surgical care	Cross sectional	Others (capacity of first referral hospitals)	National	All	District, regional and referral	Survey
Rajaguru et al 2019	Surgery	Surgery	Cross sectional	Others (operation and financing of a referral hospital)	Mbeya	NGO	Tertiary referral	Record review

For peer review only - http://bmjopen.bmj.com/site/about/guidelines.xhtml

**Article**	**Disease area**	**Intervention**	**Study Type**	**Purpose Type**	**Geographical area covered**	**Hospital Type**	**Facility/hospital Level**	**Source of data**
Reynolds et al 2012	General (particular injuries, pneumonia and psychosis)	Emergency care	Cross sectional	Others (Barriers to emergency care)	National	Public	Tertiary referral	Interview
Sawe et 2016	General	Emergency treatment	Cross sectional	Others (clinical presentation and resource utilization)	Dar es Salaam	Public	Tertiary referral	Hospital record
Staton et al 2017	Traumatic Brain Injury	Trauma care	Cross sectional	Others (describe	Kilimanjaro	NGO	Referral Hospital	Survey

For peer review only - http://bmjopen.bmj.com/site/about/guidelines.xhtml

Article	Disease area	Intervention	Study Type	Purpose Type	Geographical area covered	Hospital Type	Facility/hospital Level	Source of data
				quality of care for TBI)				
Sylvanus et al 2019	Renal failure	Renal failure treatment and management	Cohort	Others (renal failure case management)	Dar es Salaam	Public	Tertiary referral	Survey
Zimmerman et al 2020	Acute traumatic brain injury	Quantifying the effect on outcomes from delays in the care of trauma	Cross sectional	Others (association of hospital delays and poor outcomes)	Moshi	NGO	Tertiary referral	Hospital data

For peer review only - http://bmjopen.bmj.com/site/about/guidelines.xhtml

Supplementary 4: Availability of resources required for critical care provision

**Resources**	**Study**			
	Penoyar et al 2012 (N=40 hospitals)	*Staton et al 2017 (patients; N=893)*	*Zimmerman et al 2020 (patients; N=3209)*	*Baker et al 2013 (N=10 hospitals)*
Availability of oxygen when needed	42%	22.9%	20.1%	
Oxygen concentrators	75%			
Mask and tubing to connect to oxygen supply	46%			
Pulse oximetry	13%			70%
Oxygen cylinders	35%			
Mechanical ventilators				40%
Oropharyngeal airway-adult	42%			60%
Oropharyngeal airway-paediatric	21%			
Personal protective wear (gloves)	90%			90%
Personal protective wear (aprons)	81%			
Personal protective wear (face masks)	69%			
Personal protective wear (eye protection)	40%			
Consistent running water	37.5%			100%
Consistent running electricity	37.5%			50%
Staff trained in critical care				20%

Supplementary 5: Appraisal Checklist

For peer review only - http://bmjopen.bmj.com/site/about/guidelines.xhtml

Study ID	Author	#1 Question(s)	#2 Perspective	#3 Time horizon	#4 Relevant inputs	#5 Methods for quantities	#6 Data source(s)	#7 Sample size	#8 Discount rate	#9 Sensitivity analysis	#10 Costs reporting
1	Githang'a et 2020	✓	✓	(✓)	✓	✓	✓		✓	✓	✓
2	Mengistu et al 2019	✓	✓	✓	✓	✓	✓			X	✓
3	Wilson et al 2020	✓	✓	**X**	✓	✓	✓			X	✓
4	Kimaro et al 2017	✓	✓	✓	✓	✓	✓			✓	✓
5	Shayo et al 2017	✓	✓	✓	✓	✓	✓			✓	✓
6	Phillips et al 2015	✓	✓	✓	✓	✓	✓			X	✓
7	Penno et al 2015	✓	✓	X	✓	✓	✓			✓	✓
8	Kabadi et al 2013	✓	✓	✓	✓	✓	✓			X	✓
9	Sicuri et al 2011	✓	✓	X	✓	✓	✓			✓	✓

For peer review only

10	Guerriero et al 2010	✓	✓	X	✓	✓	✓			✓	✓
11	Baynes et al 2019	✓	✓	(✓)	✓	✓	✓			✓	✓
12	Riewpaiboon et al 2014	✓	✓	(✓)	✓	✓	✓			✓	✓
13	Sumona et al 2016	✓	✓	(✓)	✓	✓	✓			✓	✓
14	Chris et al 2013	✓	✓	✓	✓	✓	✓			X	✓
15		✓	✓	✓	✓	✓	✓			✓	✓

Key: ✓ addressed; (✓) partially addressed; X Not addressed

For peer review only

Supplementary 6: Cost of human resources

Study	Description	Unit of output	Currency year	Unit cost Price in 2019 (USD)	Unit cost Price in 2019 (TZS)
Mengistu et al 2019	Total personnel for Emergency obstetric care	per patient	2013	103.01	235,711.74
Baynes et al 2019	Cost of Obstetrician/gynecologist on all complications	Per patient	2016	10.00	22,884.06

Baynes et al 2019	Cost of Anesthesiologist on all obstetric complications	Per patient	2016	0.00	0.00
Baynes et al 2019	Cost Anesthetist on all obstetric complications	Per patient	2016	2.23	5,112.40
Baynes et al 2019	Cost of Anesthetist Assistant on all obstetric complications	Per patient	2016	1.81	4,138.61
Baynes et al 2019	Cost of doctor/ medical officer on all obstetric complications	Per patient	2016	15.75	36,030.22
Baynes et al 2019	Cost of assistant medical officer on all obstetric complications	Per patient	2016	5.00	11,442.03
Baynes et al 2019	Cost of clinical officer on all obstetric complications	Per patient	2016	0.85	1,947.58
Baynes et al 2019	Cost of registered nurse or principal enrolled nurse on obstetric all complications	Per patient	2016	9.79	22,397.16
Baynes et al 2019	Cost of enrolled nurse or midwife on all obstetric complications	Per patient	2016	43.62	99,813.44
Baynes et al 2019	Cost of medical attendant on all obstetric complications	Per patient	2016	6.06	13,876.50
Baynes et al 2019	Cost of laboratory technician on all complications	Per patient	2016	3.19	7,303.42

For peer review only - http://bmjopen.bmj.com/site/about/guidelines.xhtml

Baynes et al 2019	Cost of laboratory assistant on all complications	Per patient	2016	1.70	3,895.16
Baynes et al 2019	Cost of Sonographer on all obstetric complications	Per patient	2016	2.87	6,573.08
Baynes et al 2019	Cost of pharmacist on all complications	Per patient	2016	2.55	5,842.74
Baynes et al 2019	Cost of drug dispenser on all complications	Per patient	2016	1.06	2,434.47
Baynes et al 2019	Average personnel costs at primary care level facilities for all complications	Per patient	2016	14.28	32,670.64
Baynes et al 2019	Average personnel care costs at district hospitals for all complications	Per patient	2016	8.97	20,522.62
Baynes et al 2019	Average personnel costs at Regional hospital for all complications	Per patient	2016	30.14	68,968.66
Baynes et al 2019	Average personnel costs for all complications across any level of hospital	Per patient	2016	19.16	43,844.88
Shayo et al 2017	Assistant medical officer	Monthly salary	2012	638.96	1,462,064.84
Shayo et al 2017	Nurse	Monthly salary	2012	313.34	716,994.91
Shayo et al 2017	Health Attendant	Monthly salary	2012	440.41	1,007,738.83

For peer review only - http://bmjopen.bmj.com/site/about/guidelines.xhtml

Shayo et al 2017	Assistant Nurse	Monthly salary	2012	338.60	774,793.40
Shayo et al 2017	Nurse Counsellors	Monthly salary	2012	819.44	1,875,051.02
Shayo et al 2017	Medical Doctors	Monthly salary	2012	1473.56	3,371,810.40
Shayo et al 2017	Medical Specialists	Monthly salary	2012	2721.79	6,228,019.13
Shayo et al 2017	Laboratory Scientific Officer	Monthly salary	2012	1106.07	2,530,924.80
Shayo et al 2017	Laboratory technician	Monthly salary	2012	819.44	1,875,051.02
Shayo et al 2017	Pharmacist	Monthly salary	2012	1106.07	2,530,924.80
Shayo et al 2017	Pharmacy technician	Monthly salary	2012	819.44	1,875,051.02

For peer review only - http://bmjopen.bmj.com/site/about/guidelines.xhtml

PRISMA 2020 Checklist

Section and Topic	Item #	Checklist item	Location where item is reported
**TITLE**			
Title	1	Identify the report as a systematic review.	Title page
**ABSTRACT**			
Abstract	2	See the PRISMA 2020 for Abstracts checklist.	Pages 2 & 3
**INTRODUCTION**			
Rationale	3	Describe the rationale for the review in the context of existing knowledge.	Page 5 & 6 Lines 79 - 91
Objectives	4	Provide an explicit statement of the objective(s) or question(s) the review addresses.	Page 6, Lines 93 - 94
**METHODS**			
Eligibility criteria	5	Specify the inclusion and exclusion criteria for the review and how studies were grouped for the syntheses.	Page 8, Lines 123-125
Information sources	6	Specify all databases, registers, websites, organisations, reference lists and other sources searched or consulted to identify studies. Specify the date when each source was last searched or consulted.	Page 6, Lines 105-106
Search strategy	7	Present the full search strategies for all databases, registers and websites, including any filters and limits used.	Supplementary 1
Selection process	8	Specify the methods used to decide whether a study met the inclusion criteria of the review, including how many reviewers screened each record and each report retrieved, whether they worked independently, and if applicable, details of automation tools used in the process.	Page 8, Lines 118-120
Data collection process	9	Specify the methods used to collect data from reports, including how many reviewers collected data from each report, whether they worked independently, any processes for obtaining or confirming data from study investigators, and if applicable, details of automation tools used in the process.	Page 8, Lines 128-131
Data items	10a	List and define all outcomes for which data were sought. Specify whether all results that were compatible with each outcome domain in each study were sought (e.g. for all measures, time points, analyses), and if not, the methods used to decide which results to collect.	Page 9, Lines 133-138
	10b	List and define all other variables for which data were sought (e.g. participant and intervention characteristics, funding sources). Describe any assumptions made about any missing or unclear information.	Page 8, Lines 128-131
Study risk of bias assessment	11	Specify the methods used to assess risk of bias in the included studies, including details of the tool(s) used, how many reviewers assessed each study and whether they worked independently, and if applicable, details of automation tools used in the process.	Page 9, Lines 142-146
Effect measures	12	Specify for each outcome the effect measure(s) (e.g. risk ratio, mean difference) used in the synthesis or presentation of results.	N/A
Synthesis methods	13a	Describe the processes used to decide which studies were eligible for each synthesis (e.g. tabulating the study intervention characteristics and comparing against the planned groups for each synthesis (item #5)).	Page 9, Lines 133-137
	13b	Describe any methods required to prepare the data for presentation or synthesis, such as handling of missing summary statistics, or data conversions.	N/A
	13c	Describe any methods used to tabulate or visually display results of individual studies and syntheses.	Page 9, Lines 133-137
	13d	Describe any methods used to synthesize results and provide a rationale for the choice(s). If meta-analysis was performed, describe the model(s), method(s) to identify the presence and extent of statistical heterogeneity, and software package(s) used.	N/A
	13e	Describe any methods used to explore possible causes of heterogeneity among study results (e.g. subgroup analysis, meta-regression).	N/A

For peer review only - http://bmjopen.bmj.com/site/about/guidelines.xhtml

PRISMA 2020 Checklist

Section and Topic	Item #	Checklist item	Location where item is reported
	13f	Describe any sensitivity analyses conducted to assess robustness of the synthesized results.	N/A
Reporting bias assessment	14	Describe any methods used to assess risk of bias due to missing results in a synthesis (arising from reporting biases).	N/A
Certainty assessment	15	Describe any methods used to assess certainty (or confidence) in the body of evidence for an outcome.	Page 9, Lines 142-146
**RESULTS**			
Study selection	16a	Describe the results of the search and selection process, from the number of records identified in the search to the number of studies included in the review, ideally using a flow diagram.	Figure 1
	16b	Cite studies that might appear to meet the inclusion criteria, but which were excluded, and explain why they were excluded.	N/A
Study characteristics	17	Cite each included study and present its characteristics.	Table 2
Risk of bias in studies	18	Present assessments of risk of bias for each included study.	Supplementary
Results of individual studies	19	For all outcomes, present, for each study: (a) summary statistics for each group (where appropriate) and (b) an effect estimate and its precision (e.g. confidence/credible interval), ideally using structured tables or plots.	Tables 3-6
Results of syntheses	20a	For each synthesis, briefly summarise the characteristics and risk of bias among contributing studies.	N/A
	20b	Present results of all statistical syntheses conducted. If meta-analysis was done, present for each the summary estimate and its precision (e.g. confidence/credible interval) and measures of statistical heterogeneity. If comparing groups, describe the direction of the effect.	N/A
	20c	Present results of all investigations of possible causes of heterogeneity among study results.	N/A
	20d	Present results of all sensitivity analyses conducted to assess the robustness of the synthesized results.	N/A
Reporting biases	21	Present assessments of risk of bias due to missing results (arising from reporting biases) for each synthesis assessed.	N/A
Certainty of evidence	22	Present assessments of certainty (or confidence) in the body of evidence for each outcome assessed.	N/A
**DISCUSSION**			
Discussion	23a	Provide a general interpretation of the results in the context of other evidence.	Pages 40-44, Lines 294-369
	23b	Discuss any limitations of the evidence included in the review.	Pages 45-46, Lines 381- 391
	23c	Discuss any limitations of the review processes used.	Pages 45-46, Lines 381- 391
	23d	Discuss implications of the results for practice, policy, and future research.	Page 46, Lines 395-402
**OTHER INFORMATION**			
Registration and protocol	24a	Provide registration information for the review, including register name and registration number, or state that the review was not registered.	Page 6, Line 100
	24b	Indicate where the review protocol can be accessed, or state that a protocol was not prepared.	Page 6, Lines 100-101

For peer review only

PRISMA 2020 Checklist

Section and Topic	Item #	Checklist item	Location where item is reported
	24c	Describe and explain any amendments to information provided at registration or in the protocol.	N/A
Support	25	Describe sources of financial or non-financial support for the review, and the role of the funders or sponsors in the review.	Page 58, Lines 579-580
Competing interests	26	Declare any competing interests of review authors.	Page 58, Line 575
Availability of data, code and other materials	27	Report which of the following are publicly available and where they can be found: template data collection forms; data extracted from included studies; data used for all analyses; analytic code; any other materials used in the review.	Available

Based on Page MJ, McKenzie JE, Bossuyt PM, Boutron I, Hoffmann TC, Mulrow CD, et al. The PRISMA 2020 statement: an updated guideline for reporting systematic reviews. BMJ 2021;372:n71. doi: 10.1136/bmj.n71

For peer review only

BMJ Open

Resource use, availability and cost in the provision of critical care in Tanzania: A systematic review

Journal:	*BMJ Open*
Manuscript ID	bmjopen-2021-060422.R1
Article Type:	Original research
Date Submitted by the Author:	31-Aug-2022
Complete List of Authors:	Kazibwe, Joseph; London School of Hygiene & Tropical Medicine, Global Health and Development<br>Shah, Hiral A.; Imperial College London, Department of Infectious Disease Epidemiology; Center for Global Development<br>Kuwawenaruwa, A; Ifakara Health Institute, Health System Impact Evaluation and Policy Unit<br>Schell, Carl Otto; Karolinska Institutet, Department of Global Public Health; Uppsala University<br>Khalid, Karima; Muhimbili University of Health and Allied Sciences, Department of Anaesthesia and Critical Care<br>Tran, Phuong Bich; University of Antwerp, Department of Family and Population Health<br>Ghosh, Srobana; Center for Global Development, Global Health Department<br>Baker, Tim; London School of Hygiene & Tropical Medicine, Department of Clinical Research; Ifakara Health Institute, Department of Emergency Medicine<br>GUINNESS, LORNA; Center for Global Development
**Primary Subject Heading**:	Emergency medicine
Secondary Subject Heading:	Intensive care
Keywords:	INTENSIVE & CRITICAL CARE, HEALTH ECONOMICS, Health policy < HEALTH SERVICES ADMINISTRATION & MANAGEMENT

SCHOLARONE™
Manuscripts

I, the Submitting Author has the right to grant and does grant on behalf of all authors of the Work (as defined in the below author licence), an exclusive licence and/or a non-exclusive licence for contributions from authors who are: i) UK Crown employees; ii) where BMJ has agreed a CC-BY licence shall apply, and/or iii) in accordance with the terms applicable for US Federal Government officers or employees acting as part of their official duties; on a worldwide, perpetual, irrevocable, royalty-free basis to BMJ Publishing Group Ltd ("BMJ") its licensees and where the relevant Journal is co-owned by BMJ to the co-owners of the Journal, to publish the Work in this journal and any other BMJ products and to exploit all rights, as set out in our licence.

The Submitting Author accepts and understands that any supply made under these terms is made by BMJ to the Submitting Author unless you are acting as an employee on behalf of your employer or a postgraduate student of an affiliated institution which is paying any applicable article publishing charge ("APC") for Open Access articles. Where the Submitting Author wishes to make the Work available on an Open Access basis (and intends to pay the relevant APC), the terms of reuse of such Open Access shall be governed by a Creative Commons licence – details of these licences and which Creative Commons licence will apply to this Work are set out in our licence referred to above.

Other than as permitted in any relevant BMJ Author's Self Archiving Policies, I confirm this Work has not been accepted for publication elsewhere, is not being considered for publication elsewhere and does not duplicate material already published. I confirm all authors consent to publication of this Work and authorise the granting of this licence.

For peer review only

Resource use, availability and cost in the provision of critical care in Tanzania: A systematic review

Joseph Kazibwe,
1. Global Health and Development Department, London School of Hygiene and Tropical Medicine, London UK
2. ORCID - https://orcid.org/0000-0002-8315-1503

Joseph.Kazibwe@lshtm.ac.uk

Hiral A. Shah,
1. MRC Centre for Global Infectious Disease Analysis, Department of Infectious Disease Epidemiology, School of Public Health, Imperial College London, UK.
2. Center for Global Development, London, UK.
3. ORCID - https://orcid.org/0000-0003-0204-451X

August Kuwawenaruwa,
1. Health System Impact Evaluation and Policy Unit, Ifakara Health Institute United Republic of Tanzania

Carl Otto Schell,
1. Department of Global Public Health, Karolinska Institutet, Stockholm, Sweden
2. Centre for Clinical Research Sörmland, Uppsala University, Eskilstuna, Sweden
ORCID - https://orcid.org/0000-0002-7904-1336

Karima Khalid,
1. Department of Anaesthesia and Critical Care, Muhimbili University of Health and Allied Sciences, Dar es Salaam, Tanzania
2. Ifakara Health Institute, Dar-es-salaam, Tanzania
ORCID - https://orcid.org/0000-0001-9917-9853

Phuong Bich Tran,

1. Department of Family and Population Health, University of Antwerp, Antwerpen, Belgium

ORCID - https://orcid.org/0000-0003-2119-998X

Srobana Ghosh,
1. Global Health Department, Center for Global Development, London, UK

Tim Baker,
1. Department of Clinical Research, London School of Hygiene & Tropical Medicine, UK
2. Ifakara Health Institute, Dar es Salaam, Tanzania

For peer review only - http://bmjopen.bmj.com/site/about/guidelines.xhtml

3. Department of Emergency Medicine, Muhimbili University of Health and Allied Sciences, Dar es Salaam, Tanzania
4. Department of Global Public Health, Karolinska Institutet, Stockholm, Sweden
ORCID: 0000-0001-8727-7018

Lorna Guinness
1. Center for Global Development, London, UK
ORCID: https://orcid.org/0000-0002-1013-4200

Key words: Critical care, Costs of critical care, forms of critical care, Resources, Low- and middle-income countries (LMICs), Tanzania

Abstract

Objectives

Critical care is essential in saving lives of critically ill patients, however, provision of critical care across lower resource settings can be costly, fragmented and heterogenous. Despite the urgent need to scale-up the provision of critical care, little is known about its availability and cost. Here, we aim to systematically review and identify reported resource use, availability and costs for the provision of critical care and the nature of critical care provision in Tanzania.

Methods

The systematic review followed the Preferred Reporting Items for Systematic Reviews and Meta-Analyses (PRISMA) guidelines. We searched Medline, Embase and Global Health databases. We included studies that reported on provision of critical care, cost and availability of resources used in the provision of critical care published after 2010. Costs were adjusted and reported in 2019 USD and TZS using the World Bank GDP deflators. The Reference Case for Estimating the Costs of Global Health Services and Interventions was used to assess quality of included studies.

For peer review only - http://bmjopen.bmj.com/site/about/guidelines.xhtml

Results

A total 31 studies were found to fulfil the inclusion and exclusion criteria. Critical care identified in Tanzania was categorised into: ICU delivered critical care and non-ICU critical care. The availability of ICU delivered critical care was limited to urban settings whereas non-ICU critical care was found in rural and urban settings. Paediatric critical care equipment were more scarce than equipment for adults. 15 studies reported on the costs of services related to critical care yet no study reported an average or unit cost of critical care. Costs of medication, equipment (e.g. oxygen, PPE), services, and human resources were identified as inputs to specific critical care services in Tanzania.

Discussion

There is limited evidence on the resource use, availability and costs of critical care in Tanzania. There is a strong need for further empirical research on critical care resources availability, utilization and costs across specialties and hospitals of different level in low- and middle-income countries like Tanzania to inform planning, priority setting and budgeting for critical care services. PROSPERO registration number: CRD42020221923

Strength and limitations of this study

- This study followed the Preferred Reporting Items for Systematic Reviews and Meta-Analyses PRISMA guidelines to produce the first systematic review of the literature around the costs and resources used in critical care and forms of critical care in a lower resource setting such as Tanzania.

For peer review only - http://bmjopen.bmj.com/site/about/guidelines.xhtml

- A broad and comprehensive search strategy was designed to ensure all studies on resource utilisation and costs of critical care were identified despite its heterogeneous nature.
- This study used a fully articulated population, intervention, comparison and outcome framework to develop the search strategy and identify key search terms.
- To address the likelihood of limited relevant data on critical care in Tanzania, we selected a set of diseases likely to require critical care (eg, malaria and pneumonia) to include in our search strategy which may have introduced some selection bias.
- The availability of literature was limited in this lower-middle income country setting.

For peer review only

For peer review only - http://bmjopen.bmj.com/site/about/guidelines.xhtml

Background

Critical care is an essential element of healthcare whose importance has been highlighted globally during the COVID-19 pandemic with countries experiencing overwhelming numbers of critically ill patients [1–3]. As a result, the care of critical illness has become an urgent point of focus in global health policy. COVID-19 notwithstanding, critical illness remains challenging driven by the large burden of injuries, infections and noncommunicable diseases [4,5]. There are an estimated 45 million cases of critical illness in adults in the word each year[4] yet the limited capacity to deliver critical care, especially in resource constrained settings, is likely to continue [6].

Critical care is the identification, monitoring and treatment of patients with critical illness through the initial and sustained support of vital organ functions[7]. It entails enhanced monitoring, treatment and attention, due to a life-threatening illness or injury[8]. There have been great advances in the provision of critical care for example invasive and non-invasive monitoring techniques, mechanical ventilation (MV), and renal replacement therapy, among others, resulting in reduced mortality rates in patients with critical illness, especially in high income countries[9].

However, such critical care is provided in sophisticated intensive care units using equipment which are costly to procure and maintain[10]. This makes it difficult for low resource settings to acquire such equipment to meet the needs of their populations and has led to recommendations for the development of more affordable critical care models that can be provided in any hospital settings and to all critically ill patients [1,11,12]. Moreover, most critically ill patients do not need critical care at ICU-level[13] and large unmet needs of even basic lifesaving critical care have been found in low-

For peer review only - http://bmjopen.bmj.com/site/about/guidelines.xhtml

and middle-income countries (LMICs)[14–16]. With various national and international stakeholders calling for critical care scale-up[17], it is necessary to understand the resources needed in the provision of critical care and their respective costs. Unfortunately, knowledge on the current availability of resources and the costs of critical care remains sparse[1,18,19]. There is no systematic review that examines the available resources, utilisation and costs of critical care generally[20] nor in LMIC settings.

To inform the planning and scale up of critical care provision in a low-income country setting, this study takes a systematic approach to review available knowledge on the current resource availability, utilisation and cost in the provision of critical care in Tanzania[20].

Methods

The systematic review followed the Preferred Reporting Items for Systematic Reviews and Meta-Analyses (PRISMA) guidelines[21] and the protocol was registered with PROSPERO; registration number: CRD42020221923[20]. The full methodological protocol used in this review is available elsewhere[20]. For this study, critical care is defined as the identification, monitoring and treatment of patients with critical illness through the initial and sustained support of vital organ functions[7]. It involves enhanced monitoring, treatment and attention required by a patient as a result of the life-threatening aspects of an illness or injury[8]. Some aspects of critical care can be provided in any location e.g. monitoring for vital signs and respiratory support. However, an ICU is a well-established unit intended to provide specialist critical care that sustains life of patients during

For peer review only

periods when a patient is experiencing a life-threatening condition. ICU critical care is critical care offered in an ICU while a non-ICU critical care is critical care offered in a place that is not gazetted as an ICU.

Search strategy and selection process

Following PRISMA guidelines, 3 electronic databases (i.e. Medline, Embase and Global Health) were systematically searched for articles published since 2010 [22]. A ten-year time frame was considered to ensure that findings reflect the present-day resource utilization paradigm as resources used and their costs vary as technology and clinical guidelines evolve. Bibliographies of included articles were reviewed to find relevant articles that fulfil the inclusion criteria but had not been identified. Google and Google Scholar were also used to search for published articles that may not have been indexed within the databases. As internet search engines typically return several thousand results, the searches were restricted to the first fifty hits and links to potentially relevant material were accessed[23].

Table 1PICO (Population, Intervention, Comparator, Outcome) Framework used to inform the search strategy

Element	Description
Population	Any patient in need of critical care
Intervention	Inpatient services that could form part of critical care provision
Comparison	No critical care
Outcomes	Critical care services available<br><br>Cost of the different inputs to critical care provision per patient

For peer review only - http://bmjopen.bmj.com/site/about/guidelines.xhtml

	Quantity and type of resources used in provision of critical care per patient

The PICO framework (table 1) describes major components used to identify key words. Other concepts and search terms are listed in the supplementary material. Key words included critical care, critical illness, resource use and setting. To ensure that no costings were missed, we further added hospital care and selected condition with high disease burden in Tanzania to the search string (for example neonatal disorders, HIV, Malaria and TB[24]), on the premise that these are conditions that have a high risk of critical illness. See supplementary 1 for detailed key words.

Eligibility criteria

A study was considered eligible if it was published in English and reported on the provision of critical care[25], services offered under critical care, resources and or costs incurred by the health system or health providers in providing critical care in Tanzania. A detailed inclusion/ exclusion criterion can be found in supplementary 2.

Study selection

The selection process followed the PRISMA guidelines. The articles identified during the search were retrieved and uploaded to Rayyan QCRI software[26]. The software was used to remove the duplicates. A standard process of screening titles and abstracts and full text reading was followed in assessment of eligibility of articles. Although some papers were focussed on a specific condition, those that described including the costs of critical care and/or emergency related to that condition were selected. While many diagnostics are not specific to critical care, they can be used in the delivery of critical care. We therefore also included papers that reported on diagnostic costs.

For peer review only - http://bmjopen.bmj.com/site/about/guidelines.xhtml

During the assessment of eligibility of the articles, two independent researchers of LG, HAS, SG, PBT and JK first reviewed the title and abstract. Articles that were identified as still eligible were then assessed through full text review by at least two researchers (SG, JK, HAS, PBT and LG). All conflicts were discussed and agreed on through consensus with third researcher (LG or HAS).

Data extraction and synthesis

A bespoke data extraction form was developed and used to record extracted data. The extracted data included author, year of publication, context (location, setting- urban or rural, type of facility, level of facility), critical care services offered, critical care equipment available, costing perspective, costing year, currency used, type of provider, payer, source of cost data, costing time frame, direct medical costs, resources used.

A narrative synthesis of extracted data was conducted to report an overview of study characteristics of the eligible studies. We described the characteristics of critical care provision as identified in the literature and resources available for the provision of critical care in Tanzania. To facilitate comparisons, all reported costs were adjusted to 2019 prices using the GDP deflators for Tanzania[27] and reported in USD and TZS. Cost data was collated first at the service level - by specific patient level service delivered. Then costs and resources used were categorized into groups based on a standard input-wise categorisation, for example, human resources, diagnostics, services, consumables and medication.

Quality assessment of individual studies

For peer review only - http://bmjopen.bmj.com/site/about/guidelines.xhtml

The quality of the included articles was assessed by two in dependent researchers using an adaptation of the Reference Case for Estimating the Costs of Global Health Services and Interventions[28], which provides a framework for quality assessment and data extraction. We used this framework to check the transparency of the authors in reporting what they did and identify likely sources of bias. With the framework we described the following; any sub group or population analysis done, statistical methods used to establish differences in unit costs by sub-group, determinants of cost, multivariate statistical methods used to analyze cost functions, sensitivity analyses conducted, list possible sources of bias, aspects of the cost estimates that would limit generalizability of results to other constituencies, all pecuniary and non-pecuniary interests of the study. Furthermore, methodological quality of studies that reported costs were further assessed by an appraisal checklist by Adawiyah et al[29]. We used ten parameters to further assess the methodological quality which included research question being tackled, perspective, time horizon, relevant inputs, methods for quantities, data sources, sample size, discount rate, sensitivity analysis, and reporting of costs. Any discrepancies were addressed by a joint re-evaluation of the article among 3 authors.

Patient and Public Involvement

This systematic review did not involve any patient nor public in the design, data extraction and analysis. However, the findings of this systematic review will be made available to the public through an open access manuscript.

[Insert figure 1: PRISMA Chart]

Results

For peer review only - http://bmjopen.bmj.com/site/about/guidelines.xhtml

Study characteristics

A total of 792 articles were found using the search and 10 articles from the reference list review. Thirty-one studies fulfilled the inclusion criteria (figure 1), of which 14 reported on the costs associated with critical care in Tanzania (please see supplementary 3: table of study characteristics (N=31)). The disease areas covered by eligible studies included; oncology (n=1), trauma (n=4), obstetrics (n=3), neonatal/child care (n=2), HIV (n=1), renal failure (n=1), TB (n=1), malaria (n=3), stroke (n=1) and typhoid (n=1), antimicrobial resistance (n=1) surgery (n=2) respiratory illness (n=1), blood transfusion (n=1), no disease or area specific (n=8). The majority of studies were cross sectional in design (n=20) and reported on the location of critical care and critical care services available.

Most studies were conducted in a specific region: the Coast Zone (n = 7), Lake Zone (n = 3), Northern Highland Zone (n = 3), Southern Highland Zone (n = 3) and Central Zone (n = 1). A zone is a geographical area made up of districts within a given health region. Seven studies were nationally representative (n = 7); two studies did not specify a given region. Three studies were carried out in rural settings while the rest were urban or mixed settings. Of the studies that specifically reported on critical care provision, thirteen of the fifteen studies were carried out in urban settings, two of the fifteen studies were carried out in both rural and urban settings and no study was exclusively carried out in a rural setting. Nine studies specified that they were conducted in public (government owned) facilities and six specified non-governmental organisations including faith/missionary-based hospitals.

For peer review only - http://bmjopen.bmj.com/site/about/guidelines.xhtml

Critical care provision in Tanzania

The structure of the publicly funded health system in Tanzania is comprised of six levels[30] starting with the village health post (lowest level), dispensaries, health centres, district hospitals, regional hospitals and tertiary referral hospitals (the highest level) and care is delivered by public, faith/missionary-based providers and private providers [30–43]. Critical care is usually offered at the hospital level only, that is the district hospital, regional hospitals and referral hospitals [30–35,37–44]. In addition, Nicks et al state that district hospitals offer basic forms of critical care while referral hospitals offer critical care in ICUs[30].

Based on the literature, we further categorise critical care into “Critical care delivered in an ICU” and “Critical care delivered outside the ICU”. ICU delivered critical care services included intubation and ventilation. For example, one study found that 54.2% of all ICU patients were intubated or ventilated[42]. In total, seven studies reported on ICU delivered critical care [32,33,39,40,42,43]. The ICUs were all based in tertiary referral hospitals in Tanzania including regional hospitals. For example, Murthy et al state that St. Francis Hospital in Ifakara region had 10 ICU beds and Sekou Toure Regional Referral Hospital in Mwanza had 8 ICU beds[37]. The district hospitals, for which studies were available, did not provide ICU delivered critical care [38].

Critical care outside the ICU was reported to be delivered either in operating theatres or in emergency units and general wards. For example, operating theatres at Kilimanjaro Christian Medical Centre (KCMC) offered critical care for cases like trauma and sepsis[45]. Theatres in lower-level hospitals like district hospitals often offer initial critical care before referring the patient to higher level hospitals that can offer ICU based critical care. Although district hospitals

For peer review only - http://bmjopen.bmj.com/site/about/guidelines.xhtml

and some regional hospitals were reported not to have ICUs, these facilities were reported to offer critical care in the general wards[38]. Some hospitals without ICUs designated areas within general wards to cater for the critically ill patients where arterial blood gas and central venous pressure monitoring are done[38]. Administration of critical care related medicines like Mannitol, and airway management were carried out in general wards.[43] Furthermore, Staton et al found that some critical care services including intubation were performed in the emergency units[43].

Availability of resources required for critical care

The majority of eligible studies reported on the known resources used for critical care (n=21). However, many studies did not report the extent to which the resources were readily available at a given hospital (n=18). Three studies reported on availability and use of critical care resources and indicate that their availability varies between hospitals and is sometimes unreliable or inconsistent (supplementary 4). Two studies find that there is a scarcity of trained staff in critical care[38,41]. Further, the availability of critical care equipment was heterogenous, with scarcity in the areas of mask and tubing, pulse oximetry, oxygen cylinders, adult and paediatric oropharyngeal airway, PPE eye protection while gloves were abundant. In addition, a greater scarcity in paediatric than adult critical care equipment was identified [38,41].

Quality assessment

Of the 31 studies, 14 reported on the resource use and critical care costs and were appraised for quality using the Adawiyah et al adapted appraisal checklist[29] (Supplementary 5). All 14 studies were found to have clear research questions, mentioned the specific costing perspective, included relevant inputs, clearly stated methods for quantifying resources and reported costs. Four studies

For peer review only - http://bmjopen.bmj.com/site/about/guidelines.xhtml

did not specify the time horizon while four studies partially addressed it. The majority of the studies (n=8) carried out some form of sensitivity analysis. All studies that collected primary data stated sample size but did not mention how it was determined and whether it was sufficient.

Resource use and cost of resources associated with provision of critical care

Types of costs reported and sources of data

ublished costs of critical care were reported as economic costs (n = 8) or financial costs (n = 6), using a mix of bottom up (n = 9) and top down (n = 5) approaches. Sources of the costs were local data collected from hospital records, surveys and price lists. Surveys (including key informant interviews) were a common method of data collection (n = 9). Three studies used patient level data on resource use to inform costs[46–48]. Hospital or laboratory records were used in 6 of the 14 studies. Some studies used both survey and records review as the source of data (n = 6). Two studies used international literature as a source of data (n =2). Unit costs reported included cost per program, cost per patient, cost per visit, cost per patient day, cost per episode, cost per bed day, inpatient cost, total annual cost, cost per case.

For peer review only

For peer review only - http://bmjopen.bmj.com/site/about/guidelines.xhtml

Table 2: Types of costs reported and sources of data

Study	Type of costs*	Real world /guideline cost	Full/incremental cost	Costing approach**	Source of cost data
Githang'a et 2020	Financial	Real world costs	Full costs	Mixed	Hospital data
Mengistu et al 2019	Financial	Real world costs	Full costs	Top-down	Programme, hospital and health center data
Wilson et al 2020	Financial	Real world costs	Full costs	Bottom-up	Staff Survey (N=59)
Kimaro et al 2017	Economic	Real world costs	Full costs	Bottom-up	Patient survey, diaries (N=870)
Shayo et al 2017	Economic	Real world costs	Full costs	Bottom-up	Records, government standards and market price survey
Phillips et al 2015	Economic	Real world costs	Full costs	Top down	Local program records
Penno et al 2015	Economic	Real world costs	Full costs	Bottom-up	Laboratory records
Kabadi et al 2013	Economic	Real world costs	Full costs	Bottom-up	Survey (N=16) and project data
Sicuri et al 2011	Economic	Real world costs	Full costs	Bottom-up	Patient Survey (N=150)
Guerriero et al 2010	Economic	Real world costs	Full costs	N/A	Literature
Baynes et al 2019	Financial	Real world costs	Full costs	Bottom-up	Health facility Records (N=31) and key informant interviews (N=124)
Riewpaiboon et al 2014	Economic	Real world costs	Full costs	Bottom-up	Record review and patient survey (N=17)
Sumona et al 2016	Financial	Real world costs	Full costs	Bottom-up	Health facility Survey (N=386)
James et al 2013	Financial	Real world costs	Full costs	Top-down	Facility Survey (N=155)

*Economic costs are costs that capture opportunity i.e. the value of the forgone option/item including any donations or volunteer time, financial costs are the amount/money paid for a good or service.

**Top down cost estimation method is the estimation of cost by dividing the total cost by the quantity of services delivered or activities carried out, bottom up cost estimation method is based on the valuation of all resources used in the process of delivering/providing a good or service. Mixed approaches use top down methods for certain cost categories and bottom up for other categories where more precision is required.

Cost of services offered to patients

Table 3: Cost of services offered to patients

Study	Description	Unit cost of output	Currency year	Unit cost Price in 2019 (USD)	Unit cost Price in 2019 (TZS)
Mengistu et al 2019	Clinical care for emergency obstetric case	per case	2013	143.85	329,166.
Mengistu et al 2019	Average cost per surgical patient	per patient	2013	285.08	652,333.26
Mengistu et al 2019	Average cost per patient undergoing caesarean	Per patient	2013	471.28	1,078,396.4 1
Riewpaiboon et al 2014	Inpatient hospital cost	Per patient per day	2010	91.78	107.49
Sicuri et al 2011	Episode of Malaria hospitalisation and Severe anaemia	per episode	2009	57.12	130,704.61
Sicuri et al 2011	Episode of Cerebral malaria	per episode	2009	22.67	51,864.33
Sicuri et al 2011	Episode of Cerebral malaria and neurological sequela	per episode	2009	47.58	108,879.84
Kabadi et al 2013	Hospitalisation of stroke patient (Inpatient, Drugs, Registration)- Rural area	per patient	2005-2006	12.76	29,200.18

For peer review only - http://bmjopen.bmj.com/site/about/guidelines.xhtml

Kabadi et al 2013	Hospitalisation of stroke patient (Inpatient, Drugs, Registration)- Urban area	per patient	2005-2006	31.17	71,317.89
Kabadi et al 2013	Physiotherapy for stroke- urban area	per patient	2005-2006	44.88	102,697.20
Kabadi et al 2013	Hospitalisation of stroke patient (Inpatient, Drugs, Registration)- average	per patient	2005-2006	18.51	42,361.25
Kabadi et al 2013	Physiotherapy for stroke- average	per patient	2005-2006	14.03	32,093.43
James, Bura & Ensor 2013	Health centre: overall	Inpatient day	2011/12	18.03	41,261.22
James, Bura & Ensor 2013	Health centre: public	Inpatient day	2011/12	18.98	43,432.74
James, Bura & Ensor 2013	health centre: NGO	Inpatient day	2011/12	17.29	39,564.68
James, Bura & Ensor 2013	Hospital: all	Inpatient day	2011/12	25.95	59,387.93
James, Bura & Ensor 2013	Hospital: public	Inpatient day	2011/12	18.54	42,422.55
James, Bura & Ensor 2013	Hospital: NGO	Inpatient day	2011/12	15.76	36,062.52
James, Bura & Ensor 2013	Hospital: private	Inpatient day	2011/12	68.05	155,721.31
James, Bura & Ensor 2013	Hospital regional	Inpatient day	2011/12	17.33	39,653.32
James, Bura & Ensor 2013	Hospital - ARI-pneumonia	per case treated	2011/12	104.33	238,736.91
James, Bura & Ensor 2013	Hospital - Hypertension	per case treated	2011/12	253.98	581,166.07
James, Bura & Ensor 2013	Hospital - Myocardial infarction	per case treated	2011/12	268.57	614,533.21

For peer review only

For peer review only - http://bmjopen.bmj.com/site/about/guidelines.xhtml

Although many studies in table 3 reported costs of differing disease specific (e.g. malaria and anaemia) or general hospital services (e.g. inpatient and outpatient care) that included treatment of critical illness, no study reported the specific cost of the treatment of the critical care. A nationally representative study of general hospital services including critical care found that unit costs vary by provider ownership (public, NGO and private) and level of health facility (regional vs primary care) [49]. Services provided at private hospitals were more expensive than the same services received at public hospitals. NGO hospitals were less expensive than public hospitals[49].

Cost of Equipment, consumables and medication

Table 4: Cost of Equipment, consumables and medication

Study	Description	Unit cost of output	Currency year	Unit cost Price in 2019 (USD)	Unit cost Price in 2019 (TZS)
**Equipment**					
Chaudhury et al 2016	Mannequin	per piece	2014	76.03	173,970.65
Chaudhury et al 2016	Bag-mask device	per piece	2014	16.29	37,279.43
Chaudhury et al 2016	Penguin sucke	per piece	2014	3.26	7,455.89
Mengistu et al 2019	Equipment needed for emergency obstetric care	per patient	2013	90.02	205,994.65
**Consumables**					
Mengistu et al 2019	Other supplies for emergency obstetric care	per patient	2013	28.68	65,635.80
Mengistu et al 2019	Blood supplies	per patient	2013	0.71	1,620.01
Guerriero et al 2010	Consumables needed in the administration of tranexamic acid (10ml syringe,100ml bag of saline, large gauge needle)	Per dose	2007	2.00	4,576.41
**Medication**					
Analgesics and sedatives					
Riewpaiboon et al 2014	Paracetamol 120 mg/5 mL in 60-mL bottle	per bottle	2010	0.33	750.38
Riewpaiboon et al 2014	Paracetamol 500 mg tablet	Per tablet	2010	0.01	26.80
Antifungal and antibacterial drugs					
Riewpaiboon et al 2014	Ampicillin 500 mg vial injection	Per piece	2010	0.21	482.39
Riewpaiboon et al 2014	Ceftriaxone 250 mg vial injection	Per piece	2010	0.46	1,045.17
Riewpaiboon et al 2014	Ciprofloxacin 250 mg capsule	Per Capsule	2010	0.01	26.80
Riewpaiboon et al 2014	Ciprofloxacin 500 mg capsule	Per capsule	2010	0.05	107.20
Penno et al 2015	Antimicrobial treatment using generic drugs for sepsis management	per case	2011	15.17	34,703.66
Penno et al 2015	Evidence-based antimicrobials	Per case	2011	30.36	69,459.83
Kimaro et al 2017	Cotrimoxazole per daily dose	Per daily dose	2012	0.02	51.51

For peer review only - http://bmjopen.bmj.com/site/about/guidelines.xhtml

Others like anti-cancers					
Githang'a et al 2020	50 mg of 6-mercaptopurine (6MP)	Unclear	2013	0.39	885.94
Mengistu et al 2019	Drugs for emergency obstetric care	per patient	2013	6.90	15,795.12
Riewpaiboon et al 2014	Glucose (5%) 500 mL	Per unit	2010	0.50	1,152.37
Guerriero et al 2010	Tranexamic acid	Per dose	2007	9.74	22,279.66

When looking at cost categories in table 4, two studies reported costs of equipment, two studies reported costs of consumables and six studies reported medication cost. Within studies, we found per item cost for equipment (4 items), consumables (3 items) and medication (13 items). The cost of a mannequin used in training was the most expensive piece of equipment reported (USD 76.03)[50]. In the medication category, tranexamic acid was the most expensive per dose (USD 9.74)[51], whereas antimicrobials were the most expensive on a per case basis (USD 30.36)[52].

Cost of diagnostics

Six studies reported on the cost of diagnostics, for example, full blood count (FBC), serum cryptococcal meningitis test, sputum culture and imaging. Lab assessment for sepsis- ID and susceptibilities positive blood culture was the most expensive diagnostic test (USD 82.55) followed by CT scans (USD 47.81 to 44.35) (see supplementary 6 for details).

Costs of human resources

Three studies reported human resource costs (see supplementary 7). However, the breakdown of human resource costs presented in the papers did not allow for a separation of costs between treatment for the condition and the critical illness care. Reporting of human resource cost was heterogenous. One study provided monthly salaries for all personnel with monthly salaries ranging from USD 2721.79 for medical specialists USD 313.34 for nurses[53]. Another study reported total cost of personnel as a line item[54]. The third study reported unit costs (i.e. cost per patient) treatment for all obstetric complications broken down by staff category[55]. This study

For peer review only - http://bmjopen.bmj.com/site/about/guidelines.xhtml

found that the highest cost was for an enrolled nurse or midwife (USD 43.62 per patient) and that the lowest unit personnel cost was for an anaesthesiologist (less than 0.01USD per patient)[55].

Discussion

Our review found limited evidence on resource use, availability and costs of critical care in Tanzania. Of the eligible evidence, we found high variability in the geographical distribution and availability of resources needed for both ICU and non-ICU critical care provision. We found that ICU critical care was provided at the tertiary level in urban areas and no published provision in rural areas, whereas, non-ICU critical care was evident in both rural and urban areas. Within hospitals, we find that availability of key critical care resources (e.g. pulse oximeters, oxygen cylinders, mask and tubing) can be limited but the use of these resources is not documented. We, finally, find no reported estimates on the unit costs of critical care; and that the generation and reporting of cost data is not standardised thereby limiting synthesis or aggregation.

Availability of critical care in Tanzania

The majority of ICUs identified in our systematic review were located in tertiary referral hospitals and private hospitals located in urban areas (Dar es Salaam, Moshi, Mwanza, Mbeya city). This is of particular concern in Tanzania where the majority of the population (64.77) lives in rural areas[56]. Although this inequity in service distribution is not unique to Tanzania as previous studies in LMICs found all hospitals with ICUs were located in cities [37] , it is further

For peer review only - http://bmjopen.bmj.com/site/about/guidelines.xhtml

exacerbated by a lack of critical care guidelines that cater to rural areas[57]. Such geographical inequity in ICU critical care could be detrimental and of particular concern during a critical care crisis (e.g. COVID-19)[1].

However, critical care delivery is heterogenous and can be provided in advanced or basic forms, depending on resource availability. Critical care can involve high tech and high-cost equipment that requires highly trained staff (e.g. mechanical ventilation) and is more often found in the ICU. Investing in this level of critical care is therefore an expensive policy decision and may explain the lack of ICU critical care availability in rural areas in Tanzania. On the other hand, investing in adequately resourced critical care outside of ICUs can potentially save lives and reduce the case load that requires ICU care [58]. We found that non-ICU critical care was reported to be given in wards and theatres[43] across rural and urban areas.

A key finding of the literature review was that studies did not provide breakdowns of costs by the level of care provided, complexity of care, resource intensity or quality of provision . Importantly, for our study, they also did not provide breakdowns between the care for a specific condition relative to the care for the critical illness. We were therefore only able to characterise critical care in Tanzania by where the care was provided within a hospital i.e. ICU vs non-ICU delivered critical care. This is of importance as care for the critically ill takes many forms/interventions depending not only on whether it is ICU based but also by the complexity, the type of provider, the quality of care as well as the primary diagnosis. Characterising the minimum needs for the care of a critically ill patient and understanding how critical care delivery varies across these parameters will facilitate our understanding of what elements of critical care

For peer review only - http://bmjopen.bmj.com/site/about/guidelines.xhtml

are currently available as well as recommendations for the progressive improvement of critical care in all facilities. Previous research suggests that basic critical care, which effectively identifies and treats all critically ill patients, should be available and provided in all settings within a hospital (e.g. in wards) across rural and urban settings [59,60].

Recently, some of this paper's authors have proposed the concept of Essential Emergency and Critical Care (EECC) across LMICs which could be the future of low-cost critical care. EECC is pragmatic and low-cost care of critical illness that does not need to be reliant on sophisticated technology, and have a primary focus on simple, effective actions that have large potential impact on the population[58] and the components of have been specified in a large global consensus[12]. Some of the critical care interventions that have been identified as part of EECC[12] that can be provided in general wards include; identification of critical illness using vital signs based triage, caring for blocked or threatened airway for example suction for secretions that are obstructing the airway and insertion of an oropharyngeal airway, caring for hypoxia or respiratory distress, threatened circulation or shock and reduced level of consciousness, see Schell et al[12] for further details.

Given the lack of availability and access of critical care across Tanzania, such novel approaches may provide a building block to developing a fully resourced, equitable critical care system[61]. However, decision makers require further evidence on the optimal design, costs, budget impact and cost-effectiveness of such critical care interventions in rural and urban systems.

Complexity of resources within critical care

For peer review only - http://bmjopen.bmj.com/site/about/guidelines.xhtml

At the hospital level, we found that many studies identified resources that were used in the provision of services that included a critical care component but only a few studies went further to report on the availability of the resources at the facility (e.g. equipment, consumables and human resources). Of the critical care components, resource availability of oxygen was explored in four studies[38,41,43,62]. Other components explored were personal protective equipment (two studies)[38,41] and availability of utilities like running water and electricity (two studies)[38,41].

While oxygen is one of the essential resources required in the provision of critical care, many hospitals still lack basic equipment for its storage and delivery[63]. Our findings show that even when oxygen is available, the equipment and consumables required to facilitate provision of oxygen therapy can be missing[43,62]. This leaves a considerable proportion of critically ill patients in need of medical oxygen without the necessary treatment, incurring unnecessary deaths and disability.

Many reasons exist as to why availability and supply of oxygen is limited in settings such as Tanzania. For example, supply and scaling of medical oxygen is a complex process and is often controlled though monopoly suppliers. Regulation and high investment costs create barriers for new firms to enter the market and easily scale up supply[64]. In Tanzania, medical oxygen supply is controlled centrally by a quasi-governmental monopoly organisation[65]. On the demand side, there is limited funding for medical oxygen with procurement decentralised to individual hospitals with constraint budgets[63]. Alternatives, such as oxygen concentrators and hospital plants for the local production of oxygen, are affected by the need for a constant supply of

For peer review only - http://bmjopen.bmj.com/site/about/guidelines.xhtml

electricity, compounded by challenges with maintaining equipment. In Tanzania, similar to many LMICs, approx. 50% of hospitals are without a constant oxygen supply[41] and there is limited local capacity to maintain and repair oxygen concentrators like Tanzania.

In addition to oxygen equipment, we found variability in availability of personal protective equipment (PPE), and human resources across studies. Some PPE especially gloves, were available in most hospitals, while others, such as eye protection, remained unavailable, which is in line with previous research[66]. In terms of human resources, evidence suggests that only 20% of hospitals have at least one health worker trained in adult critical care which includes administering complex oxygen related equipment [38,57,59]. Technical assistance, training of health workers, and improved processes for non ICU based care, development of context-specific critical care guidelines, building capacity in equipment maintenance and biomedical engineering in facilities, and support to medical oxygen infrastructure development should be prioritised by governments and in funding applications to multilaterals and technical assistance agencies [38,67,68].

Costs of critical care

Costing of critical care is vital in eliciting potential budget constraints or impacts, assessing cost-effectiveness, providing insight into the economic burden of disease and can be useful for understanding the resources incurred by health systems, other payers, and patients. We find that no study reported or calculated an average cost or cost per patient day for the provision of critical services. On the other hand, many studies reported individual costs of resources associated with provision of critical care and it was possible to identify the cost of specific resources needed in

For peer review only - http://bmjopen.bmj.com/site/about/guidelines.xhtml

critical care provision (i.e. human resource cost, services, medication, and other consumables). Such limited evidence could be due to no formal or standardised definition of critical care existing thereby reducing specificity when costing critical care provisioning. Another possibility may be that patients who require critical care are heterogenous. The underlying demographics, epidemiology, risk factors, patient pathway and interventions administered are also heterogenous thereby making aggregation and estimates of average cost challenging. This is further confirmed by the data extracted in this review which showed that reporting of costs within eligible studies varied by defined services provided, breakdowns of costs and inputs, as well as the cost unit, thereby making comparisons or synthesising information across studies difficult. There is an urgent need to understand the resources required and their cost implications to inform the planning of improved critical care, globally.

Almost 50% of the Tanzanian population have a daily consumption of less than 1.91USD[69]. At the same time, the share of out-of-pocket payments in total health expenditure was 22% in 2019[70]. Considering the cost of high-tech critical care inputs, it is likely that critical care exposures patients to considerable out of pocket expenses resulting in catastrophic expenditure for low-income households that seek hospital care for critically ill family members. Countries should consider implementing EECC which could reduce the likely cost of critical care and therefore the financial burden on patients' households in their respective settings.

Lessons learned for other low resource settings/LMICs

The variability in critical care provision, availability of critical care resources, prices and costs are not unique to Tanzania. The heterogeneity in critical care is well documented across the

For peer review only - http://bmjopen.bmj.com/site/about/guidelines.xhtml

globe[71]. Variability in costs is also well documented across health facilities in LMICs[5]. The research and data gaps in these areas– understanding resource availability in critical care across different settings (both ICU and non ICU) - is less well documented but it is likely that the Tanzania's data gaps are replicated in many LMICs. LMICs will require research and data collection efforts in ascertaining these resource gaps and costing of critical care to inform budgets and planning.

Limitations

There are a number of inherent limitations with this systematic review. Firstly, we tailored our search strategy to account for the lack of standardised or formal definitions for critical illness, critical care provision and ICUs [59] by including specific terms for conditions likely to lead to critical illness and identified in consultation with clinicians on the authorship team. Here, we may have introduced a personal bias by focussing on specific conditions and excluding others. Secondly, cost and resource use in an African context are traditionally poorly documented and can fluctuate due to market dynamics and resource availability. While this systematic review provides key insights into peer reviewed published information on the resource use, availability and costs of critical care in Tanzania, researchers must update a previous nationally representative facility survey conducted in 2012 extending it to include an understanding of the costs of critical care provision that includes patient level data on resource use. Finally, we opted to only include articles in English but believe the extent and effects of language bias are diminished recently because of the shift towards publication of studies in English[72].

For peer review only - http://bmjopen.bmj.com/site/about/guidelines.xhtml

Conclusion

Critical care is essential in the prevention of mortality among critically ill patients, yet limited evidence exists on the costs and cost-effectiveness of scaling up critical care thereby hindering informed planning for investments in key life-saving interventions. We further find that ICU based critical care provision is currently only provided in urban areas in Tanzania and there is an urgent need to assess the quality of non-ICU based critical care implemented to address the gap in critical care provision in rural and urban areas. Evidence on resources used and potential costs in critical care is limited, and, along with a specific focus on oxygen supply and delivery which is a key resource in critical care, requires further research. Improving the evidence base on the costs, utilisation, cost-effectiveness and equity impacts of different strategies for critical care services across rural and urban areas is needed to help prevent unnecessary deaths caused by critical illness in Tanzania.

Authors' contributions

Below is the detailed breakdown of their contribution.

Concept and design: JK, HS, LG, AK, PT, OS, TB

Development of first draft: JK, HS, LG

Acquisition of data: JK, HS, LG, PT, AK, KK, SG

Analysis and interpretation of data: JK, HS, LG, PT, SG, OS, KK, TB

Drafting of the manuscript: JK, HS, LG

Critical revision of paper for important intellectual content: TB, OS, LG, PT, SG, KK, AK

Statistical analysis: -

For peer review only - http://bmjopen.bmj.com/site/about/guidelines.xhtml

Provision of study materials:

Obtaining funding: TB, OS

Administrative and technical support:

Supervision: LG, HS

Competing interests

Authors declare no competing interests and the funders did not in any way influence the development of the development of this study.

TB declares personal fees from UNICEF, the World Bank, USAID and the Wellcome Trust, all outside the submitted work.

Funding statement

This work was supported by the Wellcome Trust [221571/Z/20/Z], as part of the 'Innovation in low-and middle-income countries' Flagship.

Data sharing statement

Supporting data is available on request.

Figures

Figure 1: PRISMA Chart

Legend: The PRISMA diagram shows the details the search and selection process applied during our systematic literature search and review.

References

1. Baker T, Otto Schell C, Brun Petersen D, et al. Essential care of critical illness must not be forgotten in the COVID-19 pandemic. *Lancet*. 2020;395:1253-1254.

For peer review only - http://bmjopen.bmj.com/site/about/guidelines.xhtml

doi:10.1016/S0140-6736(20)30793-5

2. Barasa E, Kairu A, Nganga W, et al. Examining Unit Costs for COVID-19 Case Management in Kenya. *medRxiv*. Published online October 13, 2020:2020.10.08.20209684. doi:10.1101/2020.10.08.20209684

3. Sen-Crowe B, Sutherland M, McKenney M, Elkbuli A. A Closer Look Into Global Hospital Beds Capacity and Resource Shortages During the COVID-19 Pandemic. *J Surg Res*. 2021;260:56. doi:10.1016/J.JSS.2020.11.062

4. Adhikari NKJ, Fowler RA, Bhagwanjee S, Rubenfeld GD. Critical care and the global burden of critical illness in adults. *Lancet*. 2010;376(9749):1339-1346. doi:10.1016/S0140-6736(10)60446-1

5. Reynolds TA, Sawe H, Rubiano AM, Shin S Do, Wallis L, Mock CN. Strengthening Health Systems to Provide Emergency Care. In: Jamison DT, Gelband H, Horton S, et al., eds. *Disease Control Priorities, Third Edition (Volume 9): Improving Health and Reducing Poverty*. 3rd ed. The International Bank for Reconstruction and Development / The World Bank; 2017:247-265. doi:10.1596/978-1-4648-0527-1_CH13

6. Ayebale ET, Kassebaum NJ, Roche AM, Biccard BM. Africa's critical care capacity before COVID-19. *South African J Anaesth Analg*. 2020;26(3):162-164. doi:10.36303/SAJAA.2020.26.3.2431

7. Kayambankadzanja RK, Schell CO, Wärnberg MG, et al. Towards definitions of critical illness and critical care using concept analysis. *medRxiv*. Published online January 10, 2022:2022.01.09.22268917. doi:10.1101/2022.01.09.22268917

For peer review only - http://bmjopen.bmj.com/site/about/guidelines.xhtml

8. National Institute for Health and Care Excellence (NICE). What “critical care” means | Information for the public | Rehabilitation after critical illness in adults | Guidance | NICE. Published March 25, 2009. Accessed November 1, 2020. https://www.nice.org.uk/guidance/cg83/ifp/chapter/What-critical-care-means

9. Zimmerman JE, Kramer AA, Knaus WA. Changes in hospital mortality for United States intensive care unit admissions from 1988 to 2012. *Crit Care*. 2013;17(2):R81. doi:10.1186/cc12695

10. Turner HC, Van Hao N, Yacoub S, et al. Achieving affordable critical care in low-income and middle-income countries Commentary Handling editor Seye Abimbola. *BMJ Glob Heal*. 2019;4:1675. doi:10.1136/bmjgh-2019-001675

11. Schell CO, Gerdin Wärnberg M, Hvarfner A, et al. The global need for essential emergency and critical care. *Crit Care*. 2018;22(1):1-5. doi:10.1186/s13054-018-2219-2

12. Schell CO, Khalid K, Wharton-Smith A, et al. Essential Emergency and Critical Care: a consensus among global clinical experts. *BMJ Glob Heal*. 2021;6(9):e006585. doi:10.1136/BMJGH-2021-006585

13. Kayambankadzanja RK, Schell CO, Namboya F, et al. The prevalence and outcomes of sepsis in adult patients in two hospitals in Malawi. *Am J Trop Med Hyg*. 2020;102(4):896-901. doi:10.4269/ajtmh.19-0320

14. Kayambankadzanja RK, Schell CO, Mbingwani I, Mndolo SK, Castegren M, Baker T. Unmet need of essential treatments for critical illness in Malawi. Chen T-H, ed. *PLoS One*. 2021;16(9):e0256361. doi:10.1371/JOURNAL.PONE.0256361

For peer review only - http://bmjopen.bmj.com/site/about/guidelines.xhtml

15. King C, Banda M, Bar-Zeev N, et al. Care-seeking patterns amongst suspected paediatric pneumonia deaths in rural Malawi. *Gates Open Res 2021 4178*. 2021;4:178. doi:10.12688/gatesopenres.13208.2

16. Hillary C, Tom P, P. K, A.M. M, G. S, D. M. A survey of emergency and surgical services in the United Republic of Tanzania. *Acad Emerg Med*. 2011;18(5 SUPPL. 1):S242. doi:http://dx.doi.org/10.1111/j.1553-2712.2011.01073.x

17. World Health Organisation. *Strengthening the Health Systems Response to COVID-19: Creating Surge Capacity for Acute and Intensive Care* .; 2020. Accessed September 21, 2021. http://apps.who.int/bookorders.

18. World Bank. World Bank facilitates immediate support and resources for the COVID-19 (Coronavirus) emergency in Bolivia. Published April 2, 2020. Accessed December 16, 2020. https://www.worldbank.org/en/news/press-release/2020/04/02/el-banco-mundial-facilita-apoyo-y-recursos-inmediatos-para-atender-la-emergencia-por-el-covid-19-coronavirus-en-bolivia

19. The World Bank. *The World Bank Annual Report 2020: Supporting Countries in Unprecedented Time*.; 2020. Accessed December 16, 2020. https://www.worldbank.org/en/about/annual-report/covid-response

20. Kazibwe J, Shah HA, Kuwawenaruwa A, et al. Resource availability, utilisation and cost in the provision of critical care in Tanzania: a protocol for a systematic review. *BMJ Open*. 2021;11(8):e050881. doi:10.1136/BMJOPEN-2021-050881

21. Page MJ, McKenzie JE, Bossuyt PM, et al. The PRISMA 2020 statement: an updated guideline for reporting systematic reviews. *BMJ*. 2021;372. doi:10.1136/BMJ.N71

For peer review only - http://bmjopen.bmj.com/site/about/guidelines.xhtml

22. Moher D, Liberati A, Tetzlaff J, et al. Preferred reporting items for systematic reviews and meta-analyses: The PRISMA statement (Chinese edition). *J Chinese Integr Med*. 2009;7(9):889-896. doi:10.3736/jcim20090918

23. Savilaakso S, Garcia C, Garcia-Ulloa J, et al. Systematic review of effects on biodiversity from oil palm production. *Environ Evid*. 2014;3(1):4. doi:10.1186/2047-2382-3-4

24. Abbafati C, Machado DB, Cislaghi B, et al. Global burden of 369 diseases and injuries in 204 countries and territories, 1990–2019: a systematic analysis for the Global Burden of Disease Study 2019. *Lancet*. 2020;396(10258):1204-1222. doi:10.1016/S0140-6736(20)30925-9

25. Kazibwe J, Shah HA, Kuwawenaruwa A, et al. Resource availability, utilization and cost in the provision of critical care in Tanzania: A protocol for a systematic review. *BMJ Open*. Published online 2021.

26. Ouzzani M, Hammady H, Fedorowicz Z, Elmagarmid A. Rayyan-a web and mobile app for systematic reviews. *Syst Rev*. 2016;5(1). doi:10.1186/s13643-016-0384-4

27. World bank. GDP deflator (base year varies by country) - Tanzania . Accessed June 2, 2021. https://data.worldbank.org/indicator/NY.GDP.DEFL.ZS?locations=TZ

28. Vassall A, Sweeney S, Kahn JG, et al. *Reference Case for Estimating the Costs of Global Health Services and Interventions*.

29. Adawiyah R al, Saweri OPM, Boettiger DC, et al. The costs of scaling up HIV and syphilis testing in low- and middle-income countries: a systematic review. *Health Policy Plan*. 2021;2021:1-16. doi:10.1093/heapol/czab030

For peer review only - http://bmjopen.bmj.com/site/about/guidelines.xhtml

30. Nicks BA, Sawe HR, Juma AM, Reynolds TA. The state of emergency medicine in the United Republic of Tanzania. *African J Emerg Med*. 2012;2(3):97-102. doi:10.1016/j.afjem.2012.06.002

31. Sylvanus E, Sawe HR, Muhanuzi B, et al. Profile and outcome of patients with emergency complications of renal failure presenting to an urban emergency department of a tertiary hospital in Tanzania. *BMC Emerg Med*. 2019;19(1):11. doi:http://dx.doi.org/10.1186/s12873-019-0229-2

32. Engdahl Mtango S, Lugazia E, Baker U, Johansson Y, Baker T. Referral and admission to intensive care: A qualitative study of doctors' practices in a Tanzanian university hospital. Brenner S, ed. *PLoS One*. 2019;14(10):e0224355. doi:10.1371/journal.pone.0224355

33. Mkoka DA, Goicolea I, Kiwara A, Mwangu M, Hurtig AK. Availability of drugs and medical supplies for emergency obstetric care: Experience of health facility managers in a rural District of Tanzania. *BMC Pregnancy Childbirth*. 2014;14(1):1-10. doi:10.1186/1471-2393-14-108

34. Papali A, Adhikari NKJ, Diaz J V., et al. Infrastructure and organization of adult intensive care units in resource-limited settings. In: *Sepsis Management in Resource-Limited Settings*. Springer International Publishing; 2019:31-68. doi:10.1007/978-3-030-03143-5_3

35. Mwiga F, Monaghan M. Development of a difficulty airway trolley (DAT) at a tertiary referral hospital in northern Tanzania. *Trends Anaesth Crit Care*. 2020;30:e107-e108. doi:http://dx.doi.org/10.1016/j.tacc.2019.12.265

36. Sawe HR, Reynolds TA, Mfinanga JA, et al. The clinical presentation, utilization, and

For peer review only - http://bmjopen.bmj.com/site/about/guidelines.xhtml

outcome of individuals with sickle cell anaemia presenting to urban emergency department of a tertiary hospital in Tanzania. *BMC Hematol*. 2018;18(1):25. doi:http://dx.doi.org/10.1186/s12878-018-0122-3

37. Murthy S, Leligdowicz A, Adhikari NKJ. Intensive care unit capacity in low-income countries: A systematic review. *PLoS One*. 2015;10(1):116949. doi:http://dx.doi.org/10.1371/journal.pone.0116949

38. Baker T, Lugazia E, Eriksen J, Mwafongo V, Irestedt L, Konrad D. Emergency and critical care services in Tanzania: A survey of ten hospitals. *BMC Health Serv Res*. 2013;13(1):1-9. doi:10.1186/1472-6963-13-140

39. Rajaguru PP, Jusabani MA, Massawe H, Temu R, Sheth NP. Understanding surgical care delivery in Sub-Saharan Africa: a cross-sectional analysis of surgical volume, operations, and financing at a tertiary referral hospital in rural Tanzania. *Glob Heal Res policy*. 2019;4:30. doi:https://dx.doi.org/10.1186/s41256-019-0122-2

40. Reynolds TA, Mfinanga JA, Sawe HR, Runyon MR, Mwafongo V. Emergency care capacity in Africa: a clinical and educational initiative in Tanzania. *J Public Health Policy*. 2012;33(Supplement 1):S126-S137. doi:http://dx.doi.org/10.1057/jphp.2012.41

41. Penoyar T, Cohen H, Kibatala P, et al. Emergency and surgery services of primary hospitals in the United Republic of Tanzania. *BMJ Open*. 2012;2(1):e000369. doi:10.1136/bmjopen-2011-000369

42. Chalya PL, Gilyoma JM, Dass RM, et al. Trauma admissions to the Intensive care unit at a reference hospital in Northwestern Tanzania. *Scand J Trauma Resusc Emerg Med*. 2011;19:61. doi:10.1186/1757-7241-19-61

For peer review only

For peer review only - http://bmjopen.bmj.com/site/about/guidelines.xhtml

43. Staton CA, Msilanga D, Kiwango G, et al. A prospective registry evaluating the epidemiology and clinical care of traumatic brain injury patients presenting to a regional referral hospital in Moshi, Tanzania: challenges and the way forward. *Int J Inj Contr Saf Promot*. 2017;24(1):69-77. doi:10.1080/17457300.2015.1061562

44. Sawe HR, Reynolds TA, Mfinanga JA, et al. The clinical presentation, resource utilization, and outcomes of patients with sickle cell disease presenting to the ED of the muhimbili national Hospital in Dar es Salaam Tanzania. *Acad Emerg Med*. 2016;23(SUPPL. 1):S99. doi:http://dx.doi.org/10.1111/acem.12974

45. Rajaguru PP, Jusabani MA, Massawe H, Temu R, Sheth NP. Understanding surgical care delivery in Sub-Saharan Africa: a cross-sectional analysis of surgical volume, operations, and financing at a tertiary referral hospital in rural Tanzania. *Glob Heal Res Policy*. 2019;4(1):30. doi:10.1186/s41256-019-0122-2

46. Kimaro GD, Mfinanga S, Simms V, et al. The costs of providing antiretroviral therapy services to HIV-infected individuals presenting with advanced HIV disease at public health centres in Dar es Salaam, Tanzania: Findings from a randomised trial evaluating different health care strategies. *PLoS One*. 2017;12(2):e0171917. doi:http://dx.doi.org/10.1371/journal.pone.0171917

47. Sicuri E, Vieta A, Lindner L, Constenla D, Sauboin C. Economic costs of malaria in children in three Sub-Saharancountries: Ghana, Tanzania and Kenya. *Trop Med Int Heal*. 2011;16(SUPPL. 1):117. http://ovidsp.ovid.com/ovidweb.cgi?T=JS&PAGE=reference&D=emed12&NEWS=N&AN=70589204

For peer review only - http://bmjopen.bmj.com/site/about/guidelines.xhtml

48. Riewpaiboon A, Piatti M, Ley B, et al. Cost of illness due to typhoid fever in Pemba, Zanzibar, East Africa. *J Heal Popul Nutr*. 2014;32(3):377-385. http://www.jhpn.net/index.php/jhpn/article/view/2819/1043

49. James C, Bura M, Ensor T. The costs of delivering health services in Tanzania. Findings from a comprehensive costing analysis.

50. Chaudhury S, Arlington L, Brenan S, et al. Cost analysis of large-scale implementation of the "Helping Babies Breathe" newborn resuscitation-training program in Tanzania. *BMC Health Serv Res*. 2016;16(1):681. http://ovidsp.ovid.com/ovidweb.cgi?T=JS&PAGE=reference&D=emed17&NEWS=N&AN=618644170

51. Guerriero C, Cairns J, Jayaraman S, Roberts I, Perel P, Shakur H. Giving tranexamic acid to reduce surgical bleeding in sub-Saharan Africa: An economic evaluation. *Cost Eff Resour Alloc*. 2010;8:1. doi:http://dx.doi.org/10.1186/1478-7547-8-1

52. Penno EC, Baird SJ, Crump JA. Cost effectiveness of surveillance for bloodstream infections for sepsis management in low resource settings. *Am J Trop Med Hyg*. 2015;93(4):850-860. http://www.ajtmh.org/content/91/5_Suppl_1/74.full.pdf+html

53. Shayo GA, Chitama D, Moshiro C, Aboud S, Bakari M, Mugusi F. Cost-Effectiveness of isoniazid preventive therapy among HIV-infected patients clinicaly screened for latent tuberculosis infection in Dar es Salaam, Tanzania: A prospective Cohort study. *BMC Public Health*. 2017;18(1):35. doi:http://dx.doi.org/10.1186/s12889-017-4597-9

54. Mengistu T, Berruti A, Krivelyova A, Swor M, Waite R, Maro G. Cost of providing emergency obstetric care in Tanzania's Kigoma region. *Int J Health Plann Manage*.

For peer review only - http://bmjopen.bmj.com/site/about/guidelines.xhtml

2019;34(4):e1510-e1519. doi:http://dx.doi.org/10.1002/hpm.2820

55. Baynes C, Yegon E, Kimaro G, Lusiola G, Kahwa J. The unit and scale-up cost of postabortion care in Tanzania. *Spec Issue Sav women's lives through Emerg Obstet care Volunt Fam planning*. 2019;7(Suppl. 2):S327-S341. doi:http://dx.doi.org/10.9745/GHSP-D-19-00035

56. The World Bank Group. Rural population (% of total population) - Tanzania | Data. 2021. Accessed July 14, 2021. https://data.worldbank.org/indicator/SP.RUR.TOTL.ZS?locations=TZ

57. Schultz MJ, Dunser MW, Dondorp AM, et al. Current challenges in the management of sepsis in ICUs in resource-poor settings and suggestions for the future. *Intensive Care Med*. 2017;43:612-624. doi:10.1007/s00134-017-4750-z

58. Ranjit S, Kissoon N. Challenges and Solutions in translating sepsis guidelines into practice in resource-limited settings. *Transl Paediatr*. Published online 2021. Accessed August 10, 2021. https://tp.amegroups.com/article/viewFile/66320/pdf

59. Baker T, Khalid K, Acicbe O, McGloughlin S, Amin P. Critical care of tropical disease in low income countries: Report from the Task Force on Tropical Diseases by the World Federation of Societies of Intensive and Critical Care Medicine. *J Crit Care*. 2017;42:351-354. doi:10.1016/J.JCRC.2017.11.028

60. Bion J, Brown C, Gomersall C, Boulanger C, Isherwood P, Schulman D. Society of Critical Care Medicine 50th Anniversary Review Series: Critical Care Education. *Crit Care Med*. Published online 2021:1241-1253. doi:10.1097/CCM.0000000000005130

For peer review only - http://bmjopen.bmj.com/site/about/guidelines.xhtml

61. Kairu A, Were V, Isaaka L, Agweyu A, Aketch S, Barasa E. Modelling the cost-effectiveness of essential and advanced critical care for COVID-19 patients in Kenya. *medRxiv*. Published online August 24, 2021:2021.08.16.21261894. doi:10.1101/2021.08.16.21261894

62. Zimmerman A, Fox S, Griffin R, et al. An analysis of emergency care delays experienced by traumatic brain injury patients presenting to a regional referral hospital in a low-income country. *PLoS One*. 2020;15(10 October 2020):e0240528. doi:http://dx.doi.org/10.1371/journal.pone.0240528

63. Kazibwe Joseph, Baker Peter, Baker Tim, et al. We Can Stop Preventable COVID Deaths by Urgently Prioritising Medical Oxygen and Essential Critical Care | Center For Global Development. Center for Global Development. Published February 9, 2021. Accessed August 2, 2021. https://www.cgdev.org/blog/we-can-stop-preventable-covid-deaths-urgently-prioritising-medical-oxygen-and-essential

64. Davies M, Onwuzoo A, Mednick S. Fighting for breath: how the medical oxygen industry is failing African hospitals . The Guardian. Published August 10, 2020. Accessed August 9, 2022. https://www.theguardian.com/global-development/2020/aug/10/fighting-for-breath-how-the-medical-oxygen-industry-is-failing-african-hospitals

65. Tanzania Medicines and Medical Devices Authority. Guidelines for regulating medical gases. Accessed September 16, 2021. https://www.tmda.go.tz/uploads/publications/en1597391138-GUIDELINES FOR MEDICAL GASES - Final (1)-SK.pdf

66. Powell-Jackson T, King JJC, Makungu C, et al. Infection prevention and control

For peer review only - http://bmjopen.bmj.com/site/about/guidelines.xhtml

compliance in Tanzanian outpatient facilities: a cross-sectional study with implications for the control of COVID-19. *Lancet Glob Heal*. 2020;8(6):e780-e789. doi:10.1016/S2214-109X(20)30222-9

67. Murthy S, Adhikari NK. ATS REPORTS Global Health Care of the Critically Ill in Low-Resource Settings. *Ann Am Thorac Soc*. 2013;10(5):509-513. doi:10.1513/AnnalsATS.201307-246OT

68. Diaz J V., Riviello ED, Papali A, Adhikari NKJ, Ferreira JC. Global critical care: Moving forward in resource-limited settings. *Ann Glob Heal*. 2019;85(1):3-4. doi:10.5334/aogh.2413

69. World Bank Group. Poverty & Equity Brief Sub Saharan – Tanzania. Published April 2020. Accessed August 18, 2022. https://databankfiles.worldbank.org/data/download/poverty/33EF03BB-9722-4AE2-ABC7-AA2972D68AFE/Global_POVEQ_TZA.pdf

70. The World Bank Group. Out-of-pocket expenditure (% of current health expenditure) - Tanzania. Accessed July 26, 2022. https://data.worldbank.org/indicator/SH.XPD.OOPC.CH.ZS?locations=TZ

71. Wilcox ME, Vaughan K, Chong CAKY, Neumann PJ, Bell CM. Cost-Effectiveness Studies in the ICU: A Systematic Review∗. *Crit Care Med*. 2019;47(8):1011-1017. doi:10.1097/CCM.0000000000003768

72. Cochrane Reviews. Cochrane Handbook: 10.2.2.4 Language bias. In: *Cochrane*. Accessed September 16, 2021. https://handbook-5-1.cochrane.org/chapter_10/10_2_2_4_language_bias.htm

For peer review only - http://bmjopen.bmj.com/site/about/guidelines.xhtml

For peer review only

For peer review only - http://bmjopen.bmj.com/site/about/guidelines.xhtml

Figures

Figure 1: PRISMA Chart

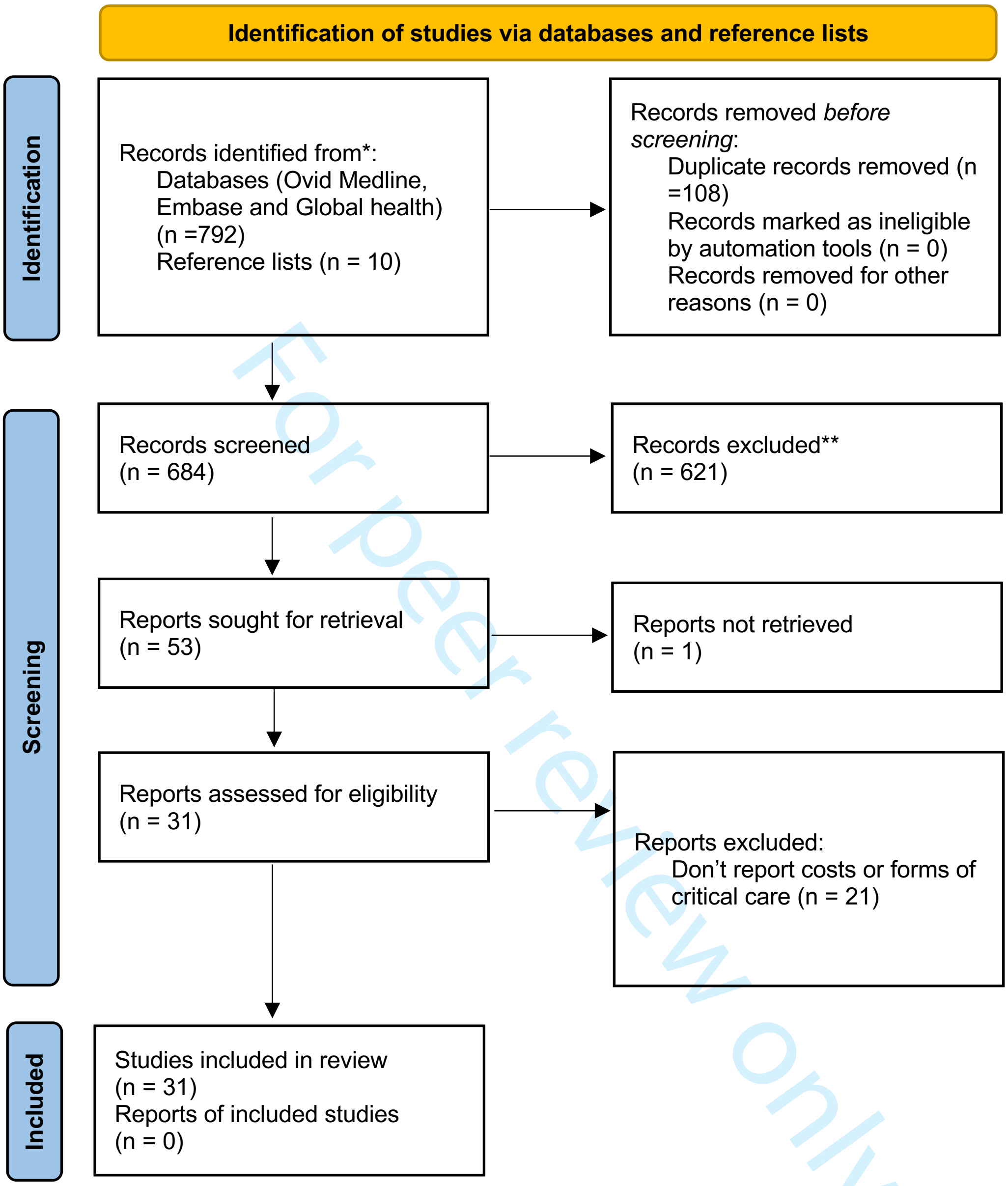

Legend: The PRISMA diagram shows the details the search and selection process applied during our systematic literature search and review.

Based on Page MJ, McKenzie JE, Bossuyt PM, Boutron I, Hoffmann TC, Mulrow CD, et al. The PRISMA 2020 statement: an updated guideline for reporting systematic reviews. BMJ 2021;372:n71. doi: 10.1136/bmj.n71

For peer review only - http://bmjopen.bmj.com/site/about/guidelines.xhtml

SUPPLEMENTARY MATERIAL

Supplementary 1: Key concepts and search terms identified for the search.

Search platform: Ovid

Database: Embase Classic+Embase <1947 to 2020 November 18> , Global Health <1973 to 2020 Week 45>, Ovid MEDLINE(R) ALL <1946 to November 17, 2020>
Search Strategy:
----
1 exp intensive care/ or hospital care.ab,ti. or artificial ventilation/ or early goal-directed therapy/ or intensive care nursing/ or newborn intensive care/ or patient monitoring/ or pediatric advanced life support.ab,ti. or emergency.ab,ti. or urgent care.ab,ti. (1538827)
2 (hospital patient or inpatient).ab,ti. (248658)
3 exp hospital patient/ (188072)
4 exp "health care cost"/ or "cost"/ or health economics/ or "drug cost"/ or health care financing/ or "hospital cost"/ or nursing cost.ab,ti. or cost.ab,ti. or costs.ab,ti. (1697788)
5 exp tanzania/ or tanzania.ab,ti. (45585)
6 exp Health Care Costs/ or exp Health Expenditures/ or (cost* or ((health care or health-care or healthcare or medical) adj3 expenditure*) or expense* or hospital resources).ti,ab. or (((resource* or "health service") adj3 ("use" or utilisation or utilization)) or ((financial or economic) and (burden or impact or consequence)) or spending or out of pocket or out-of-pocket or OOP or insurance or absenteeism or (productivity and (loss or lost)) or "economic modelling").ab,ti. (2248416)
7 artificial ventilation.ab,ti. (7728)
8 (malaria or tb or tuberculosis or diagnostics or laboratory or lab or hiv).ab,ti. (3253274)
9 (general care or general ward care or general inpatient care or hospital care).ab,ti. (27507)
10 1 or 2 or 3 or 7 or 8 or 9 (4988953)
11 (economic evaluation or cost effective).ab,ti. (264378)
12 4 or 6 or 11 (2320354)
13 5 and 10 and 12 (1905)
14 limit 13 to yr="2010 -Current" (1295)
15 remove duplicates from 14 (792)

For peer review only

Supplementary 2: Inclusion and exclusion criteria

Inclusion criterion

For a study to be included it should fulfil the following criterion

- Published in English in a peer reviewed journal
- Reports forms or types of critical care offered, critical care services offered and or costs and resources used in the provision of care in Tanzania
- Includes costs from a provider perspective
- Articles published from 2010

Exclusion criterion

Studies will be excluded in case of the following;

- Published in any other languages except English
- Studies carried out outside Tanzania
- Studies with costs estimated from patient perspective
- Studies without full text versions
- Studies published before 2010

Supplementary 3: Study Characteristics (N=31)]

Article	Disease area	Intervention	Study Type	Purpose Type	Geographical area covered	Hospital Type	Facility/hospital Level	Source of data
**Studies reporting on costs**								

For peer review only - http://bmjopen.bmj.com/site/about/guidelines.xhtml

Article	Disease area	Intervention	Study Type	Purpose Type	Geographical area covered	Hospital Type	Facility/hospital Level	Source of data
Baynes et al 2019	Obstetrics	Providing care to women that have had an abortion	Cross sectional	Cost Analysis	Mwanza, Arusha and Zanzibar	NGO & Public	All	Records and interviews
Chris et al 2013	General	Health services	Cross sectional	Cost Analysis	National	All	All	Survey
Kabadi et al 2013	Stroke	Providing stroke care	Longitudinal	Cost Analysis	Mbeya and Dar es Salaam	NGO & Public	Tertiary referral	Survey and project data
Mengistu et al 2019	Direct obstetric complications	Emergency Obstetric and Neonatal Care	Cross sectional	Cost Analysis	Kigoma	Public	Tertiary, district hospital and primary care	Programme & hospital data

For peer review only - http://bmjopen.bmj.com/site/about/guidelines.xhtml

Article	Disease area	Intervention	Study Type	Purpose Type	Geographical area covered	Hospital Type	Facility/hospital Level	Source of data
Mosha et al 2010	Malaria	Diagnostic tools and drugs	Cross sectional	Cost analysis	Kilimanjaro region and Korogwe District, Tanga region	Public	District and dispensaries	Survey
Riewpaiboon et al 2014	Typhoid	Typhoid treatment/care	Longitudinal	Cost Analysis	Pemba and Zanzibar	Public	District hospital	Record review and interviews
Sicuri et al 2011	Malaria	Malaria treatment/care	Longitudinal	Cost Analysis	Not specified	Not specified	Not specified	Survey
Chaudhury et al 2016	Neonatal care	Birth attendants training on	Cross sectional	Cost Analysis	Mbeya	NGO & Public	All	Survey

For peer review only - http://bmjopen.bmj.com/site/about/guidelines.xhtml

Article	Disease area	Intervention	Study Type	Purpose Type	Geographical area covered	Hospital Type	Facility/hospital Level	Source of data
		neonatal resuscitation						
Penno et al 2015	Antimicrobial resistance	Surveillance for Bloodstream Infections for Sepsis Management in low resource setting	Modelling	Economic evaluation	Northern Tanzania	Not specified	Not specified	Laboratory records
Githang'a et 2020	Oncology	Oncology treatment: e.g. chemotherapy, off site	Cross-sectional	Economic evaluation	Mwanza	NGO	Tertiary referral	Hospital data & interviews

For peer review only - http://bmjopen.bmj.com/site/about/guidelines.xhtml

Article	Disease area	Intervention	Study Type	Purpose Type	Geographical area covered	Hospital Type	Facility/hospital Level	Source of data
		radiotherapy, surgery						
Guerriero et al 2010	Blood transfusion	Use of Tranexamic acid as a replacement for blood transfusion	Modelling	Economic evaluation	National	Not specified	Not specified	Literature

For peer review only - http://bmjopen.bmj.com/site/about/guidelines.xhtml

Article	Disease area	Intervention	Study Type	Purpose Type	Geographical area covered	Hospital Type	Facility/hospital Level	Source of data
Kimaro et al 2017	Advanced HIV	Providing antiretroviral therapy services to HIV-infected individuals presenting with advanced HIV disease	Cohort	Economic evaluation	Dar es Salaam	Public	Primary health center	Patient survey, health care worker diaries
Phillips et al 2015	Malaria	Malaria Rapid Diagnostic test (mRDT)	Modelling	Economic evaluation	National	All	All	Local program/ records

For peer review only - http://bmjopen.bmj.com/site/about/guidelines.xhtml

**Article**	**Disease area**	**Intervention**	**Study Type**	**Purpose Type**	**Geographical area covered**	**Hospital Type**	**Facility/hospital Level**	**Source of data**
Shayo et al 2017	Tuberculosis	Isoniazid preventive therapy	Cohort	Economic evaluation	Dar es Salaam	NGO, Public and private	Tertiary and primary care	Records and interviews
Wilson et al 2020	Neonatal care	Birth attendants training on neonatal resuscitation (59 participants)	Cross sectional	Financial planning	Zanzibar	Not specified	Not specified	Survey
**Studies reporting on forms and resource availability in critical care**								
Baker et al 2011	Critical care	Emergency and critical care	Cross sectional	Others (structure and resources	National	All	Tertiary, district, and primary care	Survey

For peer review only - http://bmjopen.bmj.com/site/about/guidelines.xhtml

**Article**	**Disease area**	**Intervention**	**Study Type**	**Purpose Type**	**Geographical area covered**	**Hospital Type**	**Facility/hospital Level**	**Source of data**
				available for emergency and critical care)				
Chalya et al 2011	Trauma	Trauma care	Cross sectional	Others (describe patient characteristics and health outcomes)	Mwanza	NGO	Referral hospital	Survey
Mkoka et al 2014	Obstetrics	Emergency obstetric care	Cross sectional	Others (availability	Kongwa	Public	Primary healthcare	Interviews

For peer review only - http://bmjopen.bmj.com/site/about/guidelines.xhtml

Article	Disease area	Intervention	Study Type	Purpose Type	Geographical area covered	Hospital Type	Facility/hospital Level	Source of data
				of medical supplies)				
Mtango et al 2019	Critical care	Critical care	Cross sectional	Others (doctors' experiences of ICU referral)	Dar es salaam	Public	Tertiary referral	Interviews
Murthy et al 2015	Critical care	N/A	Review	Others (estimate the current ICU capacity)	Ifakara, Dar es Salaam and Mwanza	NGO & Public	Tertiary referral	Literature
Mwandri et al 2020	Trauma	Baseline assessment of	Cross sectional	Others (assessing	Mbeya and Dar es Salaam	NGO & Public	Tertiary referral	Hospital registry data

For peer review only - http://bmjopen.bmj.com/site/about/guidelines.xhtml

Article	Disease area	Intervention	Study Type	Purpose Type	Geographical area covered	Hospital Type	Facility/hospital Level	Source of data
		trauma care provision		burden and care process)				
Mwiga & Monaghan 2020	Respiratory illness	Difficult airway trolley	Case study	Others (development of difficulty airway trolley)	Mbeya	NGO	Tertiary referral	Difficult airway trolley
Nicks et al 2012	Emergence medicine	N/A	Case study	Others (structure of health system and care processes)	National	All	All	Literature

For peer review only - http://bmjopen.bmj.com/site/about/guidelines.xhtml

Article	Disease area	Intervention	Study Type	Purpose Type	Geographical area covered	Hospital Type	Facility/hospital Level	Source of data
Papali et al 2019	Critical care	N/A	Review	Others (structure and organisation of adult ICU)	N/A	All	All	Literature
Penoyar et al 2012	Surgery	Surgical care	Cross sectional	Others (capacity of first referral hospitals)	National	All	District, regional and referral	Survey
Rajaguru et al 2019	Surgery	Surgery	Cross sectional	Others (operation and financing of a referral hospital)	Mbeya	NGO	Tertiary referral	Record review

For peer review only - http://bmjopen.bmj.com/site/about/guidelines.xhtml

Article	Disease area	Intervention	Study Type	Purpose Type	Geographical area covered	Hospital Type	Facility/hospital Level	Source of data
Reynolds et al 2012	General (particular injuries, pneumonia and psychosis)	Emergency care	Cross sectional	Others (Barriers to emergency care)	National	Public	Tertiary referral	Interview
Sawe et 2016	General	Emergency treatment	Cross sectional	Others (clinical presentation and resource utilization)	Dar es Salaam	Public	Tertiary referral	Hospital record
Staton et al 2017	Traumatic Brain Injury	Trauma care	Cross sectional	Others (describe	Kilimanjaro	NGO	Referral Hospital	Survey

For peer review only - http://bmjopen.bmj.com/site/about/guidelines.xhtml

Article	Disease area	Intervention	Study Type	Purpose Type	Geographical area covered	Hospital Type	Facility/hospital Level	Source of data
				quality of care for TBI)				
Sylvanus et al 2019	Renal failure	Renal failure treatment and management	Cohort	Others (renal failure case management)	Dar es Salaam	Public	Tertiary referral	Survey
Zimmerman et al 2020	Acute traumatic brain injury	Quantifying the effect on outcomes from delays in the care of trauma	Cross sectional	Others (association of hospital delays and poor outcomes)	Moshi	NGO	Tertiary referral	Hospital data

For peer review only - http://bmjopen.bmj.com/site/about/guidelines.xhtml

Supplementary 4: Availability of resources required for critical care provision

**Resources**	**Study**			
	Penoyar et al 2012 (N=40 hospitals)	*Staton et al 2017 (patients; N=893)*	*Zimmerman et al 2020 (patients; N=3209)*	*Baker et al 2013 (N=10 hospitals)*
Availability of oxygen when needed	42%	22.9%	20.1%	
Oxygen concentrators	75%			
Mask and tubing to connect to oxygen supply	46%			
Pulse oximetry	13%			70%
Oxygen cylinders	35%			
Mechanical ventilators				40%
Oropharyngeal airway-adult	42%			60%
Oropharyngeal airway-paediatric	21%			
Personal protective wear (gloves)	90%			90%
Personal protective wear (aprons)	81%			
Personal protective wear (face masks)	69%			
Personal protective wear (eye protection)	40%			
Consistent running water	37.5%			100%
Consistent running electricity	37.5%			50%
Staff trained in critical care				20%

Supplementary 5: Appraisal Checklist

For peer review only - http://bmjopen.bmj.com/site/about/guidelines.xhtml

Study ID	Author	#1 Question(s)	#2 Perspective	#3 Time horizon	#4 Relevant inputs	#5 Methods for quantities	#6 Data source(s)	#7 Sample size	#8 Discount rate	#9 Sensitivity analysis	#10 Costs reporting
1	Githang'a et 2020	✓	✓	(✓)	✓	✓	✓		✓	✓	✓
2	Mengistu et al 2019	✓	✓	✓	✓	✓	✓			X	✓
3	Wilson et al 2020	✓	✓	**X**	✓	✓	✓			X	✓
4	Kimaro et al 2017	✓	✓	✓	✓	✓	✓			✓	✓
5	Shayo et al 2017	✓	✓	✓	✓	✓	✓			✓	✓
6	Phillips et al 2015	✓	✓	✓	✓	✓	✓			X	✓
7	Penno et al 2015	✓	✓	X	✓	✓	✓			✓	✓
8	Kabadi et al 2013	✓	✓	✓	✓	✓	✓			X	✓
9	Sicuri et al 2011	✓	✓	X	✓	✓	✓			✓	✓

For peer review only - http://bmjopen.bmj.com/site/about/guidelines.xhtml

For peer review only

10	Guerriero et al 2010	✓	✓	X	✓	✓	✓			✓	✓
11	Baynes et al 2019	✓	✓	(✓)	✓	✓	✓			✓	✓
12	Riewpaiboon et al 2014	✓	✓	(✓)	✓	✓	✓			✓	✓
13	Sumona et al 2016	✓	✓	(✓)	✓	✓	✓			✓	✓
14	James et al 2013	✓	✓	✓	✓	✓	✓			X	✓

Key: ✓ addressed; (✓) partially addressed; X Not addressed

Supplementary 6: Cost of diagnostics

Study	Treatment/disease area	Description	Unit of output	Currency year	Unit cost Price in 2019 (USD)	Unit cost Price in 2019 (TZS)
Mengistu et al 2019	Emergency obstetric care	Laboratory	per programme	2013	9.72	22249.85
Riewpaiboon et al 2014	Typhoid fever	Blood count	Per test	2010	1.64	3751.89
Riewpaiboon et al 2014	Typhoid fever	Blood chemistry	Per test	2010	2.46	5627.84
Kabadi et al 2013	Stroke	Diagnostics for stroke (Blood, ECG, ECHO, CXR) - Rural area	Cost per patient	2005-2006	3.09	7065.07
Kabadi et al 2013	Stroke	CT Scan- rural area	Cost per patient	2005-2006	44.35	101473.18
Kabadi et al 2013	Stroke	Diagnostics for stroke (Blood, ECG, ECHO, CXR) - Urban area	Cost per patient	2005-2006	35.89	82117.17

For peer review only - http://bmjopen.bmj.com/site/about/guidelines.xhtml

Kabadi et al 2013	Stroke	CT Scan- urban area	Cost per patient	2005-2006	55.43	126841.48
Kabadi et al 2013	Stroke	Diagnostics for stroke (Blood, ECG, ECHO, CXR) - average	Cost per patient	2005-2006	13.34	30519.33
Kabadi et al 2013	Stroke	CT Scan- average	Cost per patient	2005-2006	47.81	109400.77
Baird et al 2014	Sepsis management	Integrated Management of Adolescent and Adult Illness assessment	Per test	2011	1.17	2677.59
Baird et al 2014	Sepsis management	Lab assessment for sepsis- Negative blood culture	Per test	2011	13.09	29952.25
Baird et al 2014	Sepsis management	Lab assessment for sepsis- positive blood culture	Per test	2011	23.00	52633.02
Baird et al 2014	Sepsis management	Lab assessment for sepsis- ID and susceptibilities positive blood culture	Per test	2011	82.55	188901.34
Phillips et al 2015	Malaria	Malaria rapid diagnostic test	Per test	2010	14.92	34142.23
Shayo et al 2017	TB	Sputum microscopy	Per test	2012	2.16	4945.33
Shayo et al 2017	TB	Suptum culture	Per test	2012	10.83	24778.14
Kimaro et al 2017	HIV - ART provision	CD4 Count test	Per test	2012	17.74	40592.88
Kimaro et al 2017	HIV - ART provision	Alanine aminotransferase (ALAT) test	Per test	2012	0.99	2266.61
Kimaro et al 2017	HIV - ART provision	creatinine test	Per test	2012	0.35	798.46
Kimaro et al 2017	HIV - ART provision	Haemoglobin test	Per test	2012	0.98	2240.85
Kimaro et al 2017	HIV - ART provision	Random blood glucose (RBG) test	Per test	2012	0.96	2189.34
Kimaro et al 2017	HIV - ART provision	Venereal disease reference laboratory (VDRL) test	Per test	2012	2.13	4868.05
Kimaro et al 2017	HIV - ART provision	Pregnancy test	Per test	2012	0.60	1365.12
Kimaro et al 2017	HIV - ART provision	Full blood count (FBC) test	Per test	2012	2.30	5254.41

For peer review only - http://bmjopen.bmj.com/site/about/guidelines.xhtml

Kimaro et al 2017	HIV - ART provision	Serum cryptococcal meningitis (CRAG) test	Per test	2012	4.46	10199.73
Kimaro et al 2017	HIV - ART provision	Xpert test	Per test	2012	21.47	49118.41
Kimaro et al 2017	HIV - ART provision	Collection and processing of cerebral spinal fluid	Per test	2012	18.34	41957.99
Kimaro et al 2017	HIV - ART provision	Chest X-ray	Per test	2012	3.58	8190.70
Kimaro et al 2017	HIV - ART provision	Smear microscopy test	Per test	2012	2.04	4662.00
Kimaro et al 2017	HIV - ART provision	Sputum culture (Lowenstein Jensen) for TB	Per test	2012	7.70	17617.72

Supplementary 7: Cost of human resources

**Study**	**Description**	**Unit of output**	**Currency year**	**Unit cost Price in 2019 (USD)**	**Unit cost Price in 2019 (TZS)**
Mengistu et al 2019	Total personnel for Emergency obstetric care	per patient	2013	103.01	235,711.74
Baynes et al 2019	Cost of Obstetrician/gynecologist on all complications	Per patient	2016	10.00	22,884.06
Baynes et al 2019	Cost of Anesthesiologist on all obstetric complications	Per patient	2016	0.00	0.00

For peer review only - http://bmjopen.bmj.com/site/about/guidelines.xhtml

Baynes et al 2019	Cost Anesthetist on all obstetric complications	Per patient	2016	2.23	5,112.40
Baynes et al 2019	Cost of Anesthetist Assistant on all obstetric complications	Per patient	2016	1.81	4,138.61
Baynes et al 2019	Cost of doctor/ medical officer on all obstetric complications	Per patient	2016	15.75	36,030.22
Baynes et al 2019	Cost of assistant medical officer on all obstetric complications	Per patient	2016	5.00	11,442.03
Baynes et al 2019	Cost of clinical officer on all obstetric complications	Per patient	2016	0.85	1,947.58
Baynes et al 2019	Cost of registered nurse or principal enrolled nurse on obstetric all complications	Per patient	2016	9.79	22,397.16
Baynes et al 2019	Cost of enrolled nurse or midwife on all obstetric complications	Per patient	2016	43.62	99,813.44
Baynes et al 2019	Cost of medical attendant on all obstetric complications	Per patient	2016	6.06	13,876.50
Baynes et al 2019	Cost of laboratory technician on all complications	Per patient	2016	3.19	7,303.42
Baynes et al 2019	Cost of laboratory assistant on all complications	Per patient	2016	1.70	3,895.16

For peer review only

Baynes et al 2019	Cost of Sonographer on all obstetric complications	Per patient	2016	2.87	6,573.08
Baynes et al 2019	Cost of pharmacist on all complications	Per patient	2016	2.55	5,842.74
Baynes et al 2019	Cost of drug dispenser on all complications	Per patient	2016	1.06	2,434.47
Baynes et al 2019	Average personnel costs at primary care level facilities for all complications	Per patient	2016	14.28	32,670.64
Baynes et al 2019	Average personnel care costs at district hospitals for all complications	Per patient	2016	8.97	20,522.62
Baynes et al 2019	Average personnel costs at Regional hospital for all complications	Per patient	2016	30.14	68,968.66
Baynes et al 2019	Average personnel costs for all complications across any level of hospital	Per patient	2016	19.16	43,844.88
Shayo et al 2017	Assistant medical officer	Monthly salary	2012	638.96	1,462,064.84
Shayo et al 2017	Nurse	Monthly salary	2012	313.34	716,994.91
Shayo et al 2017	Health Attendant	Monthly salary	2012	440.41	1,007,738.83
Shayo et al 2017	Assistant Nurse	Monthly salary	2012	338.60	774,793.40

For peer review only - http://bmjopen.bmj.com/site/about/guidelines.xhtml

Shayo et al 2017	Nurse Counsellors	Monthly salary	2012	819.44	1,875,051.02
Shayo et al 2017	Medical Doctors	Monthly salary	2012	1473.56	3,371,810.40
Shayo et al 2017	Medical Specialists	Monthly salary	2012	2721.79	6,228,019.13
Shayo et al 2017	Laboratory Scientific Officer	Monthly salary	2012	1106.07	2,530,924.80
Shayo et al 2017	Laboratory technician	Monthly salary	2012	819.44	1,875,051.02
Shayo et al 2017	Pharmacist	Monthly salary	2012	1106.07	2,530,924.80
Shayo et al 2017	Pharmacy technician	Monthly salary	2012	819.44	1,875,051.02

For peer review only

PRISMA 2020 Checklist

Section and Topic	Item #	Checklist item	Location where item is reported
**TITLE**			
Title	1	Identify the report as a systematic review.	Title page
**ABSTRACT**			
Abstract	2	See the PRISMA 2020 for Abstracts checklist.	Pages 2 & 3
**INTRODUCTION**			
Rationale	3	Describe the rationale for the review in the context of existing knowledge.	Page 5 & 6 Lines 79 - 91
Objectives	4	Provide an explicit statement of the objective(s) or question(s) the review addresses.	Page 6, Lines 93 - 94
**METHODS**			
Eligibility criteria	5	Specify the inclusion and exclusion criteria for the review and how studies were grouped for the syntheses.	Page 8, Lines 123-125
Information sources	6	Specify all databases, registers, websites, organisations, reference lists and other sources searched or consulted to identify studies. Specify the date when each source was last searched or consulted.	Page 6, Lines 105-106
Search strategy	7	Present the full search strategies for all databases, registers and websites, including any filters and limits used.	Supplementary 1
Selection process	8	Specify the methods used to decide whether a study met the inclusion criteria of the review, including how many reviewers screened each record and each report retrieved, whether they worked independently, and if applicable, details of automation tools used in the process.	Page 8, Lines 118-120
Data collection process	9	Specify the methods used to collect data from reports, including how many reviewers collected data from each report, whether they worked independently, any processes for obtaining or confirming data from study investigators, and if applicable, details of automation tools used in the process.	Page 8, Lines 128-131
Data items	10a	List and define all outcomes for which data were sought. Specify whether all results that were compatible with each outcome domain in each study were sought (e.g. for all measures, time points, analyses), and if not, the methods used to decide which results to collect.	Page 9, Lines 133-138
	10b	List and define all other variables for which data were sought (e.g. participant and intervention characteristics, funding sources). Describe any assumptions made about any missing or unclear information.	Page 8, Lines 128-131
Study risk of bias assessment	11	Specify the methods used to assess risk of bias in the included studies, including details of the tool(s) used, how many reviewers assessed each study and whether they worked independently, and if applicable, details of automation tools used in the process.	Page 9, Lines 142-146
Effect measures	12	Specify for each outcome the effect measure(s) (e.g. risk ratio, mean difference) used in the synthesis or presentation of results.	N/A
Synthesis methods	13a	Describe the processes used to decide which studies were eligible for each synthesis (e.g. tabulating the study intervention characteristics and comparing against the planned groups for each synthesis (item #5)).	Page 9, Lines 133-137
	13b	Describe any methods required to prepare the data for presentation or synthesis, such as handling of missing summary statistics, or data conversions.	N/A
	13c	Describe any methods used to tabulate or visually display results of individual studies and syntheses.	Page 9, Lines 133-137
	13d	Describe any methods used to synthesize results and provide a rationale for the choice(s). If meta-analysis was performed, describe the model(s), method(s) to identify the presence and extent of statistical heterogeneity, and software package(s) used.	N/A
	13e	Describe any methods used to explore possible causes of heterogeneity among study results (e.g. subgroup analysis, meta-regression).	N/A

For peer review only
For peer review only - http://bmjopen.bmj.com/site/about/guidelines.xhtml

PRISMA 2020 Checklist

Section and Topic	Item #	Checklist item	Location where item is reported
	13f	Describe any sensitivity analyses conducted to assess robustness of the synthesized results.	N/A
Reporting bias assessment	14	Describe any methods used to assess risk of bias due to missing results in a synthesis (arising from reporting biases).	N/A
Certainty assessment	15	Describe any methods used to assess certainty (or confidence) in the body of evidence for an outcome.	Page 9, Lines 142-146
**RESULTS**			
Study selection	16a	Describe the results of the search and selection process, from the number of records identified in the search to the number of studies included in the review, ideally using a flow diagram.	Figure 1
	16b	Cite studies that might appear to meet the inclusion criteria, but which were excluded, and explain why they were excluded.	N/A
Study characteristics	17	Cite each included study and present its characteristics.	Table 2
Risk of bias in studies	18	Present assessments of risk of bias for each included study.	Supplementary
Results of individual studies	19	For all outcomes, present, for each study: (a) summary statistics for each group (where appropriate) and (b) an effect estimate and its precision (e.g. confidence/credible interval), ideally using structured tables or plots.	Tables 3-6
Results of syntheses	20a	For each synthesis, briefly summarise the characteristics and risk of bias among contributing studies.	N/A
	20b	Present results of all statistical syntheses conducted. If meta-analysis was done, present for each the summary estimate and its precision (e.g. confidence/credible interval) and measures of statistical heterogeneity. If comparing groups, describe the direction of the effect.	N/A
	20c	Present results of all investigations of possible causes of heterogeneity among study results.	N/A
	20d	Present results of all sensitivity analyses conducted to assess the robustness of the synthesized results.	N/A
Reporting biases	21	Present assessments of risk of bias due to missing results (arising from reporting biases) for each synthesis assessed.	N/A
Certainty of evidence	22	Present assessments of certainty (or confidence) in the body of evidence for each outcome assessed.	N/A
**DISCUSSION**			
Discussion	23a	Provide a general interpretation of the results in the context of other evidence.	Pages 40-44, Lines 294-369
	23b	Discuss any limitations of the evidence included in the review.	Pages 45-46, Lines 381- 391
	23c	Discuss any limitations of the review processes used.	Pages 45-46, Lines 381- 391
	23d	Discuss implications of the results for practice, policy, and future research.	Page 46, Lines 395-402
**OTHER INFORMATION**			
Registration and protocol	24a	Provide registration information for the review, including register name and registration number, or state that the review was not registered.	Page 6, Line 100
	24b	Indicate where the review protocol can be accessed, or state that a protocol was not prepared.	Page 6, Lines 100-101

For peer review only - http://bmjopen.bmj.com/site/about/guidelines.xhtml

PRISMA 2020 Checklist

Section and Topic	Item #	Checklist item	Location where item is reported
	24c	Describe and explain any amendments to information provided at registration or in the protocol.	N/A
Support	25	Describe sources of financial or non-financial support for the review, and the role of the funders or sponsors in the review.	Page 58, Lines 579-580
Competing interests	26	Declare any competing interests of review authors.	Page 58, Line 575
Availability of data, code and other materials	27	Report which of the following are publicly available and where they can be found: template data collection forms; data extracted from included studies; data used for all analyses; analytic code; any other materials used in the review.	Available

Based on Page MJ, McKenzie JE, Bossuyt PM, Boutron I, Hoffmann TC, Mulrow CD, et al. The PRISMA 2020 statement: an updated guideline for reporting systematic reviews. BMJ 2021;372:n71. doi: 10.1136/bmj.n71

For peer review only

PRISMA 2020 for Abstracts Checklist

Section and Topic	Item #	Checklist item	Reported (Yes/No)
**TITLE**			
Title	1	Identify the report as a systematic review.	Yes
**BACKGROUND**			
Objectives	2	Provide an explicit statement of the main objective(s) or question(s) the review addresses.	Yes
**METHODS**			
Eligibility criteria	3	Specify the inclusion and exclusion criteria for the review.	Yes
Information sources	4	Specify the information sources (e.g. databases, registers) used to identify studies and the date when each was last searched.	Yes
Risk of bias	5	Specify the methods used to assess risk of bias in the included studies.	Yes
Synthesis of results	6	Specify the methods used to present and synthesise results.	Yes
**RESULTS**			
Included studies	7	Give the total number of included studies and participants and summarise relevant characteristics of studies.	Yes
Synthesis of results	8	Present results for main outcomes, preferably indicating the number of included studies and participants for each. If meta-analysis was done, report the summary estimate and confidence/credible interval. If comparing groups, indicate the direction of the effect (i.e. which group is favoured).	Yes
**DISCUSSION**			
Limitations of evidence	9	Provide a brief summary of the limitations of the evidence included in the review (e.g. study risk of bias, inconsistency and imprecision).	Yes
Interpretation	10	Provide a general interpretation of the results and important implications.	Yes
**OTHER**			
Funding	11	Specify the primary source of funding for the review.	No
Registration	12	Provide the register name and registration number.	Yes

From: Page MJ, McKenzie JE, Bossuyt PM, Boutron I, Hoffmann TC, Mulrow CD, et al. The PRISMA 2020 statement: an updated guideline for reporting systematic reviews. BMJ 2021;372:n71. doi: 10.1136/bmj.n71

For more information, visit: http://www.prisma-statement.org/

For peer review only

BMJ Open

Resource use, availability and cost in the provision of critical care in Tanzania: A systematic review

Journal:	*BMJ Open*
Manuscript ID	bmjopen-2021-060422.R2
Article Type:	Original research
Date Submitted by the Author:	13-Oct-2022
Complete List of Authors:	Kazibwe, Joseph; London School of Hygiene & Tropical Medicine, Global Health and Development<br>Shah, Hiral A.; Imperial College London, Department of Infectious Disease Epidemiology; Center for Global Development<br>Kuwawenaruwa, A; Ifakara Health Institute, Health System Impact Evaluation and Policy Unit<br>Schell, Carl Otto; Karolinska Institutet, Department of Global Public Health; Uppsala University<br>Khalid, Karima; Muhimbili University of Health and Allied Sciences, Department of Anaesthesia and Critical Care<br>Tran, Phuong Bich; University of Antwerp, Department of Family and Population Health<br>Ghosh, Srobana; Center for Global Development, Global Health Department<br>Baker, Tim; London School of Hygiene & Tropical Medicine, Department of Clinical Research; Ifakara Health Institute, Department of Emergency Medicine<br>GUINNESS, LORNA; Center for Global Development
**Primary Subject Heading**:	Emergency medicine
Secondary Subject Heading:	Intensive care
Keywords:	INTENSIVE & CRITICAL CARE, HEALTH ECONOMICS, Health policy < HEALTH SERVICES ADMINISTRATION & MANAGEMENT

SCHOLARONE™
Manuscripts

I, the Submitting Author has the right to grant and does grant on behalf of all authors of the Work (as defined in the below author licence), an exclusive licence and/or a non-exclusive licence for contributions from authors who are: i) UK Crown employees; ii) where BMJ has agreed a CC-BY licence shall apply, and/or iii) in accordance with the terms applicable for US Federal Government officers or employees acting as part of their official duties; on a worldwide, perpetual, irrevocable, royalty-free basis to BMJ Publishing Group Ltd ("BMJ") its licensees and where the relevant Journal is co-owned by BMJ to the co-owners of the Journal, to publish the Work in this journal and any other BMJ products and to exploit all rights, as set out in our licence.

The Submitting Author accepts and understands that any supply made under these terms is made by BMJ to the Submitting Author unless you are acting as an employee on behalf of your employer or a postgraduate student of an affiliated institution which is paying any applicable article publishing charge ("APC") for Open Access articles. Where the Submitting Author wishes to make the Work available on an Open Access basis (and intends to pay the relevant APC), the terms of reuse of such Open Access shall be governed by a Creative Commons licence – details of these licences and which Creative Commons licence will apply to this Work are set out in our licence referred to above.

Other than as permitted in any relevant BMJ Author's Self Archiving Policies, I confirm this Work has not been accepted for publication elsewhere, is not being considered for publication elsewhere and does not duplicate material already published. I confirm all authors consent to publication of this Work and authorise the granting of this licence.

For peer review only

Resource use, availability and cost in the provision of critical care in Tanzania: A systematic review

Joseph Kazibwe,

1. Global Health and Development Department, London School of Hygiene and Tropical Medicine, London UK
2. ORCID - https://orcid.org/0000-0002-8315-1503

   Joseph.Kazibwe@lshtm.ac.uk

Hiral A. Shah,

1. MRC Centre for Global Infectious Disease Analysis, Department of Infectious Disease Epidemiology, School of Public Health, Imperial College London, UK.
2. Center for Global Development, London, UK.
3. ORCID - https://orcid.org/0000-0003-0204-451X

August Kuwawenaruwa,

1. Health System Impact Evaluation and Policy Unit, Ifakara Health Institute United Republic of Tanzania

Carl Otto Schell,

1. Department of Global Public Health, Karolinska Institutet, Stockholm, Sweden
2. Centre for Clinical Research Sörmland, Uppsala University, Eskilstuna, Sweden
   ORCID - https://orcid.org/0000-0002-7904-1336

Karima Khalid,

1. Department of Anaesthesia and Critical Care, Muhimbili University of Health and Allied Sciences, Dar es Salaam, Tanzania
2. Ifakara Health Institute, Dar-es-salaam, Tanzania
   ORCID - https://orcid.org/0000-0001-9917-9853

Phuong Bich Tran,

1. Department of Family and Population Health, University of Antwerp, Antwerpen, Belgium

   ORCID - https://orcid.org/0000-0003-2119-998X

Srobana Ghosh,

1. Global Health Department, Center for Global Development, London, UK

Tim Baker,

1. Department of Clinical Research, London School of Hygiene & Tropical Medicine, UK
2. Ifakara Health Institute, Dar es Salaam, Tanzania

For peer review only

For peer review only - http://bmjopen.bmj.com/site/about/guidelines.xhtml

3. Department of Emergency Medicine, Muhimbili University of Health and Allied Sciences, Dar es Salaam, Tanzania
4. Department of Global Public Health, Karolinska Institutet, Stockholm, Sweden
ORCID: 0000-0001-8727-7018

Lorna Guinness
1. Center for Global Development, London, UK
ORCID: https://orcid.org/0000-0002-1013-4200

Key words: Critical care, Costs of critical care, forms of critical care, Resources, Low- and middle-income countries (LMICs), Tanzania

Abstract

Objectives

Critical care is essential in saving lives of critically ill patients, however, provision of critical care across lower resource settings can be costly, fragmented and heterogenous. Despite the urgent need to scale-up the provision of critical care, little is known about its availability and cost. Here, we aim to systematically review and identify reported resource use, availability and costs for the provision of critical care and the nature of critical care provision in Tanzania.

Design

This is a systematic review following the Preferred Reporting Items for Systematic Reviews and Meta-Analyses (PRISMA) guidelines.

Data sources

Medline, Embase and Global Health databases were searched covering the period 2010 through to 17th November 2020.

For peer review only - http://bmjopen.bmj.com/site/about/guidelines.xhtml

Eligibility criteria

We included studies that reported on forms of critical care offered, critical care services offered and or costs and resources used in the provision of care in Tanzania published from 2010.

Data extraction and synthesis

Quality assessment of the articles and data extraction was done by two independent researchers. The Reference Case for Estimating the Costs of Global Health Services and Interventions was used to assess quality of included studies. A narrative synthesis of extracted data was conducted. Costs were adjusted and reported in 2019 USD and TZS using the World Bank GDP deflators.

Results

A total 31 studies were found to fulfil the inclusion and exclusion criteria. Critical care identified in Tanzania was categorised into: ICU delivered critical care and non-ICU critical care. The availability of ICU delivered critical care was limited to urban settings whereas non-ICU critical care was found in rural and urban settings. Paediatric critical care equipment were more scarce than equipment for adults. 15 studies reported on the costs of services related to critical care yet no study reported an average or unit cost of critical care. Costs of medication, equipment (e.g. oxygen, PPE), services, and human resources were identified as inputs to specific critical care services in Tanzania.

Conclusion

For peer review only - http://bmjopen.bmj.com/site/about/guidelines.xhtml

There is limited evidence on the resource use, availability and costs of critical care in Tanzania. There is a strong need for further empirical research on critical care resources availability, utilization and costs across specialties and hospitals of different level in low- and middle-income countries like Tanzania to inform planning, priority setting and budgeting for critical care services.

PROSPERO registration number: CRD42020221923

For peer review only

Strength and limitations of this study

- This study followed the Preferred Reporting Items for Systematic Reviews and Meta-Analyses PRISMA guidelines to produce the first systematic review of the literature around the costs and resources used in critical care and forms of critical care in a lower resource setting such as Tanzania.
- A broad and comprehensive search strategy was designed to ensure all studies on resource utilisation and costs of critical care were identified despite its heterogeneous nature.
- This study used a fully articulated population, intervention, comparison and outcome framework to develop the search strategy and identify key search terms.
- To address the likelihood of limited relevant data on critical care in Tanzania, we selected a set of diseases likely to require critical care (eg, malaria and pneumonia) to include in our search strategy which may have introduced some selection bias.
- The availability of literature was limited in this lower-middle income country setting.

For peer review only - http://bmjopen.bmj.com/site/about/guidelines.xhtml

Background

Critical care is an essential element of healthcare whose importance has been highlighted globally during the COVID-19 pandemic with countries experiencing overwhelming numbers of critically ill patients [1–3]. As a result, the care of critical illness has become an urgent point of focus in global health policy. COVID-19 notwithstanding, critical illness remains challenging driven by the large burden of injuries, infections and noncommunicable diseases [4,5]. There are an estimated 45 million cases of critical illness in adults in the word each year[4] yet the limited capacity to deliver critical care, especially in resource constrained settings, is likely to continue [6].

Critical care is the identification, monitoring and treatment of patients with critical illness through the initial and sustained support of vital organ functions[7]. It entails enhanced monitoring, treatment and attention, due to a life-threatening illness or injury[8]. There have been great advances in the provision of critical care for example invasive and non-invasive monitoring techniques, mechanical ventilation (MV), and renal replacement therapy, among others, resulting in reduced mortality rates in patients with critical illness, especially in high income countries[9].

However, such critical care is provided in sophisticated intensive care units using equipment which are costly to procure and maintain[10]. This makes it difficult for low resource settings to acquire such equipment to meet the needs of their populations and has led to recommendations for the development of more affordable critical care models that can be provided in any hospital settings and to all critically ill patients [1,11,12]. Moreover, most critically ill patients do not need critical care at ICU-level[13] and large unmet needs of even basic lifesaving critical care have been found in low-

For peer review only - http://bmjopen.bmj.com/site/about/guidelines.xhtml

and middle-income countries (LMICs)[14–16]. With various national and international stakeholders calling for critical care scale-up[17], it is necessary to understand the resources needed in the provision of critical care and their respective costs. Unfortunately, knowledge on the current availability of resources and the costs of critical care remains sparse[1,18,19]. There is no systematic review that examines the available resources, utilisation and costs of critical care generally[20] nor in LMIC settings.

To inform the planning and scale up of critical care provision in a low-income country setting, this study takes a systematic approach to review available knowledge on the current resource availability, utilisation and cost in the provision of critical care in Tanzania[20].

Methods

The systematic review followed the Preferred Reporting Items for Systematic Reviews and Meta-Analyses (PRISMA) guidelines[21] and the protocol was registered with PROSPERO; registration number: CRD42020221923[20]. The full methodological protocol used in this review is available elsewhere[20]. For this study, critical care is defined as the identification, monitoring and treatment of patients with critical illness through the initial and sustained support of vital organ functions[7]. It involves enhanced monitoring, treatment and attention required by a patient as a result of the life-threatening aspects of an illness or injury[8]. Some aspects of critical care can be provided in any location e.g. monitoring for vital signs and respiratory support. However, an ICU is a well-established unit intended to provide specialist critical care that sustains life of patients during

For peer review only - http://bmjopen.bmj.com/site/about/guidelines.xhtml

periods when a patient is experiencing a life-threatening condition. ICU critical care is critical care offered in an ICU while a non-ICU critical care is critical care offered in a place that is not gazetted as an ICU.

Search strategy and selection process

Following PRISMA guidelines, 3 electronic databases (i.e. Medline, Embase and Global Health) were systematically searched for articles published since 2010 [22]. A ten-year time frame was considered to ensure that findings reflect the present-day resource utilization paradigm as resources used and their costs vary as technology and clinical guidelines evolve. Bibliographies of included articles were reviewed to find relevant articles that fulfil the inclusion criteria but had not been identified. Google and Google Scholar were also used to search for published articles that may not have been indexed within the databases. As internet search engines typically return several thousand results, the searches were restricted to the first fifty hits and links to potentially relevant material were accessed[23].

Table 1PICO (Population, Intervention, Comparator, Outcome) Framework used to inform the search strategy

Element	Description
Population	Any patient in need of critical care
Intervention	Inpatient services that could form part of critical care provision
Comparison	No critical care
Outcomes	Critical care services available<br>Cost of the different inputs to critical care provision per patient

For peer review only - http://bmjopen.bmj.com/site/about/guidelines.xhtml

	Quantity and type of resources used in provision of critical care per patient

The PICO framework (table 1) describes major components used to identify key words. Other concepts and search terms are listed in the supplementary material. Key words included critical care, critical illness, resource use and setting. To ensure that no costings were missed, we further added hospital care and selected condition with high disease burden in Tanzania to the search string (for example neonatal disorders, HIV, Malaria and TB[24]), on the premise that these are conditions that have a high risk of critical illness. See supplementary 1 for detailed key words.

Eligibility criteria

A study was considered eligible if it was published in English and reported on the provision of critical care[25], services offered under critical care, resources and or costs incurred by the health system or health providers in providing critical care in Tanzania. A detailed inclusion/ exclusion criterion can be found in supplementary 2.

Study selection

The selection process followed the PRISMA guidelines. The articles identified during the search were retrieved and uploaded to Rayyan QCRI software[26]. The software was used to remove the duplicates. A standard process of screening titles and abstracts and full text reading was followed in assessment of eligibility of articles. Although some papers were focussed on a specific condition, those that described including the costs of critical care and/or emergency related to that condition were selected. While many diagnostics are not specific to critical care, they can be used in the delivery of critical care. We therefore also included papers that reported on diagnostic costs.

For peer review only - http://bmjopen.bmj.com/site/about/guidelines.xhtml

During the assessment of eligibility of the articles, two independent researchers of LG, HAS, SG, PBT and JK first reviewed the title and abstract. Articles that were identified as still eligible were then assessed through full text review by at least two researchers (SG, JK, HAS, PBT and LG). All conflicts were discussed and agreed on through consensus with third researcher (LG or HAS).

Data extraction and synthesis

A bespoke data extraction form was developed and used to record extracted data. The extracted data included author, year of publication, context (location, setting- urban or rural, type of facility, level of facility), critical care services offered, critical care equipment available, costing perspective, costing year, currency used, type of provider, payer, source of cost data, costing time frame, direct medical costs, resources used.

A narrative synthesis of extracted data was conducted to report an overview of study characteristics of the eligible studies. We described the characteristics of critical care provision as identified in the literature and resources available for the provision of critical care in Tanzania. To facilitate comparisons, all reported costs were adjusted to 2019 prices using the GDP deflators for Tanzania[27] and reported in USD and TZS. Cost data was collated first at the service level - by specific patient level service delivered. Then costs and resources used were categorized into groups based on a standard input-wise categorisation, for example, human resources, diagnostics, services, consumables and medication.

Quality assessment of individual studies

For peer review only - http://bmjopen.bmj.com/site/about/guidelines.xhtml

The quality of the included articles was assessed by two in dependent researchers using an adaptation of the Reference Case for Estimating the Costs of Global Health Services and Interventions[28], which provides a framework for quality assessment and data extraction. We used this framework to check the transparency of the authors in reporting what they did and identify likely sources of bias. With the framework we described the following; any sub group or population analysis done, statistical methods used to establish differences in unit costs by sub-group, determinants of cost, multivariate statistical methods used to analyze cost functions, sensitivity analyses conducted, list possible sources of bias, aspects of the cost estimates that would limit generalizability of results to other constituencies, all pecuniary and non-pecuniary interests of the study. Furthermore, methodological quality of studies that reported costs were further assessed by an appraisal checklist by Adawiyah et al[29]. We used ten parameters to further assess the methodological quality which included research question being tackled, perspective, time horizon, relevant inputs, methods for quantities, data sources, sample size, discount rate, sensitivity analysis, and reporting of costs. Any discrepancies were addressed by a joint re-evaluation of the article among 3 authors.

Patient and Public Involvement

This systematic review did not involve any patient nor public in the design, data extraction and analysis. However, the findings of this systematic review will be made available to the public through an open access manuscript.

Ethical approval

For peer review only - http://bmjopen.bmj.com/site/about/guidelines.xhtml

Ethical clearance was sought from and granted by the Tanzanian National Institute of Medical Research (reference: NIMR/HQ/R.8a/Vol. IX/3537) and London School of Hygiene and Tropical Medicine (ethics ref: 22866).

[Insert figure 1: PRISMA Chart]

Results

Study characteristics

A total of 792 articles were found using the search and 10 articles from the reference list review. Thirty-one studies fulfilled the inclusion criteria (figure 1), of which 14 reported on the costs associated with critical care in Tanzania (please see supplementary 3: table of study characteristics (N=31)). The disease areas covered by eligible studies included; oncology (n=1), trauma (n=4), obstetrics (n=3), neonatal/child care (n=2), HIV (n=1), renal failure (n=1), TB (n=1), malaria (n=3), stroke (n=1) and typhoid (n=1), antimicrobial resistance (n=1) surgery (n=2) respiratory illness (n=1), blood transfusion (n=1), no disease or area specific (n=8). The majority of studies were cross sectional in design (n=20) and reported on the location of critical care and critical care services available.

Most studies were conducted in a specific region: the Coast Zone (n = 7), Lake Zone (n = 3), Northern Highland Zone (n = 3), Southern Highland Zone (n = 3) and Central Zone (n = 1). A zone is a geographical area made up of districts within a given health region. Seven studies were nationally representative (n = 7); two studies did not specify a given region. Three studies were carried out in rural settings while the rest were urban or mixed settings. Of the studies that

For peer review only - http://bmjopen.bmj.com/site/about/guidelines.xhtml

specifically reported on critical care provision, thirteen of the fifteen studies were carried out in urban settings, two of the fifteen studies were carried out in both rural and urban settings and no study was exclusively carried out in a rural setting. Nine studies specified that they were conducted in public (government owned) facilities and six specified non-governmental organisations including faith/missionary-based hospitals.

Critical care provision in Tanzania

The structure of the publicly funded health system in Tanzania is comprised of six levels[30] starting with the village health post (lowest level), dispensaries, health centres, district hospitals, regional hospitals and tertiary referral hospitals (the highest level) and care is delivered by public, faith/missionary-based providers and private providers [30–43]. Critical care is usually offered at the hospital level only, that is the district hospital, regional hospitals and referral hospitals [30–35,37–44]. In addition, Nicks et al state that district hospitals offer basic forms of critical care while referral hospitals offer critical care in ICUs[30].

Based on the literature, we further categorise critical care into "Critical care delivered in an ICU" and "Critical care delivered outside the ICU". ICU delivered critical care services included intubation and ventilation. For example, one study found that 54.2% of all ICU patients were intubated or ventilated[42]. In total, seven studies reported on ICU delivered critical care [32,33,39,40,42,43]. The ICUs were all based in tertiary referral hospitals in Tanzania including regional hospitals. For example, Murthy et al state that St. Francis Hospital in Ifakara region had 10 ICU beds and Sekou Toure Regional Referral Hospital in Mwanza had 8 ICU beds[37]. The district hospitals, for which studies were available, did not provide ICU delivered critical care [38].

For peer review only - http://bmjopen.bmj.com/site/about/guidelines.xhtml

Critical care outside the ICU was reported to be delivered either in operating theatres or in emergency units and general wards. For example, operating theatres at Kilimanjaro Christian Medical Centre (KCMC) offered critical care for cases like trauma and sepsis[45]. Theatres in lower-level hospitals like district hospitals often offer initial critical care before referring the patient to higher level hospitals that can offer ICU based critical care. Although district hospitals and some regional hospitals were reported not to have ICUs, these facilities were reported to offer critical care in the general wards[38]. Some hospitals without ICUs designated areas within general wards to cater for the critically ill patients where arterial blood gas and central venous pressure monitoring are done[38]. Administration of critical care related medicines like Mannitol, and airway management were carried out in general wards.[43] Furthermore, Staton et al found that some critical care services including intubation were performed in the emergency units[43].

Availability of resources required for critical care

The majority of eligible studies reported on the known resources used for critical care (n=21). However, many studies did not report the extent to which the resources were readily available at a given hospital (n=18). Three studies reported on availability and use of critical care resources and indicate that their availability varies between hospitals and is sometimes unreliable or inconsistent (supplementary 4). Two studies find that there is a scarcity of trained staff in critical care[38,41]. Further, the availability of critical care equipment was heterogenous, with scarcity in the areas of mask and tubing, pulse oximetry, oxygen cylinders, adult and paediatric

For peer review only - http://bmjopen.bmj.com/site/about/guidelines.xhtml

oropharyngeal airway, PPE eye protection while gloves were abundant. In addition, a greater scarcity in paediatric than adult critical care equipment was identified [38,41].

Quality assessment

Of the 31 studies, 14 reported on the resource use and critical care costs and were appraised for quality using the Adawiyah et al adapted appraisal checklist[29] (Supplementary 5). All 14 studies were found to have clear research questions, mentioned the specific costing perspective, included relevant inputs, clearly stated methods for quantifying resources and reported costs. Four studies did not specify the time horizon while four studies partially addressed it. The majority of the studies (n=8) carried out some form of sensitivity analysis. All studies that collected primary data stated sample size but did not mention how it was determined and whether it was sufficient.

Resource use and cost of resources associated with provision of critical care

Types of costs reported and sources of data

Studies that published costs of critical care as shown in table 2, reported economic costs (n = 8) or financial costs (n = 6), using a mix of bottom up (n = 9) and top down (n = 5) approaches. Sources of the costs were local data collected from hospital records, surveys and price lists. Surveys (including key informant interviews) were a common method of data collection (n = 9). Three studies used patient level data on resource use to inform costs[46–48]. Hospital or laboratory records were used in 6 of the 14 studies. Some studies used both survey and records review as the source of data (n = 6). Two studies used international literature as a source of data (n =2). Unit costs reported included cost per program, cost per patient, cost per visit, cost per patient day, cost per episode, cost per bed day, inpatient cost, total annual cost, cost per case.

For peer review only - http://bmjopen.bmj.com/site/about/guidelines.xhtml

Table 2: Types of costs reported and sources of data

Study	Type of costs*	Real world /guideline cost	Full/incremental cost	Costing approach**	Source of cost data
Githang'a et 2020	Financial	Real world costs	Full costs	Mixed	Hospital data
Mengistu et al 2019	Financial	Real world costs	Full costs	Top-down	Programme, hospital and health center data
Wilson et al 2020	Financial	Real world costs	Full costs	Bottom-up	Staff Survey (N=59)
Kimaro et al 2017	Economic	Real world costs	Full costs	Bottom-up	Patient survey, diaries (N=870)
Shayo et al 2017	Economic	Real world costs	Full costs	Bottom-up	Records, government standards and market price survey
Phillips et al 2015	Economic	Real world costs	Full costs	Top down	Local program records
Penno et al 2015	Economic	Real world costs	Full costs	Bottom-up	Laboratory records
Kabadi et al 2013	Economic	Real world costs	Full costs	Bottom-up	Survey (N=16) and project data
Sicuri et al 2011	Economic	Real world costs	Full costs	Bottom-up	Patient Survey (N=150)
Guerriero et al 2010	Economic	Real world costs	Full costs	N/A	Literature
Baynes et al 2019	Financial	Real world costs	Full costs	Bottom-up	Health facility Records (N=31) and key informant interviews (N=124)
Riewpaiboon et al 2014	Economic	Real world costs	Full costs	Bottom-up	Record review and patient survey (N=17)
Sumona et al 2016	Financial	Real world costs	Full costs	Bottom-up	Health facility Survey (N=386)
James et al 2013	Financial	Real world costs	Full costs	Top-down	Facility Survey (N=155)

*Economic costs are costs that capture opportunity i.e. the value of the forgone option/item including any donations or volunteer time, financial costs are the amount/money paid for a good or service.

**Top down cost estimation method is the estimation of cost by dividing the total cost by the quantity of services delivered or activities carried out, bottom up cost estimation method is based on the valuation of all resources used in the process of delivering/providing a good or service. Mixed approaches use top down methods for certain cost categories and bottom up for other categories where more precision is required.

Cost of services offered to patients

Table 3: Cost of services offered to patients

Study	Description	Unit cost of output	Currency year	Unit cost Price in 2019 (USD)	Unit cost Price in 2019 (TZS)
Mengistu et al 2019	Clinical care for emergency obstetric case	per case	2013	143.85	329,166.
Mengistu et al 2019	Average cost per surgical patient	per patient	2013	285.08	652,333.26
Mengistu et al 2019	Average cost per patient undergoing caesarean	Per patient	2013	471.28	1,078,396.41
Riewpaiboon et al 2014	Inpatient hospital cost	Per patient per day	2010	91.78	107.49
Sicuri et al 2011	Episode of Malaria hospitalisation and Severe anaemia	per episode	2009	57.12	130,704.61
Sicuri et al 2011	Episode of Cerebral malaria	per episode	2009	22.67	51,864.33
Sicuri et al 2011	Episode of Cerebral malaria and neurological sequela	per episode	2009	47.58	108,879.84
Kabadi et al 2013	Hospitalisation of stroke patient (Inpatient, Drugs, Registration)- Rural area	per patient	2005-2006	12.76	29,200.18

For peer review only - http://bmjopen.bmj.com/site/about/guidelines.xhtml

Kabadi et al 2013	Hospitalisation of stroke patient (Inpatient, Drugs, Registration)- Urban area	per patient	2005-2006	31.17	71,317.89
Kabadi et al 2013	Physiotherapy for stroke- urban area	per patient	2005-2006	44.88	102,697.20
Kabadi et al 2013	Hospitalisation of stroke patient (Inpatient, Drugs, Registration)- average	per patient	2005-2006	18.51	42,361.25
Kabadi et al 2013	Physiotherapy for stroke- average	per patient	2005-2006	14.03	32,093.43
James, Bura & Ensor 2013	Health centre: overall	Inpatient day	2011/12	18.03	41,261.22
James, Bura & Ensor 2013	Health centre: public	Inpatient day	2011/12	18.98	43,432.74
James, Bura & Ensor 2013	health centre: NGO	Inpatient day	2011/12	17.29	39,564.68
James, Bura & Ensor 2013	Hospital: all	Inpatient day	2011/12	25.95	59,387.93
James, Bura & Ensor 2013	Hospital: public	Inpatient day	2011/12	18.54	42,422.55
James, Bura & Ensor 2013	Hospital: NGO	Inpatient day	2011/12	15.76	36,062.52
James, Bura & Ensor 2013	Hospital: private	Inpatient day	2011/12	68.05	155,721.31
James, Bura & Ensor 2013	Hospital regional	Inpatient day	2011/12	17.33	39,653.32
James, Bura & Ensor 2013	Hospital - ARI-pneumonia	per case treated	2011/12	104.33	238,736.91
James, Bura & Ensor 2013	Hospital - Hypertension	per case treated	2011/12	253.98	581,166.07
James, Bura & Ensor 2013	Hospital - Myocardial infarction	per case treated	2011/12	268.57	614,533.21

For peer review only

For peer review only - http://bmjopen.bmj.com/site/about/guidelines.xhtml

Although many studies in table 3 reported costs of differing disease specific (e.g. malaria and anaemia) or general hospital services (e.g. inpatient and outpatient care) that included treatment of critical illness, no study reported the specific cost of the treatment of the critical care. A nationally representative study of general hospital services including critical care found that unit costs vary by provider ownership (public, NGO and private) and level of health facility (regional vs primary care) [49]. Services provided at private hospitals were more expensive than the same services received at public hospitals. NGO hospitals were less expensive than public hospitals[49].

Cost of Equipment, consumables and medication

Table 4: Cost of Equipment, consumables and medication

Study	Description	Unit cost of output	Currency year	Unit cost Price in 2019 (USD)	Unit cost Price in 2019 (TZS)
**Equipment**					
Chaudhury et al 2016	Mannequin	per piece	2014	76.03	173,970.65
Chaudhury et al 2016	Bag-mask device	per piece	2014	16.29	37,279.43
Chaudhury et al 2016	Penguin sucke	per piece	2014	3.26	7,455.89
Mengistu et al 2019	Equipment needed for emergency obstetric care	per patient	2013	90.02	205,994.65
**Consumables**					
Mengistu et al 2019	Other supplies for emergency obstetric care	per patient	2013	28.68	65,635.80
Mengistu et al 2019	Blood supplies	per patient	2013	0.71	1,620.01
Guerriero et al 2010	Consumables needed in the administration of tranexamic acid (10ml syringe,100ml bag of saline, large gauge needle)	Per dose	2007	2.00	4,576.41
**Medication**					
Analgesics and sedatives					
Riewpaiboon et al 2014	Paracetamol 120 mg/5 mL in 60-mL bottle	per bottle	2010	0.33	750.38
Riewpaiboon et al 2014	Paracetamol 500 mg tablet	Per tablet	2010	0.01	26.80
Antifungal and antibacterial drugs					
Riewpaiboon et al 2014	Ampicillin 500 mg vial injection	Per piece	2010	0.21	482.39
Riewpaiboon et al 2014	Ceftriaxone 250 mg vial injection	Per piece	2010	0.46	1,045.17
Riewpaiboon et al 2014	Ciprofloxacin 250 mg capsule	Per Capsule	2010	0.01	26.80
Riewpaiboon et al 2014	Ciprofloxacin 500 mg capsule	Per capsule	2010	0.05	107.20
Penno et al 2015	Antimicrobial treatment using generic drugs for sepsis management	per case	2011	15.17	34,703.66
Penno et al 2015	Evidence-based antimicrobials	Per case	2011	30.36	69,459.83
Kimaro et al 2017	Cotrimoxazole per daily dose	Per daily dose	2012	0.02	51.51

For peer review only - http://bmjopen.bmj.com/site/about/guidelines.xhtml

Others like anti-cancers					
Githang'a et al 2020	50 mg of 6-mercaptopurine (6MP)	Unclear	2013	0.39	885.94
Mengistu et al 2019	Drugs for emergency obstetric care	per patient	2013	6.90	15,795.12
Riewpaiboon et al 2014	Glucose (5%) 500 mL	Per unit	2010	0.50	1,152.37
Guerriero et al 2010	Tranexamic acid	Per dose	2007	9.74	22,279.66

When looking at cost categories in table 4, two studies reported costs of equipment, two studies reported costs of consumables and six studies reported medication cost. Within studies, we found per item cost for equipment (4 items), consumables (3 items) and medication (13 items). The cost of a mannequin used in training was the most expensive piece of equipment reported (USD 76.03)[50]. In the medication category, tranexamic acid was the most expensive per dose (USD 9.74)[51], whereas antimicrobials were the most expensive on a per case basis (USD 30.36)[52].

Cost of diagnostics

Six studies reported on the cost of diagnostics, for example, full blood count (FBC), serum cryptococcal meningitis test, sputum culture and imaging. Lab assessment for sepsis- ID and susceptibilities positive blood culture was the most expensive diagnostic test (USD 82.55) followed by CT scans (USD 47.81 to 44.35) (see supplementary 6 for details).

Costs of human resources

Three studies reported human resource costs (see supplementary 7). However, the breakdown of human resource costs presented in the papers did not allow for a separation of costs between treatment for the condition and the critical illness care.   Reporting of human resource cost was heterogenous. One study provided monthly salaries for all personnel with monthly salaries ranging from USD 2721.79 for medical specialists USD 313.34 for nurses[53]. Another study reported total cost of personnel as a line item[54]. The third study reported unit costs (i.e. cost per patient) treatment for all obstetric complications broken down by staff category[55]. This study

For peer review only - http://bmjopen.bmj.com/site/about/guidelines.xhtml

found that the highest cost was for an enrolled nurse or midwife (USD 43.62 per patient) and that the lowest unit personnel cost was for an anaesthesiologist (less than 0.01USD per patient)[55].

Discussion

Our review found limited evidence on resource use, availability and costs of critical care in Tanzania. Of the eligible evidence, we found high variability in the geographical distribution and availability of resources needed for both ICU and non-ICU critical care provision. We found that ICU critical care was provided at the tertiary level in urban areas and no published provision in rural areas, whereas, non-ICU critical care was evident in both rural and urban areas. Within hospitals, we find that availability of key critical care resources (e.g. pulse oximeters, oxygen cylinders, mask and tubing) can be limited but the use of these resources is not documented. We, finally, find no reported estimates on the unit costs of critical care; and that the generation and reporting of cost data is not standardised thereby limiting synthesis or aggregation.

Availability of critical care in Tanzania

The majority of ICUs identified in our systematic review were located in tertiary referral hospitals and private hospitals located in urban areas (Dar es Salaam, Moshi, Mwanza, Mbeya city). This is of particular concern in Tanzania where the majority of the population (64.77) lives in rural areas[56]. Although this inequity in service distribution is not unique to Tanzania as previous studies in LMICs found all hospitals with ICUs were located in cities [37] , it is further

For peer review only - http://bmjopen.bmj.com/site/about/guidelines.xhtml

exacerbated by a lack of critical care guidelines that cater to rural areas[57]. Such geographical inequity in ICU critical care could be detrimental and of particular concern during a critical care crisis (e.g. COVID-19)[1].

However, critical care delivery is heterogenous and can be provided in advanced or basic forms, depending on resource availability. Critical care can involve high tech and high-cost equipment that requires highly trained staff (e.g. mechanical ventilation) and is more often found in the ICU. Investing in this level of critical care is therefore an expensive policy decision and may explain the lack of ICU critical care availability in rural areas in Tanzania. On the other hand, investing in adequately resourced critical care outside of ICUs can potentially save lives and reduce the case load that requires ICU care [58]. We found that non-ICU critical care was reported to be given in wards and theatres[43] across rural and urban areas.

A key finding of the literature review was that studies did not provide breakdowns of costs by the level of care provided, complexity of care, resource intensity or quality of provision . Importantly, for our study, they also did not provide breakdowns between the care for a specific condition relative to the care for the critical illness. We were therefore only able to characterise critical care in Tanzania by where the care was provided within a hospital i.e. ICU vs non-ICU delivered critical care. This is of importance as care for the critically ill takes many forms/interventions depending not only on whether it is ICU based but also by the complexity, the type of provider, the quality of care as well as the primary diagnosis. Characterising the minimum needs for the care of a critically ill patient and understanding how critical care delivery varies across these parameters will facilitate our understanding of what elements of critical care

For peer review only - http://bmjopen.bmj.com/site/about/guidelines.xhtml

are currently available as well as recommendations for the progressive improvement of critical care in all facilities. Previous research suggests that basic critical care, which effectively identifies and treats all critically ill patients, should be available and provided in all settings within a hospital (e.g. in wards) across rural and urban settings [59,60].

Recently, some of this paper's authors have proposed the concept of Essential Emergency and Critical Care (EECC) across LMICs which could be the future of low-cost critical care. EECC is pragmatic and low-cost care of critical illness that does not need to be reliant on sophisticated technology, and have a primary focus on simple, effective actions that have large potential impact on the population[58] and the components of have been specified in a large global consensus[12]. Some of the critical care interventions that have been identified as part of EECC[12] that can be provided in general wards include; identification of critical illness using vital signs based triage, caring for blocked or threatened airway for example suction for secretions that are obstructing the airway and insertion of an oropharyngeal airway, caring for hypoxia or respiratory distress, threatened circulation or shock and reduced level of consciousness, see Schell et al[12] for further details.

Given the lack of availability and access of critical care across Tanzania, such novel approaches may provide a building block to developing a fully resourced, equitable critical care system[61]. However, decision makers require further evidence on the optimal design, costs, budget impact and cost-effectiveness of such critical care interventions in rural and urban systems.

Complexity of resources within critical care

For peer review only - http://bmjopen.bmj.com/site/about/guidelines.xhtml

At the hospital level, we found that many studies identified resources that were used in the provision of services that included a critical care component but only a few studies went further to report on the availability of the resources at the facility (e.g. equipment, consumables and human resources). Of the critical care components, resource availability of oxygen was explored in four studies[38,41,43,62]. Other components explored were personal protective equipment (two studies)[38,41] and availability of utilities like running water and electricity (two studies)[38,41].

While oxygen is one of the essential resources required in the provision of critical care, many hospitals still lack basic equipment for its storage and delivery[63]. Our findings show that even when oxygen is available, the equipment and consumables required to facilitate provision of oxygen therapy can be missing[43,62]. This leaves a considerable proportion of critically ill patients in need of medical oxygen without the necessary treatment, incurring unnecessary deaths and disability.

Many reasons exist as to why availability and supply of oxygen is limited in settings such as Tanzania. For example, supply and scaling of medical oxygen is a complex process and is often controlled though monopoly suppliers. Regulation and high investment costs create barriers for new firms to enter the market and easily scale up supply[64]. In Tanzania, medical oxygen supply is controlled centrally by a quasi-governmental monopoly organisation[65]. On the demand side, there is limited funding for medical oxygen with procurement decentralised to individual hospitals with constraint budgets[63]. Alternatives, such as oxygen concentrators and hospital plants for the local production of oxygen, are affected by the need for a constant supply of

For peer review only - http://bmjopen.bmj.com/site/about/guidelines.xhtml

electricity, compounded by challenges with maintaining equipment. In Tanzania, similar to many LMICs, approx. 50% of hospitals are without a constant oxygen supply[41] and there is limited local capacity to maintain and repair oxygen concentrators like Tanzania.

In addition to oxygen equipment, we found variability in availability of personal protective equipment (PPE), and human resources across studies. Some PPE especially gloves, were available in most hospitals, while others, such as eye protection, remained unavailable, which is in line with previous research[66]. In terms of human resources, evidence suggests that only 20% of hospitals have at least one health worker trained in adult critical care which includes administering complex oxygen related equipment [38,57,59]. Technical assistance, training of health workers, and improved processes for non ICU based care, development of context-specific critical care guidelines, building capacity in equipment maintenance and biomedical engineering in facilities, and support to medical oxygen infrastructure development should be prioritised by governments and in funding applications to multilaterals and technical assistance agencies [38,67,68].

Costs of critical care

Costing of critical care is vital in eliciting potential budget constraints or impacts, assessing cost-effectiveness, providing insight into the economic burden of disease and can be useful for understanding the resources incurred by health systems, other payers, and patients. We find that no study reported or calculated an average cost or cost per patient day for the provision of critical services. On the other hand, many studies reported individual costs of resources associated with provision of critical care and it was possible to identify the cost of specific resources needed in

For peer review only - http://bmjopen.bmj.com/site/about/guidelines.xhtml

critical care provision (i.e. human resource cost, services, medication, and other consumables). Such limited evidence could be due to no formal or standardised definition of critical care existing thereby reducing specificity when costing critical care provisioning. Another possibility may be that patients who require critical care are heterogenous. The underlying demographics, epidemiology, risk factors, patient pathway and interventions administered are also heterogenous thereby making aggregation and estimates of average cost challenging. This is further confirmed by the data extracted in this review which showed that reporting of costs within eligible studies varied by defined services provided, breakdowns of costs and inputs, as well as the cost unit, thereby making comparisons or synthesising information across studies difficult. There is an urgent need to understand the resources required and their cost implications to inform the planning of improved critical care, globally.

Almost 50% of the Tanzanian population have a daily consumption of less than 1.91USD[69]. At the same time, the share of out-of-pocket payments in total health expenditure was 22% in 2019[70]. Considering the cost of high-tech critical care inputs, it is likely that critical care exposures patients to considerable out of pocket expenses resulting in catastrophic expenditure for low-income households that seek hospital care for critically ill family members. Countries should consider implementing EECC which could reduce the likely cost of critical care and therefore the financial burden on patients' households in their respective settings.

Lessons learned for other low resource settings/LMICs

The variability in critical care provision, availability of critical care resources, prices and costs are not unique to Tanzania. The heterogeneity in critical care is well documented across the

For peer review only - http://bmjopen.bmj.com/site/about/guidelines.xhtml

globe[71]. Variability in costs is also well documented across health facilities in LMICs[5]. The research and data gaps in these areas– understanding resource availability in critical care across different settings (both ICU and non ICU) - is less well documented but it is likely that the Tanzania's data gaps are replicated in many LMICs. LMICs will require research and data collection efforts in ascertaining these resource gaps and costing of critical care to inform budgets and planning.

Limitations

There are a number of inherent limitations with this systematic review. Firstly, we tailored our search strategy to account for the lack of standardised or formal definitions for critical illness, critical care provision and ICUs [59] by including specific terms for conditions likely to lead to critical illness and identified in consultation with clinicians on the authorship team. Here, we may have introduced a personal bias by focussing on specific conditions and excluding others. Secondly, cost and resource use in an African context are traditionally poorly documented and can fluctuate due to market dynamics and resource availability. While this systematic review provides key insights into peer reviewed published information on the resource use, availability and costs of critical care in Tanzania, researchers must update a previous nationally representative facility survey conducted in 2012 extending it to include an understanding of the costs of critical care provision that includes patient level data on resource use. Finally, we opted to only include articles in English but believe the extent and effects of language bias are diminished recently because of the shift towards publication of studies in English[72].

For peer review only - http://bmjopen.bmj.com/site/about/guidelines.xhtml

Conclusion

Critical care is essential in the prevention of mortality among critically ill patients, yet limited evidence exists on the costs and cost-effectiveness of scaling up critical care thereby hindering informed planning for investments in key life-saving interventions. We further find that ICU based critical care provision is currently only provided in urban areas in Tanzania and there is an urgent need to assess the quality of non-ICU based critical care implemented to address the gap in critical care provision in rural and urban areas. Evidence on resources used and potential costs in critical care is limited, and, along with a specific focus on oxygen supply and delivery which is a key resource in critical care, requires further research. Improving the evidence base on the costs, utilisation, cost-effectiveness and equity impacts of different strategies for critical care services across rural and urban areas is needed to help prevent unnecessary deaths caused by critical illness in Tanzania.

For peer review only

Authors' contributions

Below is the detailed breakdown of their contribution.

Concept and design: JK, HS, LG, AK, PT, OS, TB

Development of first draft: JK, HS, LG

Acquisition of data: JK, HS, LG, PT, AK, KK, SG

Analysis and interpretation of data: JK, HS, LG, PT, SG, OS, KK, TB

Drafting of the manuscript: JK, HS, LG

Critical revision of paper for important intellectual content: TB, OS, LG, PT, SG, KK, AK

Statistical analysis: -

For peer review only - http://bmjopen.bmj.com/site/about/guidelines.xhtml

Provision of study materials:

Obtaining funding: TB, OS

Administrative and technical support:

Supervision: LG, HS

Competing interests

Authors declare no competing interests and the funders did not in any way influence the development of the development of this study.

TB declares personal fees from UNICEF, the World Bank, USAID and the Wellcome Trust, all outside the submitted work.

Funding statement

This work was supported by the Wellcome Trust [221571/Z/20/Z], as part of the 'Innovation in low-and middle-income countries' Flagship.

Data sharing statement

Supporting data is available on request.

Figures

Figure 1: PRISMA Chart

Legend: The PRISMA diagram shows the details the search and selection process applied during our systematic literature search and review.

References

1. Baker T, Otto Schell C, Brun Petersen D, et al. Essential care of critical illness must not be forgotten in the COVID-19 pandemic. *Lancet*. 2020;395:1253-1254.

For peer review only - http://bmjopen.bmj.com/site/about/guidelines.xhtml

doi:10.1016/S0140-6736(20)30793-5

2. Barasa E, Kairu A, Nganga W, et al. Examining Unit Costs for COVID-19 Case Management in Kenya. *medRxiv*. Published online October 13, 2020:2020.10.08.20209684. doi:10.1101/2020.10.08.20209684

3. Sen-Crowe B, Sutherland M, McKenney M, Elkbuli A. A Closer Look Into Global Hospital Beds Capacity and Resource Shortages During the COVID-19 Pandemic. *J Surg Res*. 2021;260:56. doi:10.1016/J.JSS.2020.11.062

4. Adhikari NKJ, Fowler RA, Bhagwanjee S, Rubenfeld GD. Critical care and the global burden of critical illness in adults. *Lancet*. 2010;376(9749):1339-1346. doi:10.1016/S0140-6736(10)60446-1

5. Reynolds TA, Sawe H, Rubiano AM, Shin S Do, Wallis L, Mock CN. Strengthening Health Systems to Provide Emergency Care. In: Jamison DT, Gelband H, Horton S, et al., eds. *Disease Control Priorities, Third Edition (Volume 9): Improving Health and Reducing Poverty*. 3rd ed. The International Bank for Reconstruction and Development / The World Bank; 2017:247-265. doi:10.1596/978-1-4648-0527-1_CH13

6. Ayebale ET, Kassebaum NJ, Roche AM, Biccard BM. Africa's critical care capacity before COVID-19. *South African J Anaesth Analg*. 2020;26(3):162-164. doi:10.36303/SAJAA.2020.26.3.2431

7. Kayambankadzanja RK, Schell CO, Wärnberg MG, et al. Towards definitions of critical illness and critical care using concept analysis. *medRxiv*. Published online January 10, 2022:2022.01.09.22268917. doi:10.1101/2022.01.09.22268917

For peer review only - http://bmjopen.bmj.com/site/about/guidelines.xhtml

8. National Institute for Health and Care Excellence (NICE). What “critical care” means | Information for the public | Rehabilitation after critical illness in adults | Guidance | NICE. Published March 25, 2009. Accessed November 1, 2020. https://www.nice.org.uk/guidance/cg83/ifp/chapter/What-critical-care-means

9. Zimmerman JE, Kramer AA, Knaus WA. Changes in hospital mortality for United States intensive care unit admissions from 1988 to 2012. *Crit Care*. 2013;17(2):R81. doi:10.1186/cc12695

10. Turner HC, Van Hao N, Yacoub S, et al. Achieving affordable critical care in low-income and middle-income countries Commentary Handling editor Seye Abimbola. *BMJ Glob Heal*. 2019;4:1675. doi:10.1136/bmjgh-2019-001675

11. Schell CO, Gerdin Wärnberg M, Hvarfner A, et al. The global need for essential emergency and critical care. *Crit Care*. 2018;22(1):1-5. doi:10.1186/s13054-018-2219-2

12. Schell CO, Khalid K, Wharton-Smith A, et al. Essential Emergency and Critical Care: a consensus among global clinical experts. *BMJ Glob Heal*. 2021;6(9):e006585. doi:10.1136/BMJGH-2021-006585

13. Kayambankadzanja RK, Schell CO, Namboya F, et al. The prevalence and outcomes of sepsis in adult patients in two hospitals in Malawi. *Am J Trop Med Hyg*. 2020;102(4):896-901. doi:10.4269/ajtmh.19-0320

14. Kayambankadzanja RK, Schell CO, Mbingwani I, Mndolo SK, Castegren M, Baker T. Unmet need of essential treatments for critical illness in Malawi. Chen T-H, ed. *PLoS One*. 2021;16(9):e0256361. doi:10.1371/JOURNAL.PONE.0256361

For peer review only - http://bmjopen.bmj.com/site/about/guidelines.xhtml

15. King C, Banda M, Bar-Zeev N, et al. Care-seeking patterns amongst suspected paediatric pneumonia deaths in rural Malawi. *Gates Open Res 2021 4178*. 2021;4:178. doi:10.12688/gatesopenres.13208.2

16. Hillary C, Tom P, P. K, A.M. M, G. S, D. M. A survey of emergency and surgical services in the United Republic of Tanzania. *Acad Emerg Med*. 2011;18(5 SUPPL. 1):S242. doi:http://dx.doi.org/10.1111/j.1553-2712.2011.01073.x

17. World Health Organisation. *Strengthening the Health Systems Response to COVID-19: Creating Surge Capacity for Acute and Intensive Care* .; 2020. Accessed September 21, 2021. http://apps.who.int/bookorders.

18. World Bank. World Bank facilitates immediate support and resources for the COVID-19 (Coronavirus) emergency in Bolivia. Published April 2, 2020. Accessed December 16, 2020. https://www.worldbank.org/en/news/press-release/2020/04/02/el-banco-mundial-facilita-apoyo-y-recursos-inmediatos-para-atender-la-emergencia-por-el-covid-19-coronavirus-en-bolivia

19. The World Bank. *The World Bank Annual Report 2020: Supporting Countries in Unprecedented Time*.; 2020. Accessed December 16, 2020. https://www.worldbank.org/en/about/annual-report/covid-response

20. Kazibwe J, Shah HA, Kuwawenaruwa A, et al. Resource availability, utilisation and cost in the provision of critical care in Tanzania: a protocol for a systematic review. *BMJ Open*. 2021;11(8):e050881. doi:10.1136/BMJOPEN-2021-050881

21. Page MJ, McKenzie JE, Bossuyt PM, et al. The PRISMA 2020 statement: an updated guideline for reporting systematic reviews. *BMJ*. 2021;372. doi:10.1136/BMJ.N71

For peer review only - http://bmjopen.bmj.com/site/about/guidelines.xhtml

22. Moher D, Liberati A, Tetzlaff J, et al. Preferred reporting items for systematic reviews and meta-analyses: The PRISMA statement (Chinese edition). *J Chinese Integr Med*. 2009;7(9):889-896. doi:10.3736/jcim20090918

23. Savilaakso S, Garcia C, Garcia-Ulloa J, et al. Systematic review of effects on biodiversity from oil palm production. *Environ Evid*. 2014;3(1):4. doi:10.1186/2047-2382-3-4

24. Abbafati C, Machado DB, Cislaghi B, et al. Global burden of 369 diseases and injuries in 204 countries and territories, 1990–2019: a systematic analysis for the Global Burden of Disease Study 2019. *Lancet*. 2020;396(10258):1204-1222. doi:10.1016/S0140-6736(20)30925-9

25. Kazibwe J, Shah HA, Kuwawenaruwa A, et al. Resource availability, utilization and cost in the provision of critical care in Tanzania: A protocol for a systematic review. *BMJ Open*. Published online 2021.

26. Ouzzani M, Hammady H, Fedorowicz Z, Elmagarmid A. Rayyan-a web and mobile app for systematic reviews. *Syst Rev*. 2016;5(1). doi:10.1186/s13643-016-0384-4

27. World bank. GDP deflator (base year varies by country) - Tanzania . Accessed June 2, 2021. https://data.worldbank.org/indicator/NY.GDP.DEFL.ZS?locations=TZ

28. Vassall A, Sweeney S, Kahn JG, et al. *Reference Case for Estimating the Costs of Global Health Services and Interventions*.

29. Adawiyah R al, Saweri OPM, Boettiger DC, et al. The costs of scaling up HIV and syphilis testing in low- and middle-income countries: a systematic review. *Health Policy Plan*. 2021;2021:1-16. doi:10.1093/heapol/czab030

For peer review only - http://bmjopen.bmj.com/site/about/guidelines.xhtml

30. Nicks BA, Sawe HR, Juma AM, Reynolds TA. The state of emergency medicine in the United Republic of Tanzania. *African J Emerg Med*. 2012;2(3):97-102. doi:10.1016/j.afjem.2012.06.002

31. Sylvanus E, Sawe HR, Muhanuzi B, et al. Profile and outcome of patients with emergency complications of renal failure presenting to an urban emergency department of a tertiary hospital in Tanzania. *BMC Emerg Med*. 2019;19(1):11. doi:http://dx.doi.org/10.1186/s12873-019-0229-2

32. Engdahl Mtango S, Lugazia E, Baker U, Johansson Y, Baker T. Referral and admission to intensive care: A qualitative study of doctors' practices in a Tanzanian university hospital. Brenner S, ed. *PLoS One*. 2019;14(10):e0224355. doi:10.1371/journal.pone.0224355

33. Mkoka DA, Goicolea I, Kiwara A, Mwangu M, Hurtig AK. Availability of drugs and medical supplies for emergency obstetric care: Experience of health facility managers in a rural District of Tanzania. *BMC Pregnancy Childbirth*. 2014;14(1):1-10. doi:10.1186/1471-2393-14-108

34. Papali A, Adhikari NKJ, Diaz J V., et al. Infrastructure and organization of adult intensive care units in resource-limited settings. In: *Sepsis Management in Resource-Limited Settings*. Springer International Publishing; 2019:31-68. doi:10.1007/978-3-030-03143-5_3

35. Mwiga F, Monaghan M. Development of a difficulty airway trolley (DAT) at a tertiary referral hospital in northern Tanzania. *Trends Anaesth Crit Care*. 2020;30:e107-e108. doi:http://dx.doi.org/10.1016/j.tacc.2019.12.265

36. Sawe HR, Reynolds TA, Mfinanga JA, et al. The clinical presentation, utilization, and

For peer review only - http://bmjopen.bmj.com/site/about/guidelines.xhtml

outcome of individuals with sickle cell anaemia presenting to urban emergency department of a tertiary hospital in Tanzania. *BMC Hematol*. 2018;18(1):25. doi:http://dx.doi.org/10.1186/s12878-018-0122-3

37. Murthy S, Leligdowicz A, Adhikari NKJ. Intensive care unit capacity in low-income countries: A systematic review. *PLoS One*. 2015;10(1):116949. doi:http://dx.doi.org/10.1371/journal.pone.0116949

38. Baker T, Lugazia E, Eriksen J, Mwafongo V, Irestedt L, Konrad D. Emergency and critical care services in Tanzania: A survey of ten hospitals. *BMC Health Serv Res*. 2013;13(1):1-9. doi:10.1186/1472-6963-13-140

39. Rajaguru PP, Jusabani MA, Massawe H, Temu R, Sheth NP. Understanding surgical care delivery in Sub-Saharan Africa: a cross-sectional analysis of surgical volume, operations, and financing at a tertiary referral hospital in rural Tanzania. *Glob Heal Res policy*. 2019;4:30. doi:https://dx.doi.org/10.1186/s41256-019-0122-2

40. Reynolds TA, Mfinanga JA, Sawe HR, Runyon MR, Mwafongo V. Emergency care capacity in Africa: a clinical and educational initiative in Tanzania. *J Public Health Policy*. 2012;33(Supplement 1):S126-S137. doi:http://dx.doi.org/10.1057/jphp.2012.41

41. Penoyar T, Cohen H, Kibatala P, et al. Emergency and surgery services of primary hospitals in the United Republic of Tanzania. *BMJ Open*. 2012;2(1):e000369. doi:10.1136/bmjopen-2011-000369

42. Chalya PL, Gilyoma JM, Dass RM, et al. Trauma admissions to the Intensive care unit at a reference hospital in Northwestern Tanzania. *Scand J Trauma Resusc Emerg Med*. 2011;19:61. doi:10.1186/1757-7241-19-61

For peer review only

For peer review only - http://bmjopen.bmj.com/site/about/guidelines.xhtml

43. Staton CA, Msilanga D, Kiwango G, et al. A prospective registry evaluating the epidemiology and clinical care of traumatic brain injury patients presenting to a regional referral hospital in Moshi, Tanzania: challenges and the way forward. *Int J Inj Contr Saf Promot*. 2017;24(1):69-77. doi:10.1080/17457300.2015.1061562

44. Sawe HR, Reynolds TA, Mfinanga JA, et al. The clinical presentation, resource utilization, and outcomes of patients with sickle cell disease presenting to the ED of the muhimbili national Hospital in Dar es Salaam Tanzania. *Acad Emerg Med*. 2016;23(SUPPL. 1):S99. doi:http://dx.doi.org/10.1111/acem.12974

45. Rajaguru PP, Jusabani MA, Massawe H, Temu R, Sheth NP. Understanding surgical care delivery in Sub-Saharan Africa: a cross-sectional analysis of surgical volume, operations, and financing at a tertiary referral hospital in rural Tanzania. *Glob Heal Res Policy*. 2019;4(1):30. doi:10.1186/s41256-019-0122-2

46. Kimaro GD, Mfinanga S, Simms V, et al. The costs of providing antiretroviral therapy services to HIV-infected individuals presenting with advanced HIV disease at public health centres in Dar es Salaam, Tanzania: Findings from a randomised trial evaluating different health care strategies. *PLoS One*. 2017;12(2):e0171917. doi:http://dx.doi.org/10.1371/journal.pone.0171917

47. Sicuri E, Vieta A, Lindner L, Constenla D, Sauboin C. Economic costs of malaria in children in three Sub-Saharancountries: Ghana, Tanzania and Kenya. *Trop Med Int Heal*. 2011;16(SUPPL. 1):117. http://ovidsp.ovid.com/ovidweb.cgi?T=JS&PAGE=reference&D=emed12&NEWS=N&AN=70589204

For peer review only - http://bmjopen.bmj.com/site/about/guidelines.xhtml

48. Riewpaiboon A, Piatti M, Ley B, et al. Cost of illness due to typhoid fever in Pemba, Zanzibar, East Africa. *J Heal Popul Nutr*. 2014;32(3):377-385. http://www.jhpn.net/index.php/jhpn/article/view/2819/1043

49. James C, Bura M, Ensor T. The costs of delivering health services in Tanzania. Findings from a comprehensive costing analysis.

50. Chaudhury S, Arlington L, Brenan S, et al. Cost analysis of large-scale implementation of the "Helping Babies Breathe" newborn resuscitation-training program in Tanzania. *BMC Health Serv Res*. 2016;16(1):681. http://ovidsp.ovid.com/ovidweb.cgi?T=JS&PAGE=reference&D=emed17&NEWS=N&AN=618644170

51. Guerriero C, Cairns J, Jayaraman S, Roberts I, Perel P, Shakur H. Giving tranexamic acid to reduce surgical bleeding in sub-Saharan Africa: An economic evaluation. *Cost Eff Resour Alloc*. 2010;8:1. doi:http://dx.doi.org/10.1186/1478-7547-8-1

52. Penno EC, Baird SJ, Crump JA. Cost effectiveness of surveillance for bloodstream infections for sepsis management in low resource settings. *Am J Trop Med Hyg*. 2015;93(4):850-860. http://www.ajtmh.org/content/91/5_Suppl_1/74.full.pdf+html

53. Shayo GA, Chitama D, Moshiro C, Aboud S, Bakari M, Mugusi F. Cost-Effectiveness of isoniazid preventive therapy among HIV-infected patients clinicaly screened for latent tuberculosis infection in Dar es Salaam, Tanzania: A prospective Cohort study. *BMC Public Health*. 2017;18(1):35. doi:http://dx.doi.org/10.1186/s12889-017-4597-9

54. Mengistu T, Berruti A, Krivelyova A, Swor M, Waite R, Maro G. Cost of providing emergency obstetric care in Tanzania's Kigoma region. *Int J Health Plann Manage*.

For peer review only - http://bmjopen.bmj.com/site/about/guidelines.xhtml

2019;34(4):e1510-e1519. doi:http://dx.doi.org/10.1002/hpm.2820

55. Baynes C, Yegon E, Kimaro G, Lusiola G, Kahwa J. The unit and scale-up cost of postabortion care in Tanzania. *Spec Issue Sav women's lives through Emerg Obstet care Volunt Fam planning*. 2019;7(Suppl. 2):S327-S341. doi:http://dx.doi.org/10.9745/GHSP-D-19-00035

56. The World Bank Group. Rural population (% of total population) - Tanzania | Data. 2021. Accessed July 14, 2021. https://data.worldbank.org/indicator/SP.RUR.TOTL.ZS?locations=TZ

57. Schultz MJ, Dunser MW, Dondorp AM, et al. Current challenges in the management of sepsis in ICUs in resource-poor settings and suggestions for the future. *Intensive Care Med*. 2017;43:612-624. doi:10.1007/s00134-017-4750-z

58. Ranjit S, Kissoon N. Challenges and Solutions in translating sepsis guidelines into practice in resource-limited settings. *Transl Paediatr*. Published online 2021. Accessed August 10, 2021. https://tp.amegroups.com/article/viewFile/66320/pdf

59. Baker T, Khalid K, Acicbe O, McGloughlin S, Amin P. Critical care of tropical disease in low income countries: Report from the Task Force on Tropical Diseases by the World Federation of Societies of Intensive and Critical Care Medicine. *J Crit Care*. 2017;42:351-354. doi:10.1016/J.JCRC.2017.11.028

60. Bion J, Brown C, Gomersall C, Boulanger C, Isherwood P, Schulman D. Society of Critical Care Medicine 50th Anniversary Review Series: Critical Care Education. *Crit Care Med*. Published online 2021:1241-1253. doi:10.1097/CCM.0000000000005130

For peer review only - http://bmjopen.bmj.com/site/about/guidelines.xhtml

61. Kairu A, Were V, Isaaka L, Agweyu A, Aketch S, Barasa E. Modelling the cost-effectiveness of essential and advanced critical care for COVID-19 patients in Kenya. *medRxiv*. Published online August 24, 2021:2021.08.16.21261894. doi:10.1101/2021.08.16.21261894

62. Zimmerman A, Fox S, Griffin R, et al. An analysis of emergency care delays experienced by traumatic brain injury patients presenting to a regional referral hospital in a low-income country. *PLoS One*. 2020;15(10 October 2020):e0240528. doi:http://dx.doi.org/10.1371/journal.pone.0240528

63. Kazibwe Joseph, Baker Peter, Baker Tim, et al. We Can Stop Preventable COVID Deaths by Urgently Prioritising Medical Oxygen and Essential Critical Care | Center For Global Development. Center for Global Development. Published February 9, 2021. Accessed August 2, 2021. https://www.cgdev.org/blog/we-can-stop-preventable-covid-deaths-urgently-prioritising-medical-oxygen-and-essential

64. Davies M, Onwuzoo A, Mednick S. Fighting for breath: how the medical oxygen industry is failing African hospitals . The Guardian. Published August 10, 2020. Accessed August 9, 2022. https://www.theguardian.com/global-development/2020/aug/10/fighting-for-breath-how-the-medical-oxygen-industry-is-failing-african-hospitals

65. Tanzania Medicines and Medical Devices Authority. Guidelines for regulating medical gases. Accessed September 16, 2021. https://www.tmda.go.tz/uploads/publications/en1597391138-GUIDELINES FOR MEDICAL GASES - Final (1)-SK.pdf

66. Powell-Jackson T, King JJC, Makungu C, et al. Infection prevention and control

For peer review only

compliance in Tanzanian outpatient facilities: a cross-sectional study with implications for the control of COVID-19. *Lancet Glob Heal*. 2020;8(6):e780-e789. doi:10.1016/S2214-109X(20)30222-9

67. Murthy S, Adhikari NK. ATS REPORTS Global Health Care of the Critically Ill in Low-Resource Settings. *Ann Am Thorac Soc*. 2013;10(5):509-513. doi:10.1513/AnnalsATS.201307-246OT

68. Diaz J V., Riviello ED, Papali A, Adhikari NKJ, Ferreira JC. Global critical care: Moving forward in resource-limited settings. *Ann Glob Heal*. 2019;85(1):3-4. doi:10.5334/aogh.2413

69. World Bank Group. Poverty & Equity Brief Sub Saharan – Tanzania. Published April 2020. Accessed August 18, 2022. https://databankfiles.worldbank.org/data/download/poverty/33EF03BB-9722-4AE2-ABC7-AA2972D68AFE/Global_POVEQ_TZA.pdf

70. The World Bank Group. Out-of-pocket expenditure (% of current health expenditure) - Tanzania. Accessed July 26, 2022. https://data.worldbank.org/indicator/SH.XPD.OOPC.CH.ZS?locations=TZ

71. Wilcox ME, Vaughan K, Chong CAKY, Neumann PJ, Bell CM. Cost-Effectiveness Studies in the ICU: A Systematic Review∗. *Crit Care Med*. 2019;47(8):1011-1017. doi:10.1097/CCM.0000000000003768

72. Cochrane Reviews. Cochrane Handbook: 10.2.2.4 Language bias. In: *Cochrane*. Accessed September 16, 2021. https://handbook-5-1.cochrane.org/chapter_10/10_2_2_4_language_bias.htm

For peer review only - http://bmjopen.bmj.com/site/about/guidelines.xhtml

For peer review only

For peer review only - http://bmjopen.bmj.com/site/about/guidelines.xhtml

Figures

Figure 1: PRISMA Chart

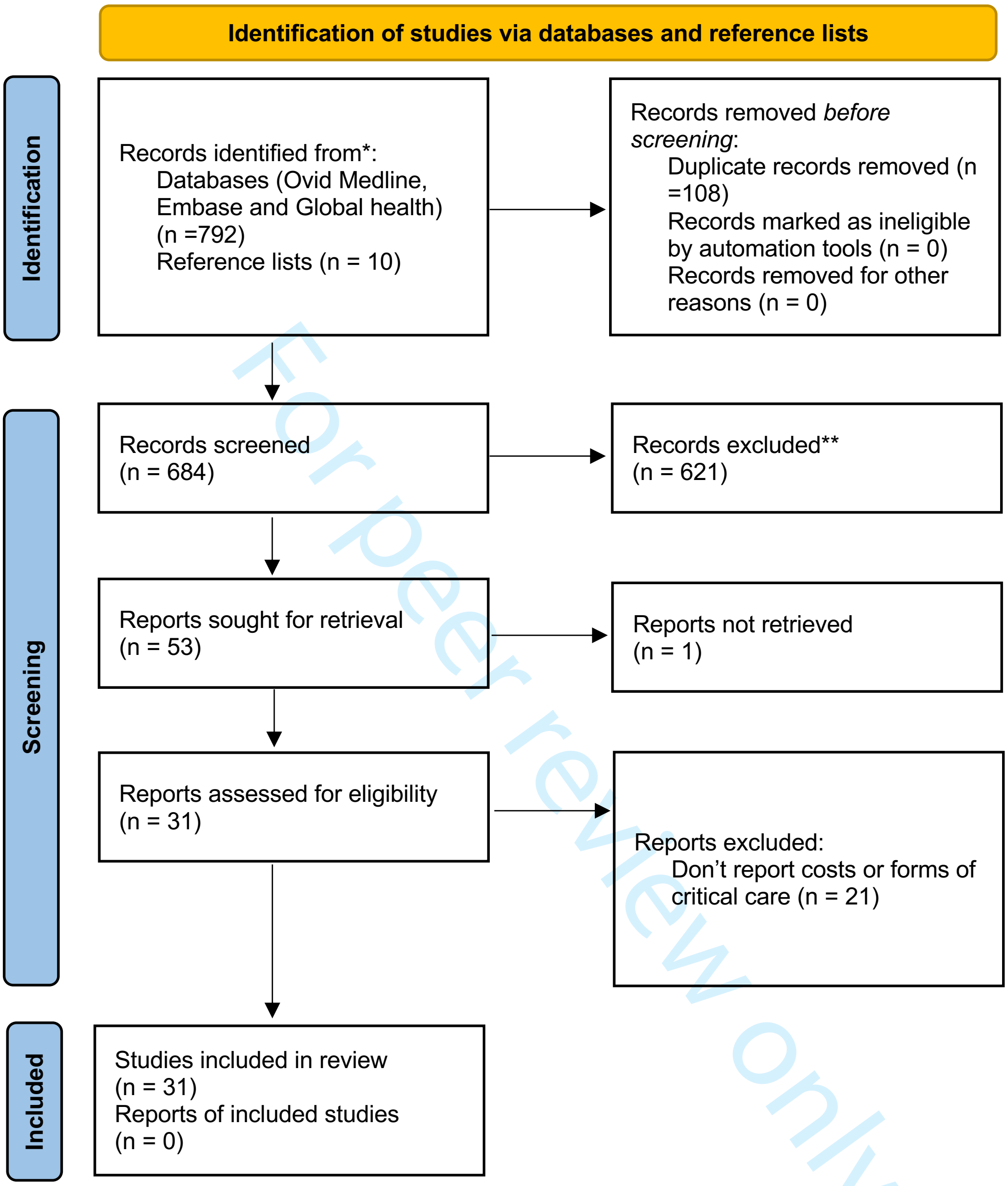

Legend: The PRISMA diagram shows the details the search and selection process applied during our systematic literature search and review.

Based on Page MJ, McKenzie JE, Bossuyt PM, Boutron I, Hoffmann TC, Mulrow CD, et al. The PRISMA 2020 statement: an updated guideline for reporting systematic reviews. BMJ 2021;372:n71. doi: 10.1136/bmj.n71

SUPPLEMENTARY MATERIAL

Supplementary 1: Key concepts and search terms identified for the search.

Databases: Embase, Medline and Global Health
Search platform: Ovid

Database: Embase Classic+Embase <1947 to 2020 November 18> , Global Health <1973 to 2020 Week 45>, Ovid MEDLINE(R) ALL <1946 to November 17, 2020>
Search Strategy:
----
1 exp intensive care/ or hospital care.ab,ti. or artificial ventilation/ or early goal-directed therapy/ or intensive care nursing/ or newborn intensive care/ or patient monitoring/ or pediatric advanced life support.ab,ti. or emergency.ab,ti. or urgent care.ab,ti. (1538827)
2 (hospital patient or inpatient).ab,ti. (248658)
3 exp hospital patient/ (188072)
4 exp "health care cost"/ or "cost"/ or health economics/ or "drug cost"/ or health care financing/ or "hospital cost"/ or nursing cost.ab,ti. or cost.ab,ti. or costs.ab,ti. (1697788)
5 exp tanzania/ or tanzania.ab,ti. (45585)
6 exp Health Care Costs/ or exp Health Expenditures/ or (cost* or ((health care or health-care or healthcare or medical) adj3 expenditure*) or expense* or hospital resources).ti,ab. or (((resource* or "health service") adj3 ("use" or utilisation or utilization)) or ((financial or economic) and (burden or impact or consequence)) or spending or out of pocket or out-of-pocket or OOP or insurance or absenteeism or (productivity and (loss or lost)) or "economic modelling").ab,ti. (2248416)
7 artificial ventilation.ab,ti. (7728)
8 (malaria or tb or tuberculosis or diagnostics or laboratory or lab or hiv).ab,ti. (3253274)
9 (general care or general ward care or general inpatient care or hospital care).ab,ti. (27507)
10 1 or 2 or 3 or 7 or 8 or 9 (4988953)
11 (economic evaluation or cost effective).ab,ti. (264378)
12 4 or 6 or 11 (2320354)
13 5 and 10 and 12 (1905)
14 limit 13 to yr="2010 -Current" (1295)
15 remove duplicates from 14 (792)

For peer review only - http://bmjopen.bmj.com/site/about/guidelines.xhtml

Google Scholar

"Intensive care" OR "Critical care" OR "Critical illness" OR "Resource use" AND "Tanzania"

Supplementary 2: Inclusion and exclusion criteria

Inclusion criterion
For a study to be included it should fulfil the following criterion

- Published in English in a peer reviewed journal
- Reports forms or types of critical care offered, critical care services offered and or costs and resources used in the provision of care in Tanzania
- Includes costs from a provider perspective
- Articles published from 2010

Exclusion criterion
Studies will be excluded in case of the following;

- Published in any other languages except English
- Studies carried out outside Tanzania
- Studies with costs estimated from patient perspective
- Studies without full text versions
- Studies published before 2010

Supplementary 3: Study Characteristics (N=31)]

For peer review only - http://bmjopen.bmj.com/site/about/guidelines.xhtml

Article	Disease area	Intervention	Study Type	Purpose Type	Geographical area covered	Hospital Type	Facility/hospital Level	Source of data
**Studies reporting on costs**								
Baynes et al 2019	Obstetrics	Providing care to women that have had an abortion	Cross sectional	Cost Analysis	Mwanza, Arusha and Zanzibar	NGO & Public	All	Records and interviews
Chris et al 2013	General	Health services	Cross sectional	Cost Analysis	National	All	All	Survey
Kabadi et al 2013	Stroke	Providing stroke care	Longitudinal	Cost Analysis	Mbeya and Dar es Salaam	NGO & Public	Tertiary referral	Survey and project data
Mengistu et al 2019	Direct obstetric complications	Emergency Obstetric and Neonatal Care	Cross sectional	Cost Analysis	Kigoma	Public	Tertiary, district hospital and primary care	Programme & hospital data

For peer review only - http://bmjopen.bmj.com/site/about/guidelines.xhtml

Article	Disease area	Intervention	Study Type	Purpose Type	Geographical area covered	Hospital Type	Facility/hospital Level	Source of data
Mosha et al 2010	Malaria	Diagnostic tools and drugs	Cross sectional	Cost analysis	Kilimanjaro region and Korogwe District, Tanga region	Public	District and dispensaries	Survey
Riewpaiboon et al 2014	Typhoid	Typhoid treatment/care	Longitudinal	Cost Analysis	Pemba and Zanzibar	Public	District hospital	Record review and interviews
Sicuri et al 2011	Malaria	Malaria treatment/care	Longitudinal	Cost Analysis	Not specified	Not specified	Not specified	Survey
Chaudhury et al 2016	Neonatal care	Birth attendants training on	Cross sectional	Cost Analysis	Mbeya	NGO & Public	All	Survey

For peer review only - http://bmjopen.bmj.com/site/about/guidelines.xhtml

**Article**	**Disease area**	**Intervention**	**Study Type**	**Purpose Type**	**Geographical area covered**	**Hospital Type**	**Facility/hospital Level**	**Source of data**
		neonatal resuscitation						
Penno et al 2015	Antimicrobial resistance	Surveillance for Bloodstream Infections for Sepsis Management in low resource setting	Modelling	Economic evaluation	Northern Tanzania	Not specified	Not specified	Laboratory records
Githang'a et 2020	Oncology	Oncology treatment: e.g. chemotherapy, off site	Cross-sectional	Economic evaluation	Mwanza	NGO	Tertiary referral	Hospital data & interviews

For peer review only - http://bmjopen.bmj.com/site/about/guidelines.xhtml

Article	Disease area	Intervention	Study Type	Purpose Type	Geographical area covered	Hospital Type	Facility/hospital Level	Source of data
		radiotherapy, surgery						
Guerriero et al 2010	Blood transfusion	Use of Tranexamic acid as a replacement for blood transfusion	Modelling	Economic evaluation	National	Not specified	Not specified	Literature

For peer review only - http://bmjopen.bmj.com/site/about/guidelines.xhtml

Article	Disease area	Intervention	Study Type	Purpose Type	Geographical area covered	Hospital Type	Facility/hospital Level	Source of data
Kimaro et al 2017	Advanced HIV	Providing antiretroviral therapy services to HIV-infected individuals presenting with advanced HIV disease	Cohort	Economic evaluation	Dar es Salaam	Public	Primary health center	Patient survey, health care worker diaries
Phillips et al 2015	Malaria	Malaria Rapid Diagnostic test (mRDT)	Modelling	Economic evaluation	National	All	All	Local program/ records

For peer review only - http://bmjopen.bmj.com/site/about/guidelines.xhtml

Article	Disease area	Intervention	Study Type	Purpose Type	Geographical area covered	Hospital Type	Facility/hospital Level	Source of data
Shayo et al 2017	Tuberculosis	Isoniazid preventive therapy	Cohort	Economic evaluation	Dar es Salaam	NGO, Public and private	Tertiary and primary care	Records and interviews
Wilson et al 2020	Neonatal care	Birth attendants training on neonatal resuscitation (59 participants)	Cross sectional	Financial planning	Zanzibar	Not specified	Not specified	Survey
**Studies reporting on forms and resource availability in critical care**								
Baker et al 2011	Critical care	Emergency and critical care	Cross sectional	Others (structure and resources	National	All	Tertiary, district, and primary care	Survey

For peer review only - http://bmjopen.bmj.com/site/about/guidelines.xhtml

Article	Disease area	Intervention	Study Type	Purpose Type	Geographical area covered	Hospital Type	Facility/hospital Level	Source of data
				available for emergency and critical care)				
Chalya et al 2011	Trauma	Trauma care	Cross sectional	Others (describe patient characteristics and health outcomes)	Mwanza	NGO	Referral hospital	Survey
Mkoka et al 2014	Obstetrics	Emergency obstetric care	Cross sectional	Others (availability	Kongwa	Public	Primary healthcare	Interviews

For peer review only - http://bmjopen.bmj.com/site/about/guidelines.xhtml

Article	Disease area	Intervention	Study Type	Purpose Type	Geographical area covered	Hospital Type	Facility/hospital Level	Source of data
				of medical supplies)				
Mtango et al 2019	Critical care	Critical care	Cross sectional	Others (doctors' experiences of ICU referral)	Dar es salaam	Public	Tertiary referral	Interviews
Murthy et al 2015	Critical care	N/A	Review	Others (estimate the current ICU capacity)	Ifakara, Dar es Salaam and Mwanza	NGO & Public	Tertiary referral	Literature
Mwandri et al 2020	Trauma	Baseline assessment of	Cross sectional	Others (assessing	Mbeya and Dar es Salaam	NGO & Public	Tertiary referral	Hospital registry data

For peer review only - http://bmjopen.bmj.com/site/about/guidelines.xhtml

Article	Disease area	Intervention	Study Type	Purpose Type	Geographical area covered	Hospital Type	Facility/hospital Level	Source of data
		trauma care provision		burden and care process)				
Mwiga & Monaghan 2020	Respiratory illness	Difficult airway trolley	Case study	Others (development of difficulty airway trolley)	Mbeya	NGO	Tertiary referral	Difficult airway trolley
Nicks et al 2012	Emergence medicine	N/A	Case study	Others (structure of health system and care processes)	National	All	All	Literature

For peer review only - http://bmjopen.bmj.com/site/about/guidelines.xhtml

Article	Disease area	Intervention	Study Type	Purpose Type	Geographical area covered	Hospital Type	Facility/hospital Level	Source of data
Papali et al 2019	Critical care	N/A	Review	Others (structure and organisation of adult ICU)	N/A	All	All	Literature
Penoyar et al 2012	Surgery	Surgical care	Cross sectional	Others (capacity of first referral hospitals)	National	All	District, regional and referral	Survey
Rajaguru et al 2019	Surgery	Surgery	Cross sectional	Others (operation and financing of a referral hospital)	Mbeya	NGO	Tertiary referral	Record review

For peer review only - http://bmjopen.bmj.com/site/about/guidelines.xhtml

Article	Disease area	Intervention	Study Type	Purpose Type	Geographical area covered	Hospital Type	Facility/hospital Level	Source of data
Reynolds et al 2012	General (particular injuries, pneumonia and psychosis)	Emergency care	Cross sectional	Others (Barriers to emergency care)	National	Public	Tertiary referral	Interview
Sawe et 2016	General	Emergency treatment	Cross sectional	Others (clinical presentation and resource utilization)	Dar es Salaam	Public	Tertiary referral	Hospital record
Staton et al 2017	Traumatic Brain Injury	Trauma care	Cross sectional	Others (describe	Kilimanjaro	NGO	Referral Hospital	Survey

For peer review only - http://bmjopen.bmj.com/site/about/guidelines.xhtml

Article	Disease area	Intervention	Study Type	Purpose Type	Geographical area covered	Hospital Type	Facility/hospital Level	Source of data
				quality of care for TBI)				
Sylvanus et al 2019	Renal failure	Renal failure treatment and management	Cohort	Others (renal failure case management)	Dar es Salaam	Public	Tertiary referral	Survey
Zimmerman et al 2020	Acute traumatic brain injury	Quantifying the effect on outcomes from delays in the care of trauma	Cross sectional	Others (association of hospital delays and poor outcomes)	Moshi	NGO	Tertiary referral	Hospital data

For peer review only - http://bmjopen.bmj.com/site/about/guidelines.xhtml

Supplementary 4: Availability of resources required for critical care provision

**Resources**	**Study**			
	Penoyar et al 2012 (N=40 hospitals)	*Staton et al 2017 (patients; N=893)*	*Zimmerman et al 2020 (patients; N=3209)*	*Baker et al 2013 (N=10 hospitals)*
Availability of oxygen when needed	42%	22.9%	20.1%	
Oxygen concentrators	75%			
Mask and tubing to connect to oxygen supply	46%			
Pulse oximetry	13%			70%
Oxygen cylinders	35%			
Mechanical ventilators				40%
Oropharyngeal airway-adult	42%			60%
Oropharyngeal airway-paediatric	21%			
Personal protective wear (gloves)	90%			90%
Personal protective wear (aprons)	81%			
Personal protective wear (face masks)	69%			
Personal protective wear (eye protection)	40%			
Consistent running water	37.5%			100%
Consistent running electricity	37.5%			50%
Staff trained in critical care				20%

Supplementary 5: Appraisal Checklist

For peer review only - http://bmjopen.bmj.com/site/about/guidelines.xhtml

Study ID	Author	#1 Question(s)	#2 Perspective	#3 Time horizon	#4 Relevant inputs	#5 Methods for quantities	#6 Data source(s)	#7 Sample size	#8 Discount rate	#9 Sensitivity analysis	#10 Costs reporting
1	Githang'a et 2020	✓	✓	(✓)	✓	✓	✓		✓	✓	✓
2	Mengistu et al 2019	✓	✓	✓	✓	✓	✓			X	✓
3	Wilson et al 2020	✓	✓	X	✓	✓	✓			X	✓
4	Kimaro et al 2017	✓	✓	✓	✓	✓	✓			✓	✓
5	Shayo et al 2017	✓	✓	✓	✓	✓	✓			✓	✓
6	Phillips et al 2015	✓	✓	✓	✓	✓	✓			X	✓
7	Penno et al 2015	✓	✓	X	✓	✓	✓			✓	✓
8	Kabadi et al 2013	✓	✓	✓	✓	✓	✓			X	✓
9	Sicuri et al 2011	✓	✓	X	✓	✓	✓			✓	✓

For peer review only

10	Guerriero et al 2010	✓	✓	X	✓	✓	✓			✓	✓
11	Baynes et al 2019	✓	✓	(✓)	✓	✓	✓			✓	✓
12	Riewpaiboon et al 2014	✓	✓	(✓)	✓	✓	✓			✓	✓
13	Sumona et al 2016	✓	✓	(✓)	✓	✓	✓			✓	✓
14	James et al 2013	✓	✓	✓	✓	✓	✓			X	✓

Key: ✓ addressed; (✓) partially addressed; X Not addressed

For peer review only

Supplementary 6: Cost of diagnostics

Study	Treatment/disease area	Description	Unit of output	Currency year	Unit cost Price in 2019 (USD)	Unit cost Price in 2019 (TZS)
Mengistu et al 2019	Emergency obstetric care	Laboratory	per programme	2013	9.72	22249.85
Riewpaiboon et al 2014	Typhoid fever	Blood count	Per test	2010	1.64	3751.89
Riewpaiboon et al 2014	Typhoid fever	Blood chemistry	Per test	2010	2.46	5627.84
Kabadi et al 2013	Stroke	Diagnostics for stroke (Blood, ECG, ECHO, CXR) - Rural area	Cost per patient	2005-2006	3.09	7065.07
Kabadi et al 2013	Stroke	CT Scan- rural area	Cost per patient	2005-2006	44.35	101473.18
Kabadi et al 2013	Stroke	Diagnostics for stroke (Blood, ECG, ECHO, CXR) - Urban area	Cost per patient	2005-2006	35.89	82117.17

For peer review only - http://bmjopen.bmj.com/site/about/guidelines.xhtml

Kabadi et al 2013	Stroke	CT Scan- urban area	Cost per patient	2005-2006	55.43	126841.48
Kabadi et al 2013	Stroke	Diagnostics for stroke (Blood, ECG, ECHO, CXR) - average	Cost per patient	2005-2006	13.34	30519.33
Kabadi et al 2013	Stroke	CT Scan- average	Cost per patient	2005-2006	47.81	109400.77
Baird et al 2014	Sepsis management	Integrated Management of Adolescent and Adult Illness assessment	Per test	2011	1.17	2677.59
Baird et al 2014	Sepsis management	Lab assessment for sepsis- Negative blood culture	Per test	2011	13.09	29952.25
Baird et al 2014	Sepsis management	Lab assessment for sepsis- positive blood culture	Per test	2011	23.00	52633.02
Baird et al 2014	Sepsis management	Lab assessment for sepsis- ID and susceptibilities positive blood culture	Per test	2011	82.55	188901.34
Phillips et al 2015	Malaria	Malaria rapid diagnostic test	Per test	2010	14.92	34142.23
Shayo et al 2017	TB	Sputum microscopy	Per test	2012	2.16	4945.33
Shayo et al 2017	TB	Suptum culture	Per test	2012	10.83	24778.14
Kimaro et al 2017	HIV - ART provision	CD4 Count test	Per test	2012	17.74	40592.88
Kimaro et al 2017	HIV - ART provision	Alanine aminotransferase (ALAT) test	Per test	2012	0.99	2266.61
Kimaro et al 2017	HIV - ART provision	creatinine test	Per test	2012	0.35	798.46
Kimaro et al 2017	HIV - ART provision	Haemoglobin test	Per test	2012	0.98	2240.85
Kimaro et al 2017	HIV - ART provision	Random blood glucose (RBG) test	Per test	2012	0.96	2189.34
Kimaro et al 2017	HIV - ART provision	Venereal disease reference laboratory (VDRL) test	Per test	2012	2.13	4868.05
Kimaro et al 2017	HIV - ART provision	Pregnancy test	Per test	2012	0.60	1365.12
Kimaro et al 2017	HIV - ART provision	Full blood count (FBC) test	Per test	2012	2.30	5254.41

For peer review only - http://bmjopen.bmj.com/site/about/guidelines.xhtml

Kimaro et al 2017	HIV - ART provision	Serum cryptococcal meningitis (CRAG) test	Per test	2012	4.46	10199.73
Kimaro et al 2017	HIV - ART provision	Xpert test	Per test	2012	21.47	49118.41
Kimaro et al 2017	HIV - ART provision	Collection and processing of cerebral spinal fluid	Per test	2012	18.34	41957.99
Kimaro et al 2017	HIV - ART provision	Chest X-ray	Per test	2012	3.58	8190.70
Kimaro et al 2017	HIV - ART provision	Smear microscopy test	Per test	2012	2.04	4662.00
Kimaro et al 2017	HIV - ART provision	Sputum culture (Lowenstein Jensen) for TB	Per test	2012	7.70	17617.72

Supplementary 7: Cost of human resources

Study	Description	Unit of output	Currency year	Unit cost Price in 2019 (USD)	Unit cost Price in 2019 (TZS)
Mengistu et al 2019	Total personnel for Emergency obstetric care	per patient	2013	103.01	235,711.74
Baynes et al 2019	Cost of Obstetrician/gynecologist on all complications	Per patient	2016	10.00	22,884.06
Baynes et al 2019	Cost of Anesthesiologist on all obstetric complications	Per patient	2016	0.00	0.00

For peer review only - http://bmjopen.bmj.com/site/about/guidelines.xhtml

Baynes et al 2019	Cost Anesthetist on all obstetric complications	Per patient	2016	2.23	5,112.40
Baynes et al 2019	Cost of Anesthetist Assistant on all obstetric complications	Per patient	2016	1.81	4,138.61
Baynes et al 2019	Cost of doctor/ medical officer on all obstetric complications	Per patient	2016	15.75	36,030.22
Baynes et al 2019	Cost of assistant medical officer on all obstetric complications	Per patient	2016	5.00	11,442.03
Baynes et al 2019	Cost of clinical officer on all obstetric complications	Per patient	2016	0.85	1,947.58
Baynes et al 2019	Cost of registered nurse or principal enrolled nurse on obstetric all complications	Per patient	2016	9.79	22,397.16
Baynes et al 2019	Cost of enrolled nurse or midwife on all obstetric complications	Per patient	2016	43.62	99,813.44
Baynes et al 2019	Cost of medical attendant on all obstetric complications	Per patient	2016	6.06	13,876.50
Baynes et al 2019	Cost of laboratory technician on all complications	Per patient	2016	3.19	7,303.42
Baynes et al 2019	Cost of laboratory assistant on all complications	Per patient	2016	1.70	3,895.16

For peer review only - http://bmjopen.bmj.com/site/about/guidelines.xhtml

Baynes et al 2019	Cost of Sonographer on all obstetric complications	Per patient	2016	2.87	6,573.08
Baynes et al 2019	Cost of pharmacist on all complications	Per patient	2016	2.55	5,842.74
Baynes et al 2019	Cost of drug dispenser on all complications	Per patient	2016	1.06	2,434.47
Baynes et al 2019	Average personnel costs at primary care level facilities for all complications	Per patient	2016	14.28	32,670.64
Baynes et al 2019	Average personnel care costs at district hospitals for all complications	Per patient	2016	8.97	20,522.62
Baynes et al 2019	Average personnel costs at Regional hospital for all complications	Per patient	2016	30.14	68,968.66
Baynes et al 2019	Average personnel costs for all complications across any level of hospital	Per patient	2016	19.16	43,844.88
Shayo et al 2017	Assistant medical officer	Monthly salary	2012	638.96	1,462,064.84
Shayo et al 2017	Nurse	Monthly salary	2012	313.34	716,994.91
Shayo et al 2017	Health Attendant	Monthly salary	2012	440.41	1,007,738.83
Shayo et al 2017	Assistant Nurse	Monthly salary	2012	338.60	774,793.40

For peer review only - http://bmjopen.bmj.com/site/about/guidelines.xhtml

Shayo et al 2017	Nurse Counsellors	Monthly salary	2012	819.44	1,875,051.02
Shayo et al 2017	Medical Doctors	Monthly salary	2012	1473.56	3,371,810.40
Shayo et al 2017	Medical Specialists	Monthly salary	2012	2721.79	6,228,019.13
Shayo et al 2017	Laboratory Scientific Officer	Monthly salary	2012	1106.07	2,530,924.80
Shayo et al 2017	Laboratory technician	Monthly salary	2012	819.44	1,875,051.02
Shayo et al 2017	Pharmacist	Monthly salary	2012	1106.07	2,530,924.80
Shayo et al 2017	Pharmacy technician	Monthly salary	2012	819.44	1,875,051.02

For peer review only

PRISMA 2020 Checklist

Section and Topic	Item #	Checklist item	Location where item is reported
**TITLE**			
Title	1	Identify the report as a systematic review.	Title page
**ABSTRACT**			
Abstract	2	See the PRISMA 2020 for Abstracts checklist.	Pages 2 & 3
**INTRODUCTION**			
Rationale	3	Describe the rationale for the review in the context of existing knowledge.	Page 5 & 6 Lines 79 - 91
Objectives	4	Provide an explicit statement of the objective(s) or question(s) the review addresses.	Page 6, Lines 93 - 94
**METHODS**			
Eligibility criteria	5	Specify the inclusion and exclusion criteria for the review and how studies were grouped for the syntheses.	Page 8, Lines 123-125
Information sources	6	Specify all databases, registers, websites, organisations, reference lists and other sources searched or consulted to identify studies. Specify the date when each source was last searched or consulted.	Page 6, Lines 105-106
Search strategy	7	Present the full search strategies for all databases, registers and websites, including any filters and limits used.	Supplementary 1
Selection process	8	Specify the methods used to decide whether a study met the inclusion criteria of the review, including how many reviewers screened each record and each report retrieved, whether they worked independently, and if applicable, details of automation tools used in the process.	Page 8, Lines 118-120
Data collection process	9	Specify the methods used to collect data from reports, including how many reviewers collected data from each report, whether they worked independently, any processes for obtaining or confirming data from study investigators, and if applicable, details of automation tools used in the process.	Page 8, Lines 128-131
Data items	10a	List and define all outcomes for which data were sought. Specify whether all results that were compatible with each outcome domain in each study were sought (e.g. for all measures, time points, analyses), and if not, the methods used to decide which results to collect.	Page 9, Lines 133-138
	10b	List and define all other variables for which data were sought (e.g. participant and intervention characteristics, funding sources). Describe any assumptions made about any missing or unclear information.	Page 8, Lines 128-131
Study risk of bias assessment	11	Specify the methods used to assess risk of bias in the included studies, including details of the tool(s) used, how many reviewers assessed each study and whether they worked independently, and if applicable, details of automation tools used in the process.	Page 9, Lines 142-146
Effect measures	12	Specify for each outcome the effect measure(s) (e.g. risk ratio, mean difference) used in the synthesis or presentation of results.	N/A
Synthesis methods	13a	Describe the processes used to decide which studies were eligible for each synthesis (e.g. tabulating the study intervention characteristics and comparing against the planned groups for each synthesis (item #5)).	Page 9, Lines 133-137
	13b	Describe any methods required to prepare the data for presentation or synthesis, such as handling of missing summary statistics, or data conversions.	N/A
	13c	Describe any methods used to tabulate or visually display results of individual studies and syntheses.	Page 9, Lines 133-137
	13d	Describe any methods used to synthesize results and provide a rationale for the choice(s). If meta-analysis was performed, describe the model(s), method(s) to identify the presence and extent of statistical heterogeneity, and software package(s) used.	N/A
	13e	Describe any methods used to explore possible causes of heterogeneity among study results (e.g. subgroup analysis, meta-regression).	N/A

For peer review only

For peer review only - http://bmjopen.bmj.com/site/about/guidelines.xhtml

PRISMA 2020 Checklist

Section and Topic	Item #	Checklist item	Location where item is reported
	13f	Describe any sensitivity analyses conducted to assess robustness of the synthesized results.	N/A
Reporting bias assessment	14	Describe any methods used to assess risk of bias due to missing results in a synthesis (arising from reporting biases).	N/A
Certainty assessment	15	Describe any methods used to assess certainty (or confidence) in the body of evidence for an outcome.	Page 9, Lines 142-146
**RESULTS**			
Study selection	16a	Describe the results of the search and selection process, from the number of records identified in the search to the number of studies included in the review, ideally using a flow diagram.	Figure 1
	16b	Cite studies that might appear to meet the inclusion criteria, but which were excluded, and explain why they were excluded.	N/A
Study characteristics	17	Cite each included study and present its characteristics.	Table 2
Risk of bias in studies	18	Present assessments of risk of bias for each included study.	Supplementary
Results of individual studies	19	For all outcomes, present, for each study: (a) summary statistics for each group (where appropriate) and (b) an effect estimate and its precision (e.g. confidence/credible interval), ideally using structured tables or plots.	Tables 3-6
Results of syntheses	20a	For each synthesis, briefly summarise the characteristics and risk of bias among contributing studies.	N/A
	20b	Present results of all statistical syntheses conducted. If meta-analysis was done, present for each the summary estimate and its precision (e.g. confidence/credible interval) and measures of statistical heterogeneity. If comparing groups, describe the direction of the effect.	N/A
	20c	Present results of all investigations of possible causes of heterogeneity among study results.	N/A
	20d	Present results of all sensitivity analyses conducted to assess the robustness of the synthesized results.	N/A
Reporting biases	21	Present assessments of risk of bias due to missing results (arising from reporting biases) for each synthesis assessed.	N/A
Certainty of evidence	22	Present assessments of certainty (or confidence) in the body of evidence for each outcome assessed.	N/A
**DISCUSSION**			
Discussion	23a	Provide a general interpretation of the results in the context of other evidence.	Pages 40-44, Lines 294-369
	23b	Discuss any limitations of the evidence included in the review.	Pages 45-46, Lines 381- 391
	23c	Discuss any limitations of the review processes used.	Pages 45-46, Lines 381- 391
	23d	Discuss implications of the results for practice, policy, and future research.	Page 46, Lines 395-402
**OTHER INFORMATION**			
Registration and protocol	24a	Provide registration information for the review, including register name and registration number, or state that the review was not registered.	Page 6, Line 100
	24b	Indicate where the review protocol can be accessed, or state that a protocol was not prepared.	Page 6, Lines 100-101

For peer review only

For peer review only - http://bmjopen.bmj.com/site/about/guidelines.xhtml

PRISMA 2020 Checklist

Section and Topic	Item #	Checklist item	Location where item is reported
	24c	Describe and explain any amendments to information provided at registration or in the protocol.	N/A
Support	25	Describe sources of financial or non-financial support for the review, and the role of the funders or sponsors in the review.	Page 58, Lines 579-580
Competing interests	26	Declare any competing interests of review authors.	Page 58, Line 575
Availability of data, code and other materials	27	Report which of the following are publicly available and where they can be found: template data collection forms; data extracted from included studies; data used for all analyses; analytic code; any other materials used in the review.	Available

Based on Page MJ, McKenzie JE, Bossuyt PM, Boutron I, Hoffmann TC, Mulrow CD, et al. The PRISMA 2020 statement: an updated guideline for reporting systematic reviews. BMJ 2021;372:n71. doi: 10.1136/bmj.n71

For peer review only
